# Supplementary material for: Ginsenoside panaxatriol reverses TNBC paclitaxel resistance by inhibiting the IRAK1/NF-κB and ERK pathways
Source: PeerJ. 2020 Jun 3;8:e9281. doi: 10.7717/peerj.9281 (PMC7275687; doi:10.7717/peerj.9281)
Supplement: Supplemental Information 1 [file peerj-08-9281-s001.docx]

***Fig. S1* Effects of PTX on MB231-PT cells viability**

| A | B |
| --- | --- |
| 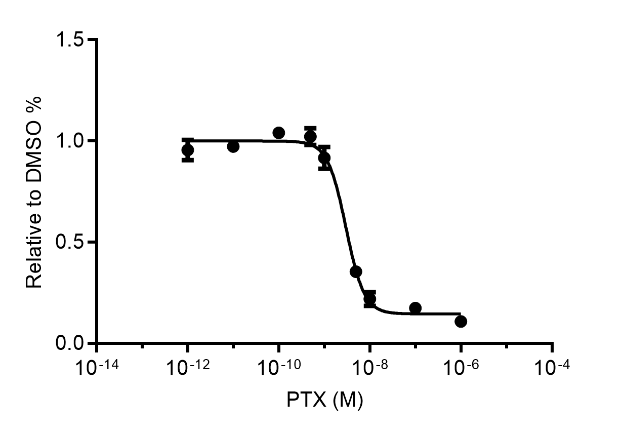 | 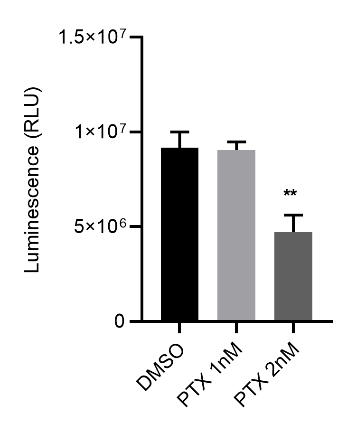 |

(A-B) Cells were treated with different concentration of PTX for 4 days. **P < 0.01, P-values were calculated with t test.

***Fig. S2* Compare of IRAK1/4 inh, GRg3 and GPT on MB231-PR cells viability**


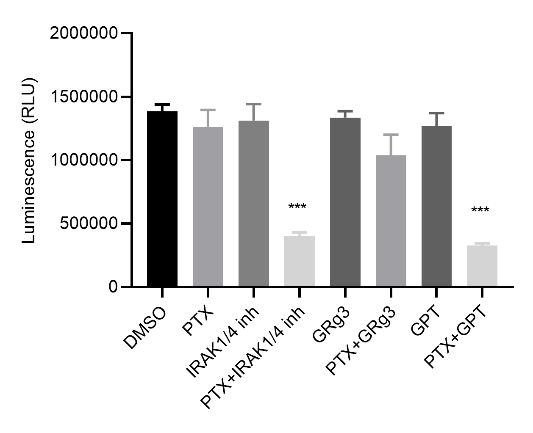


Cells were treated with DMSO, 75 nM PTX, 10 μM IRAK1/4 inh, 10 μM GRg3, 10 μM GPT, or combination for 4 days. ***P < 0.001. P-values were calculated with t test.

***Fig. S3* Raw data for Colony formation assay (article Fig 1D)**

***
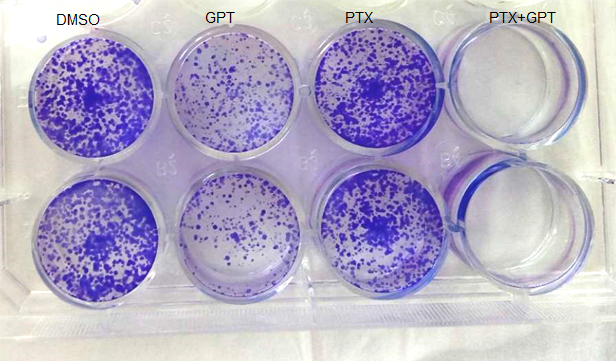
***

***Fig. S4* Raw data for FACS**


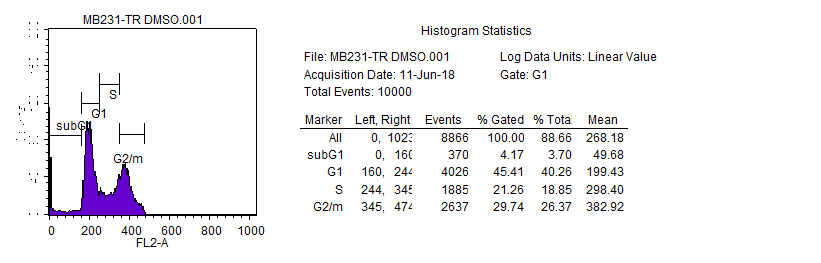


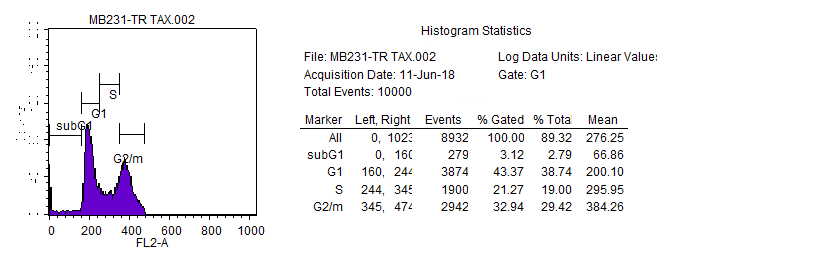


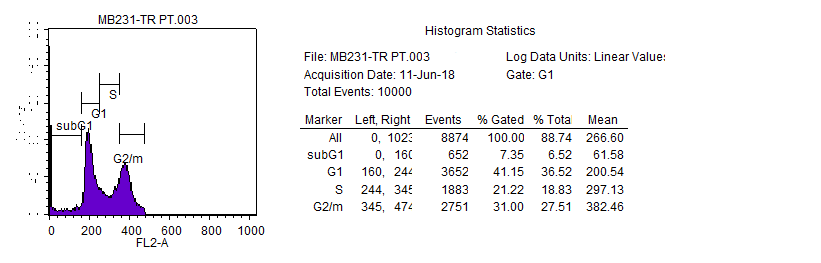


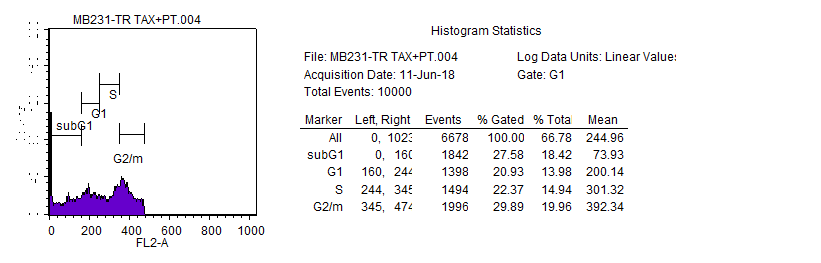


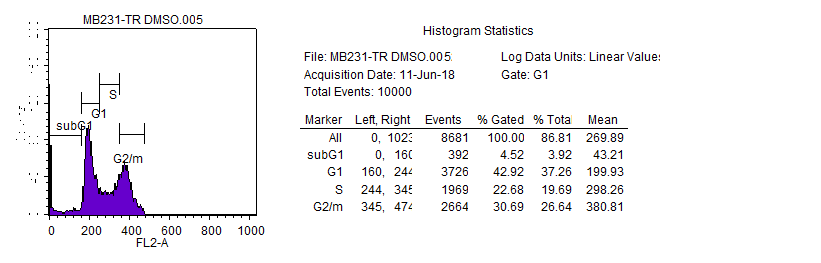


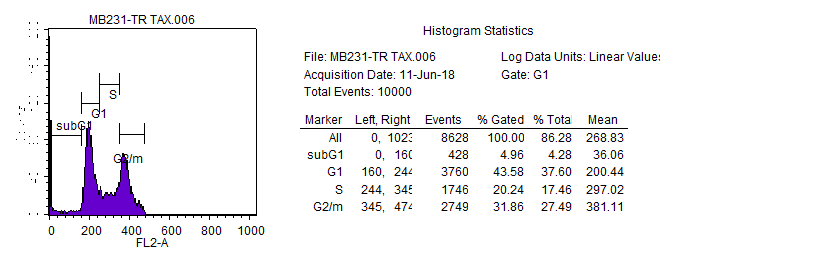


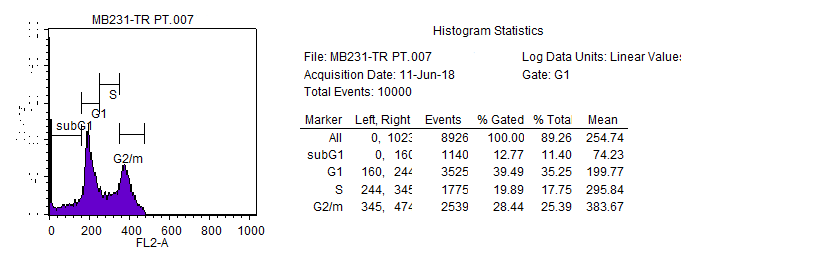


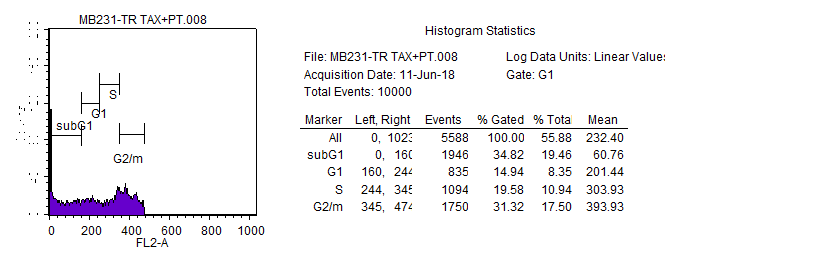


***Fig. S5* Raw data for western blots (article Fig 2A)**

**p-IRAK1 S376**


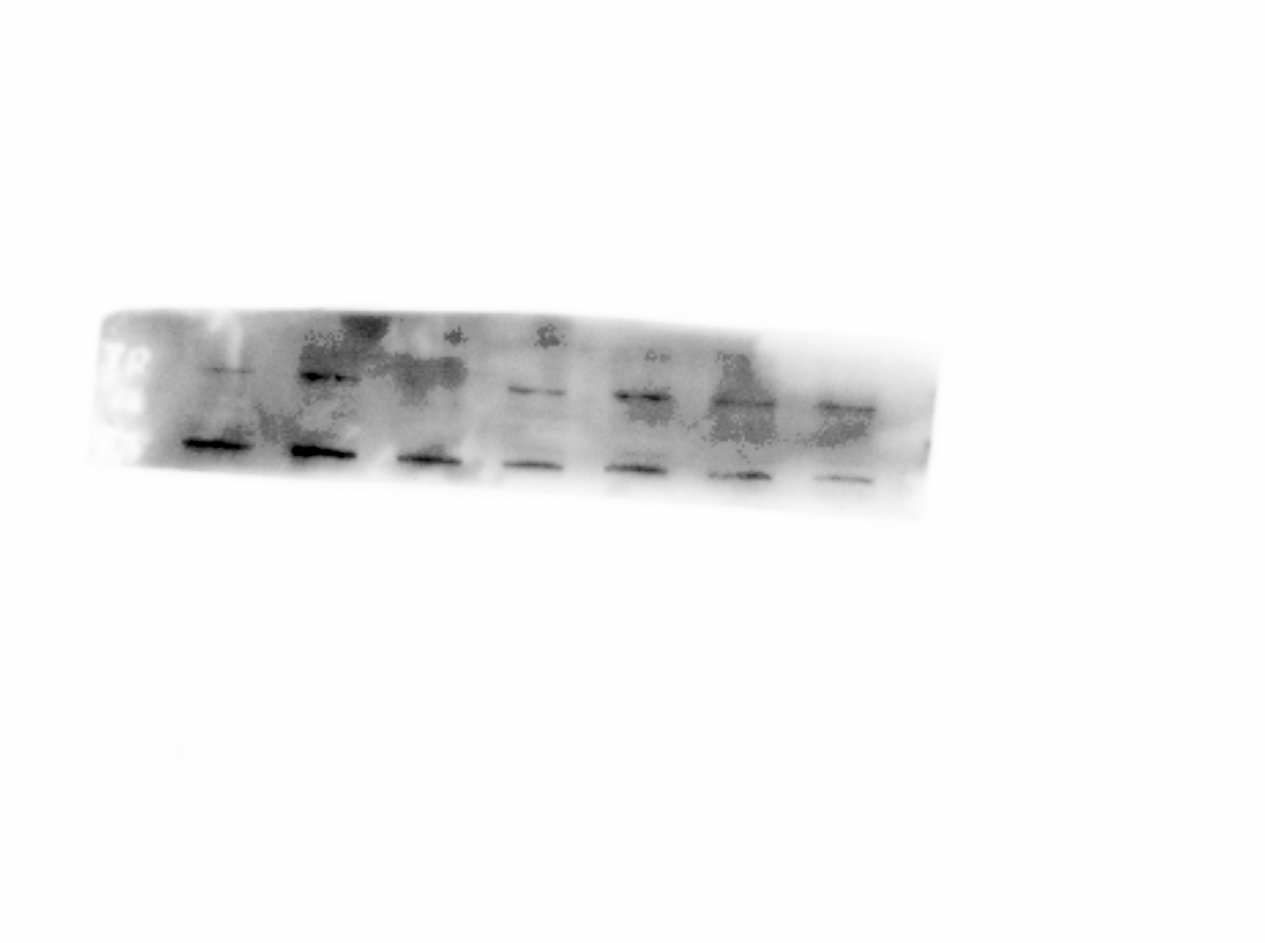


PTX+GPT 20

PTX+GPT 10

PTX+GPT 5

PTX+GPT 2.5

GPT

PTX

DMSO

**IRAK1**


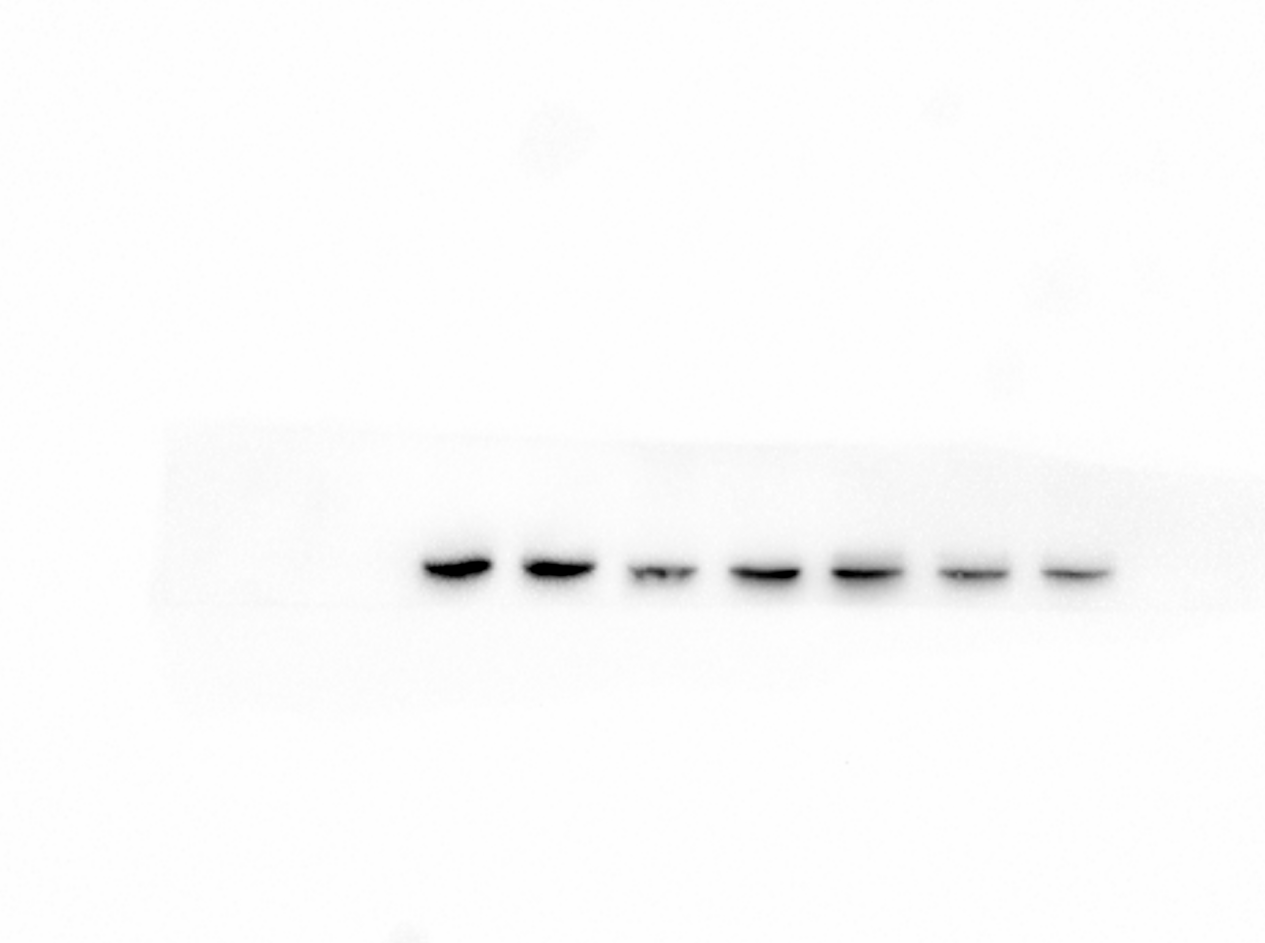


**p-P65 S536**


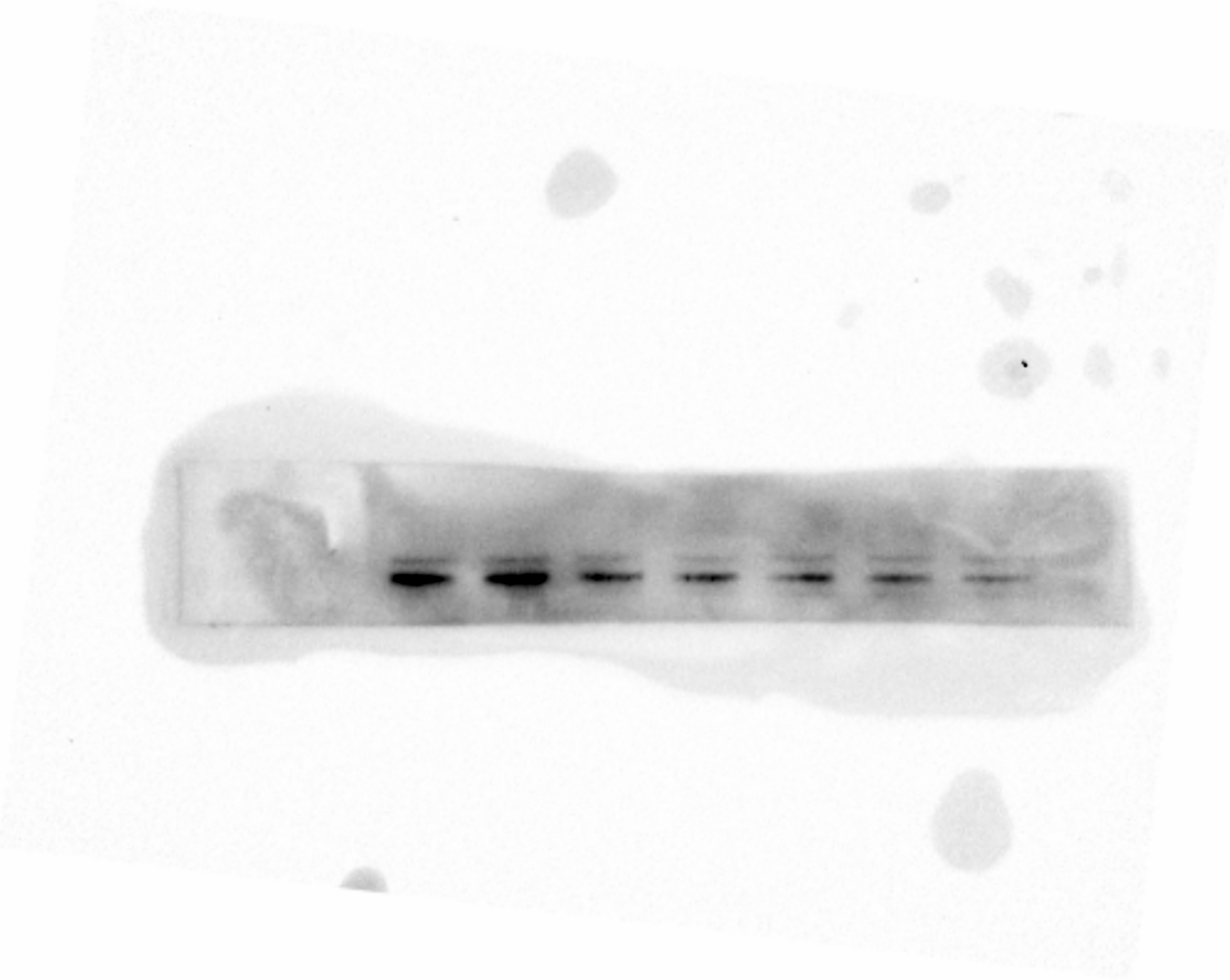


**P65**


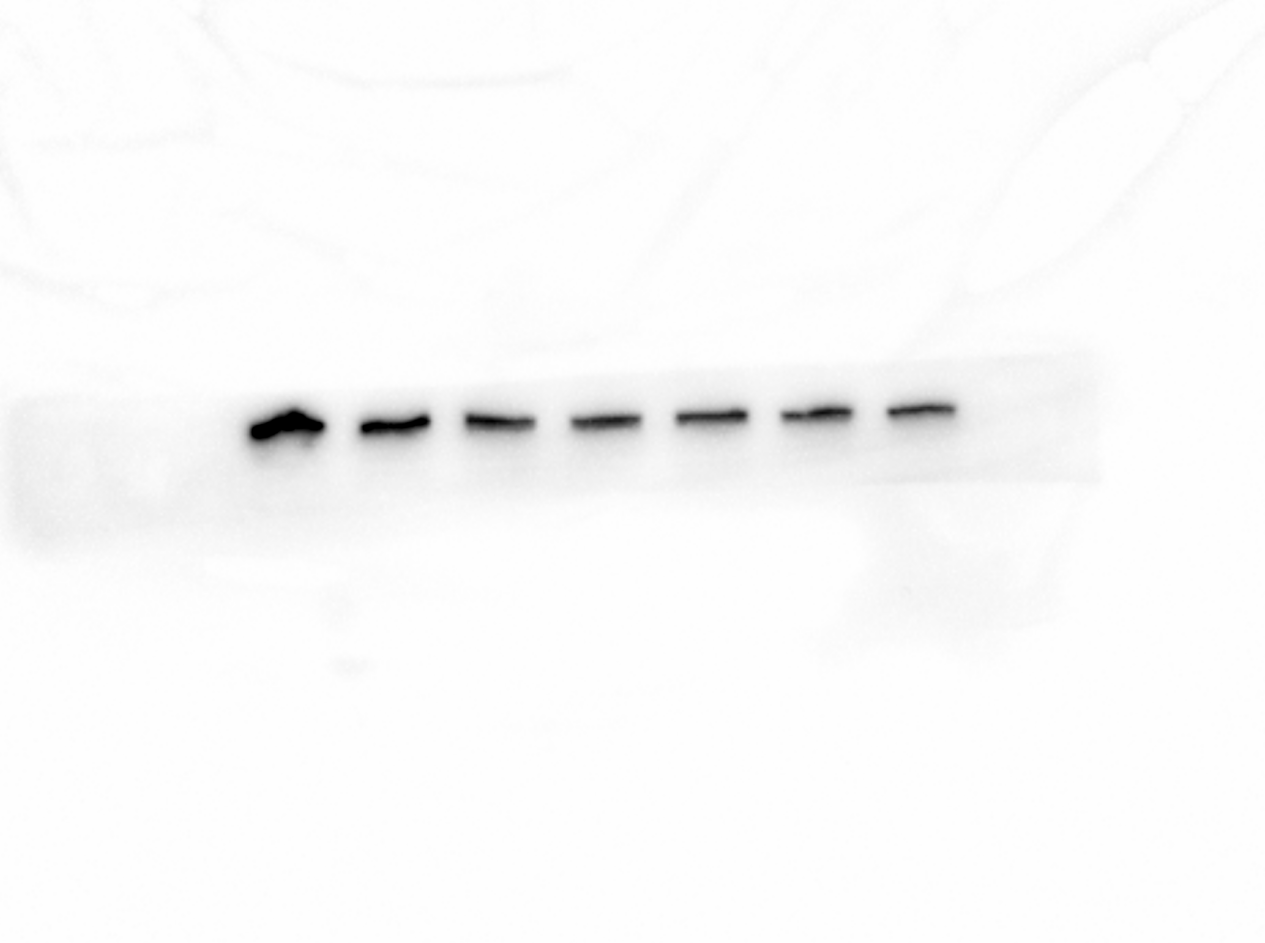
**p-ERK1/2**


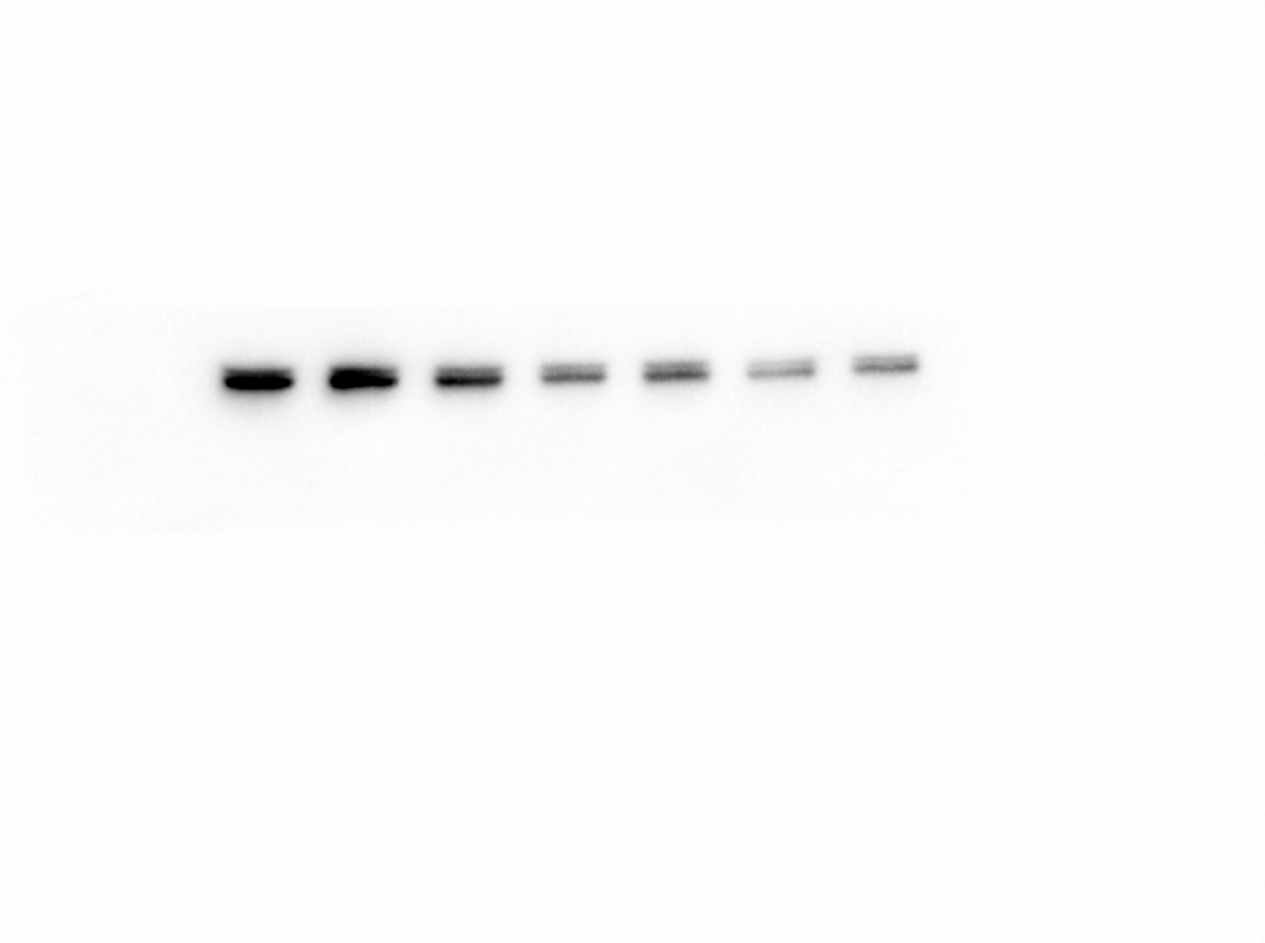


**ERK1/2**


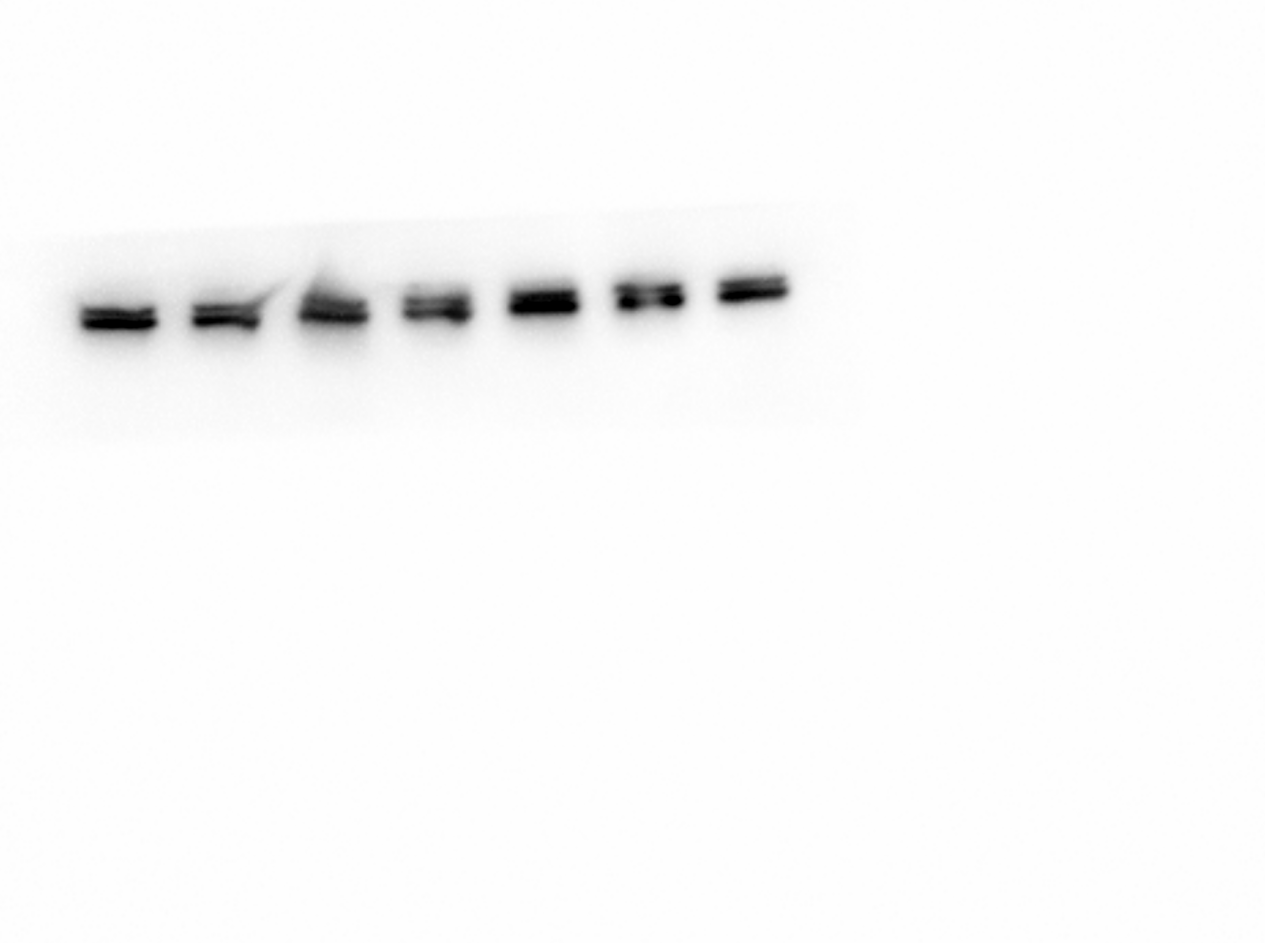


**BAX**


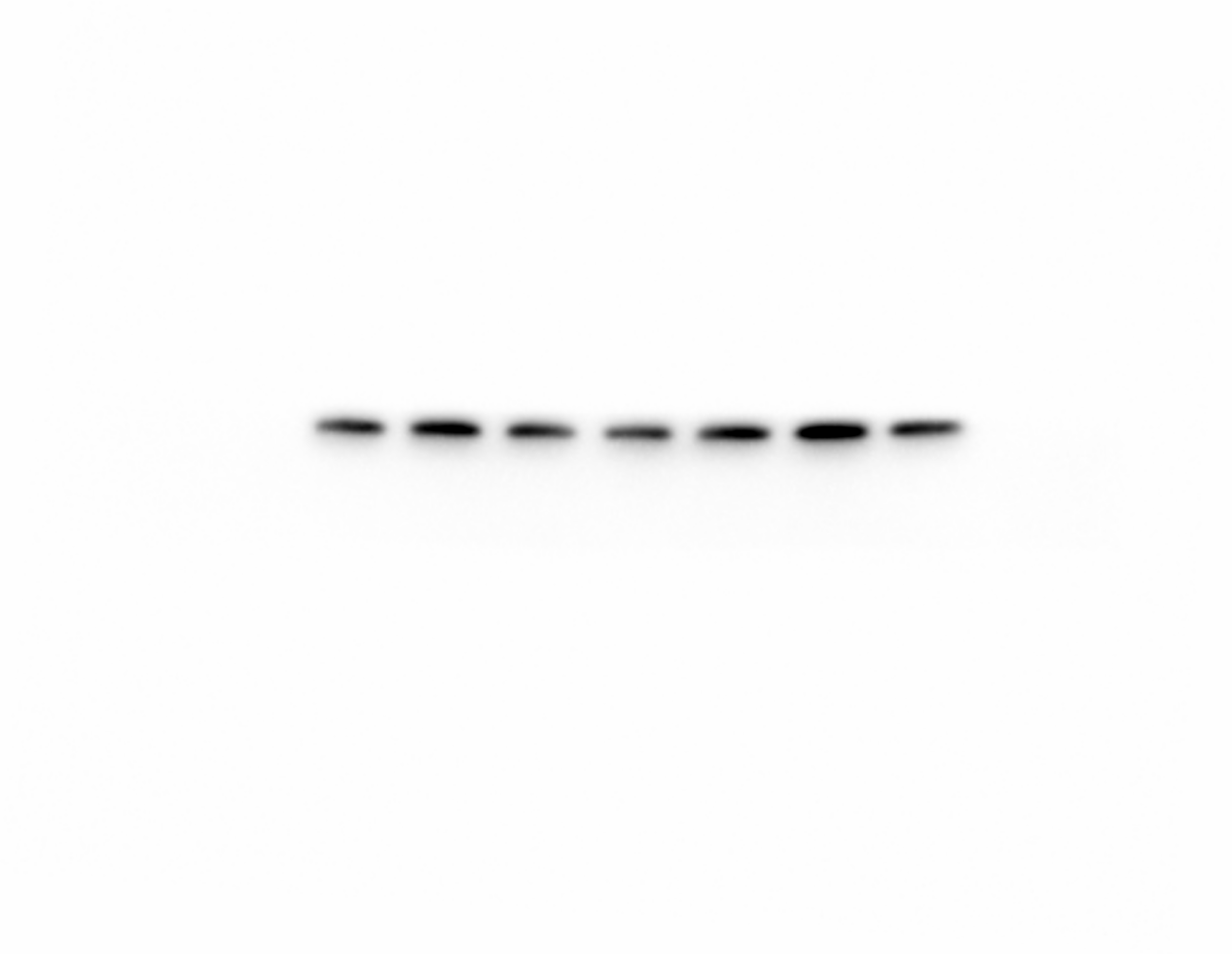


**BCL-2**


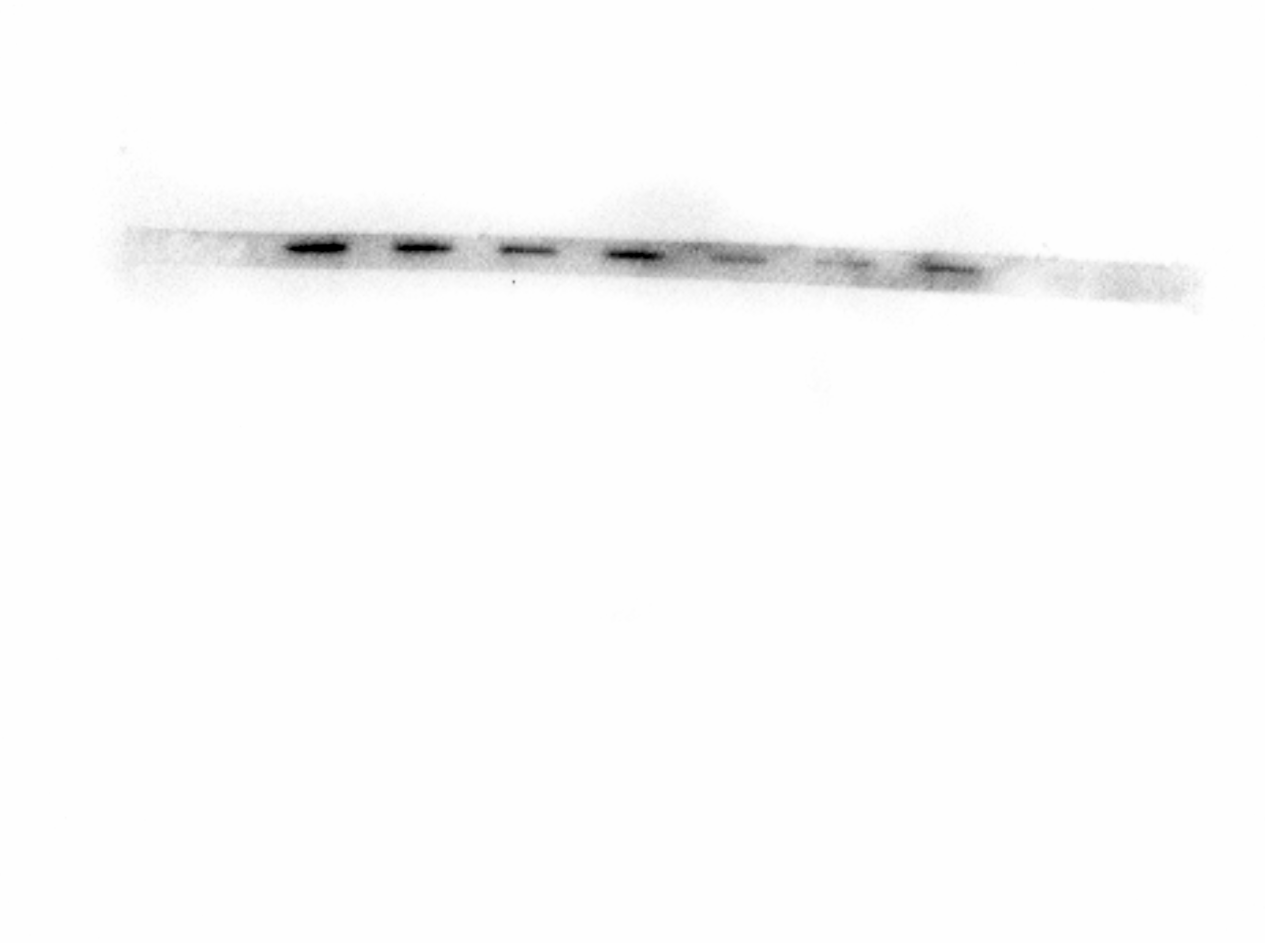


**MCL-1**


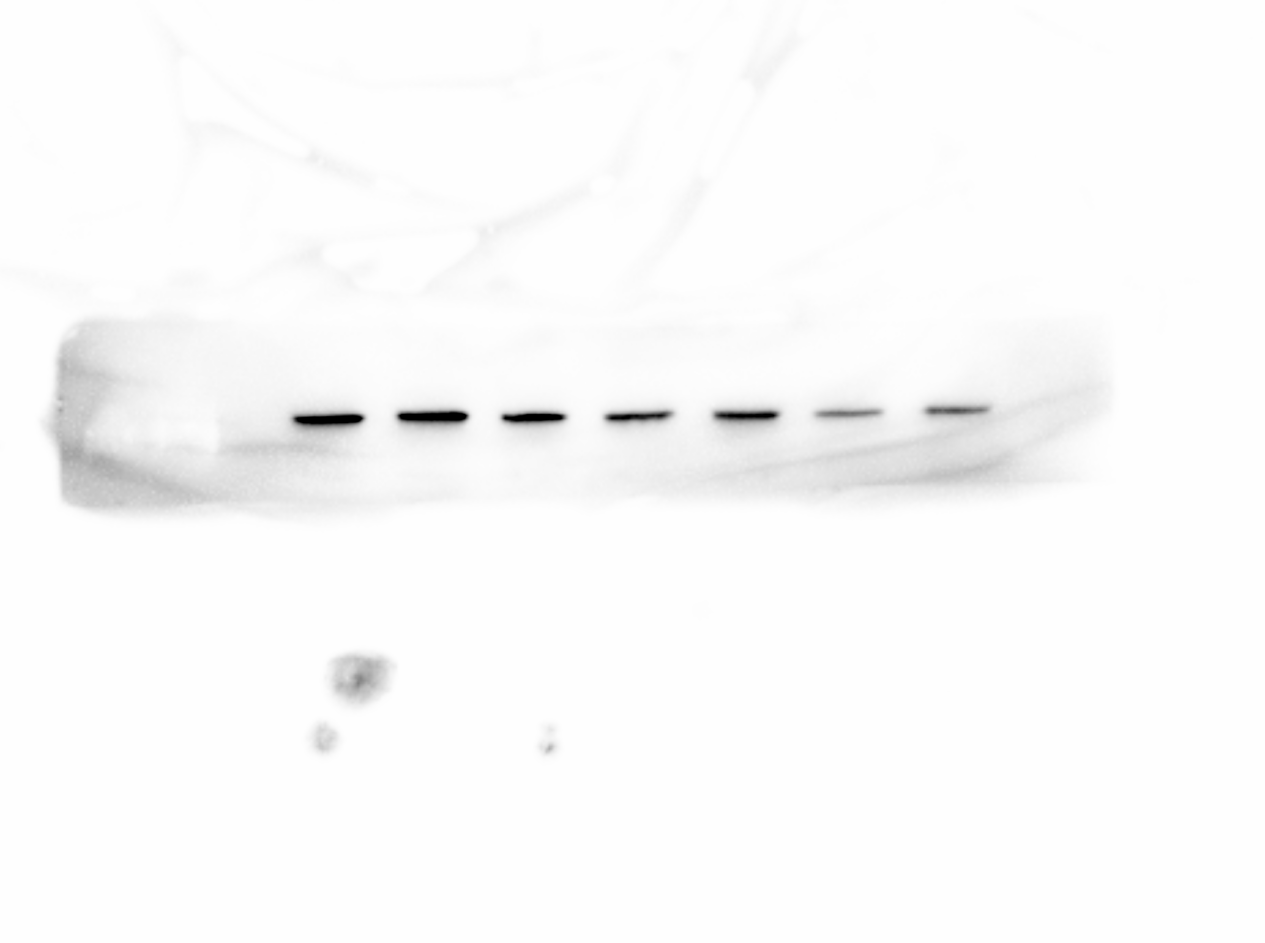


**beta-actin**


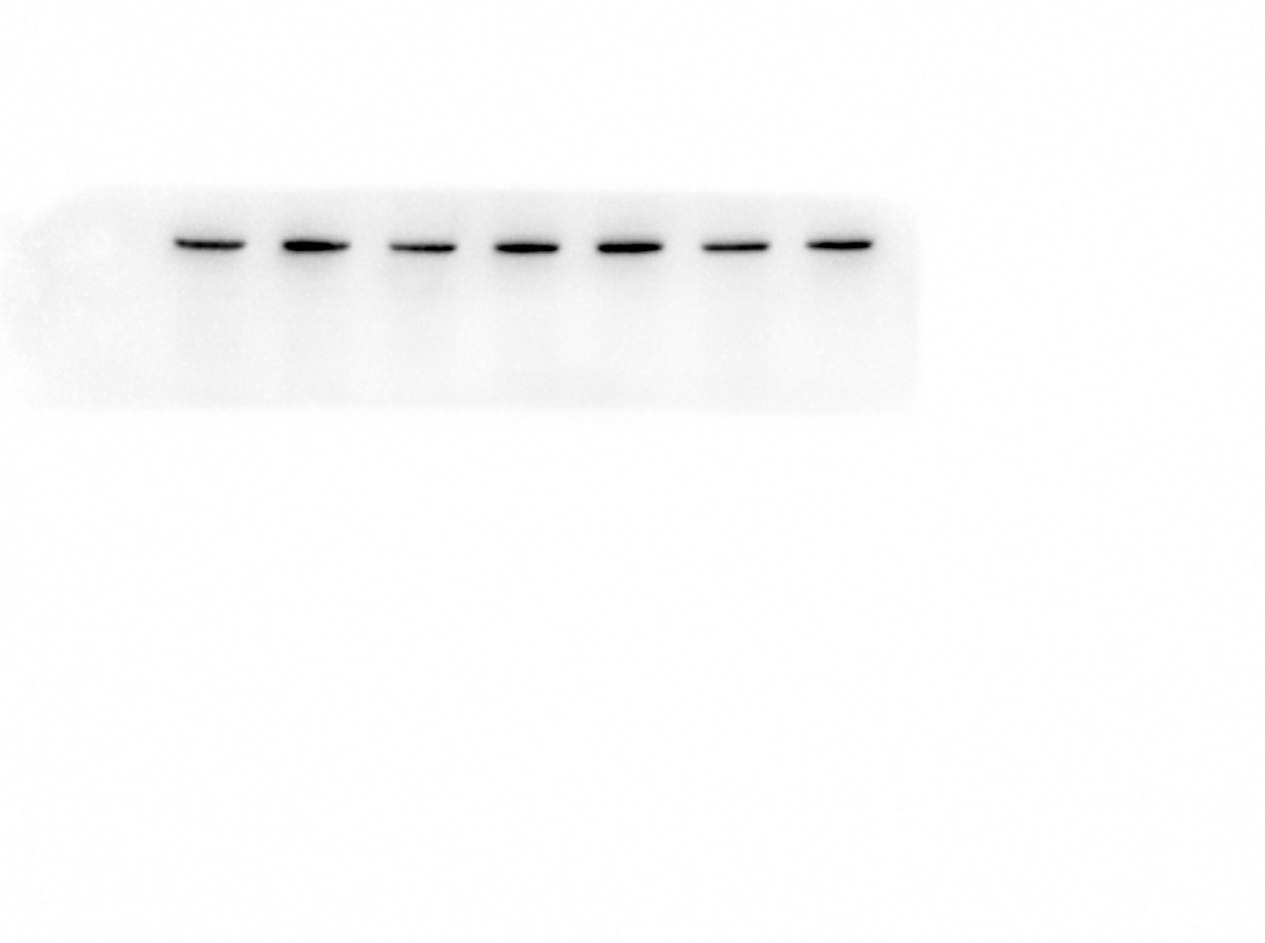


**IκB-alpha**

**
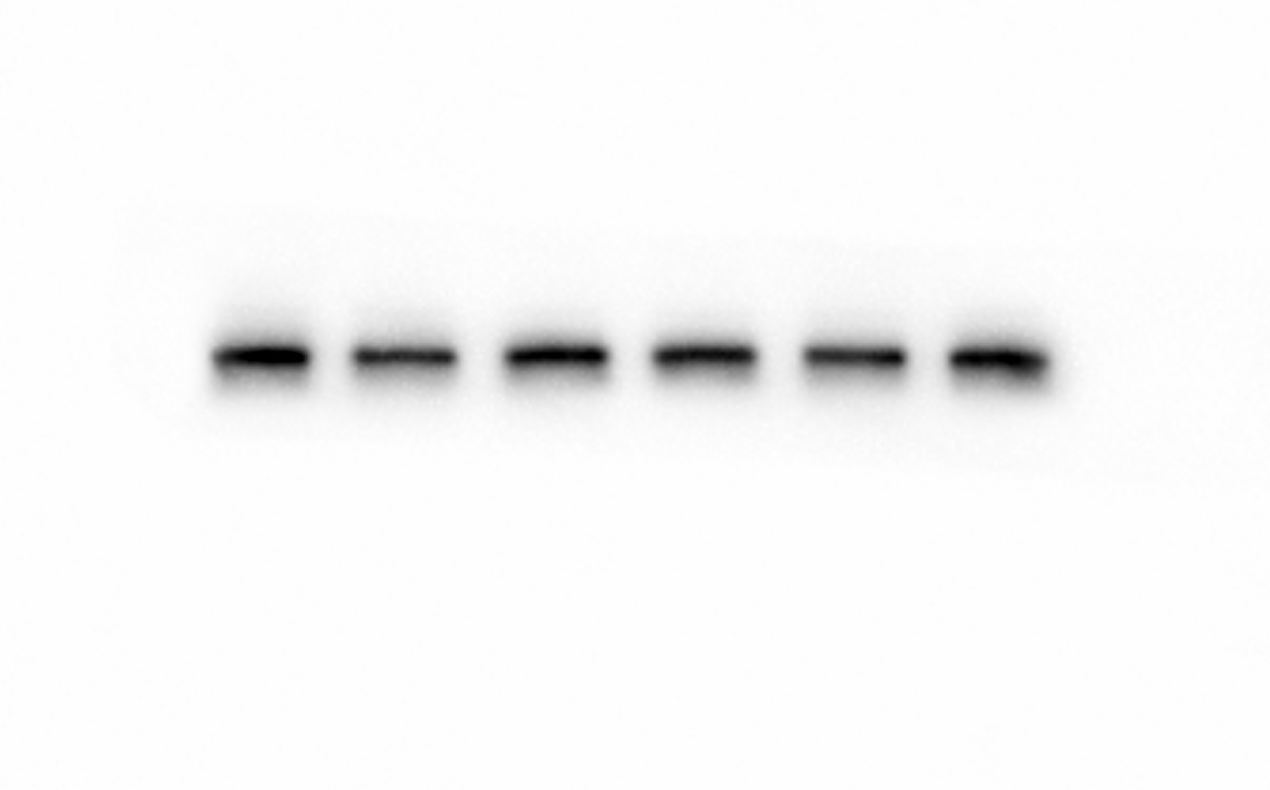
**

***Fig. S6* Raw data for western blots (article Fig 2B 24 h)**

**p-IRAK1 S376**


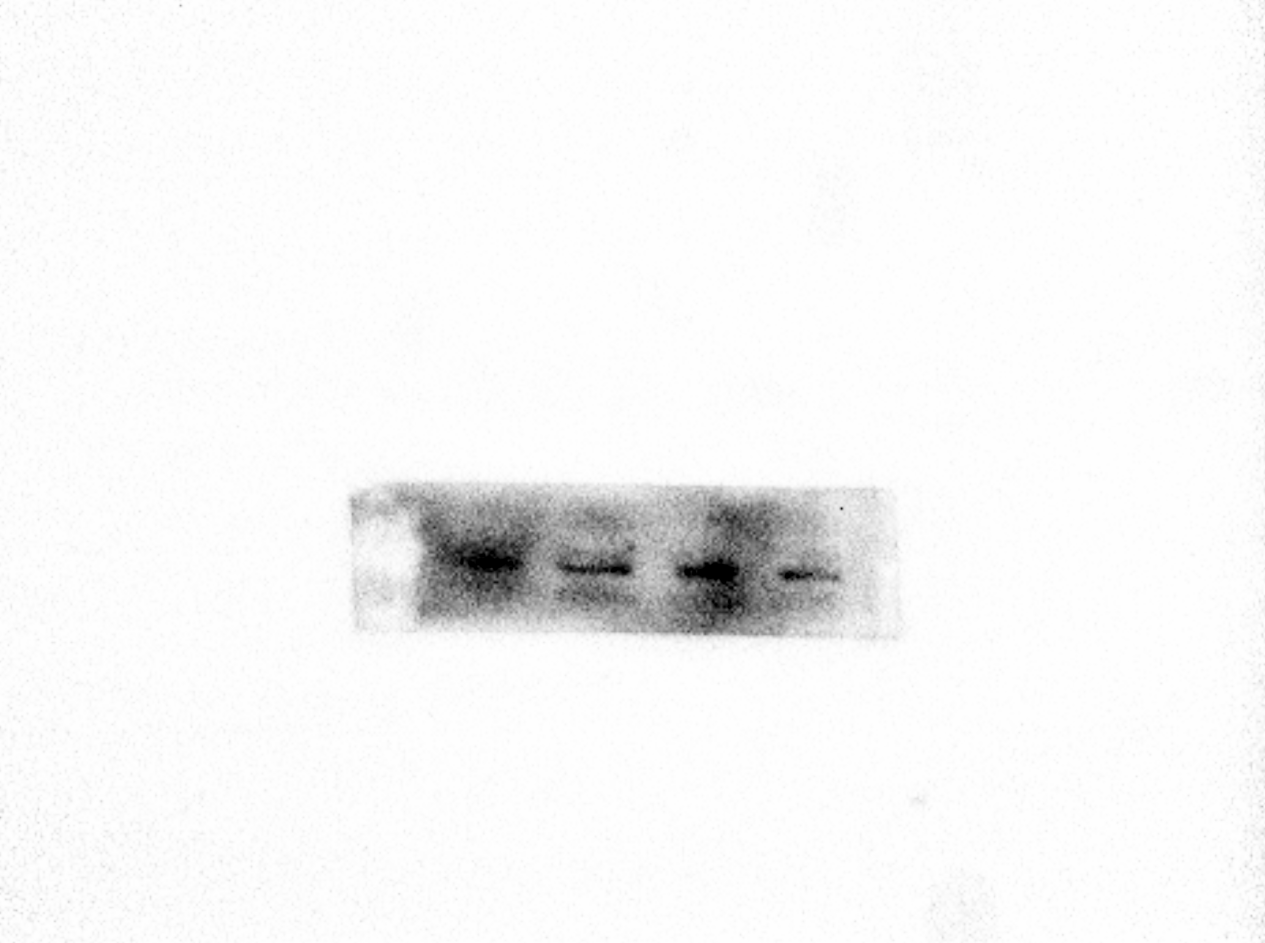


PTX

DMSO

PTX+GPT

GPT

**IRAK1**


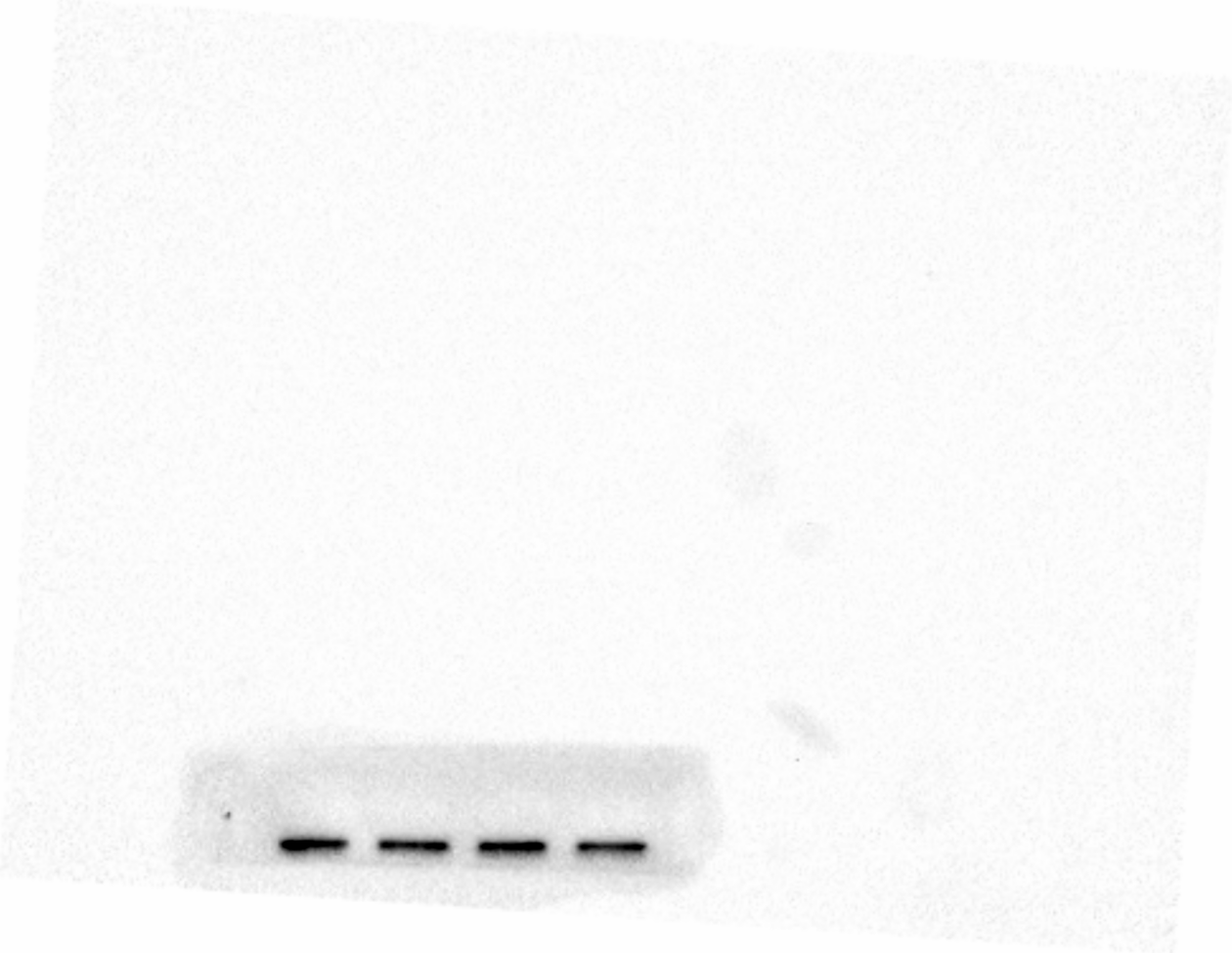


**p-P65 S536**


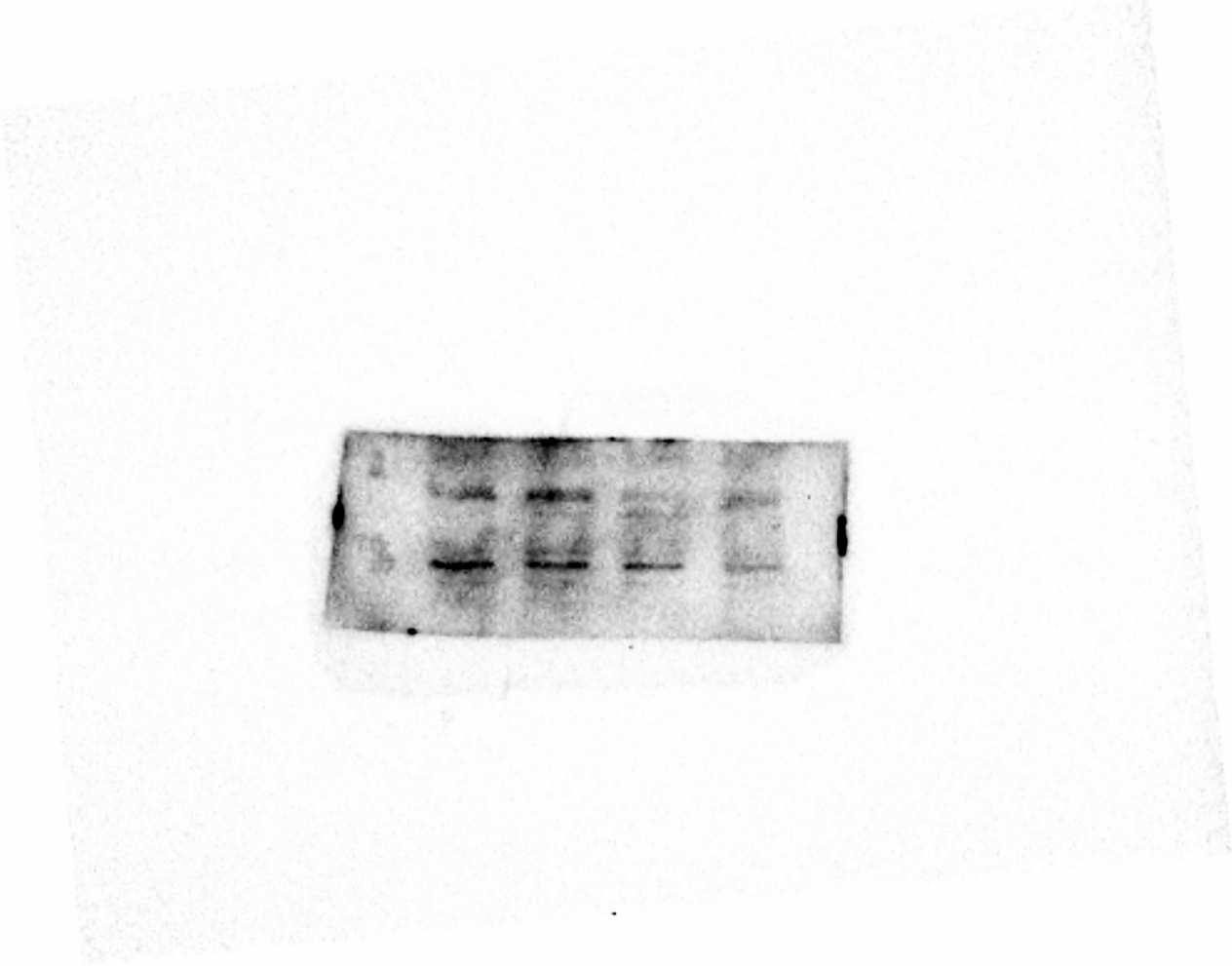


**P65**


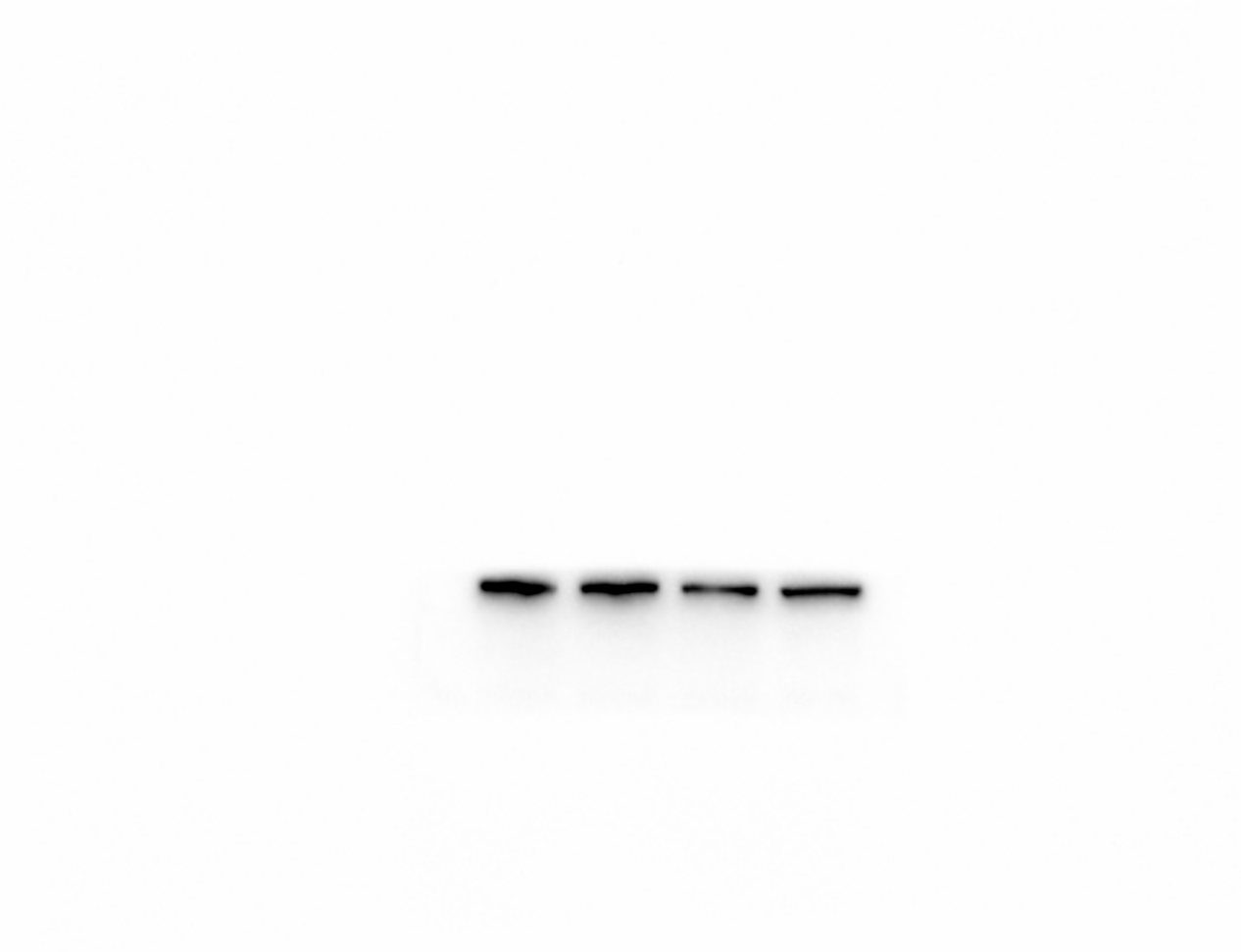


**p-ERK1/2**


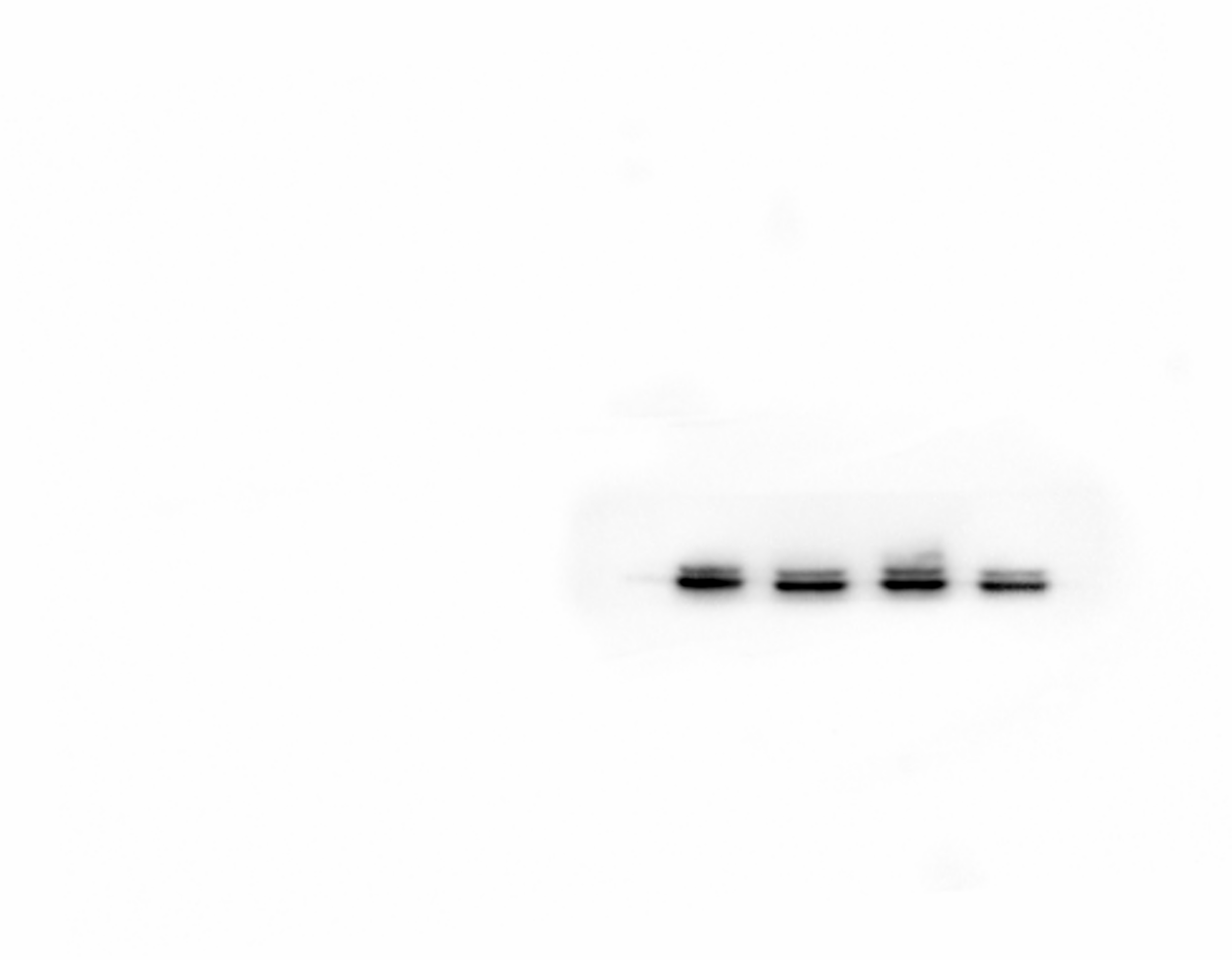


**ERK1/2**


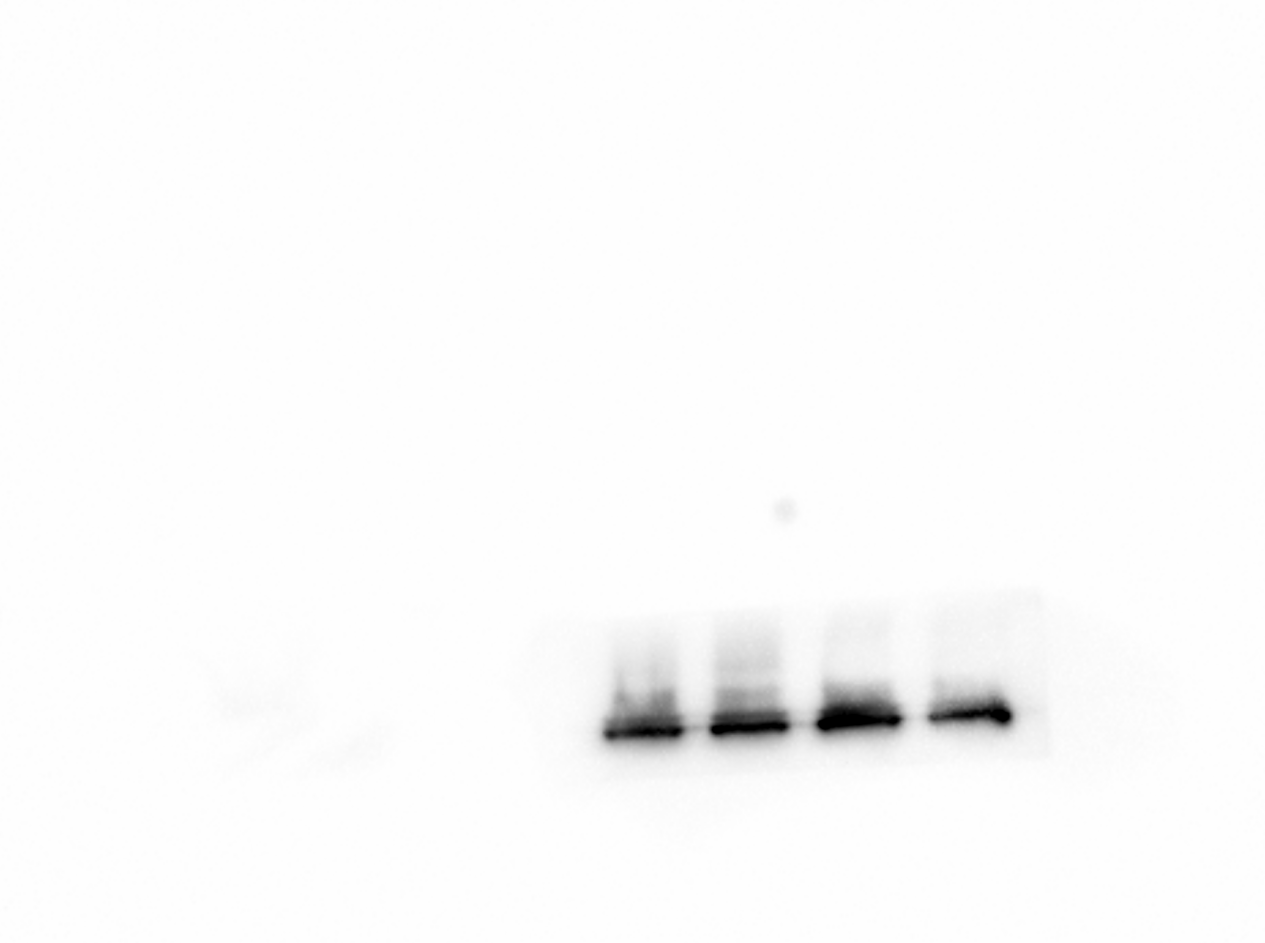


**BAX**


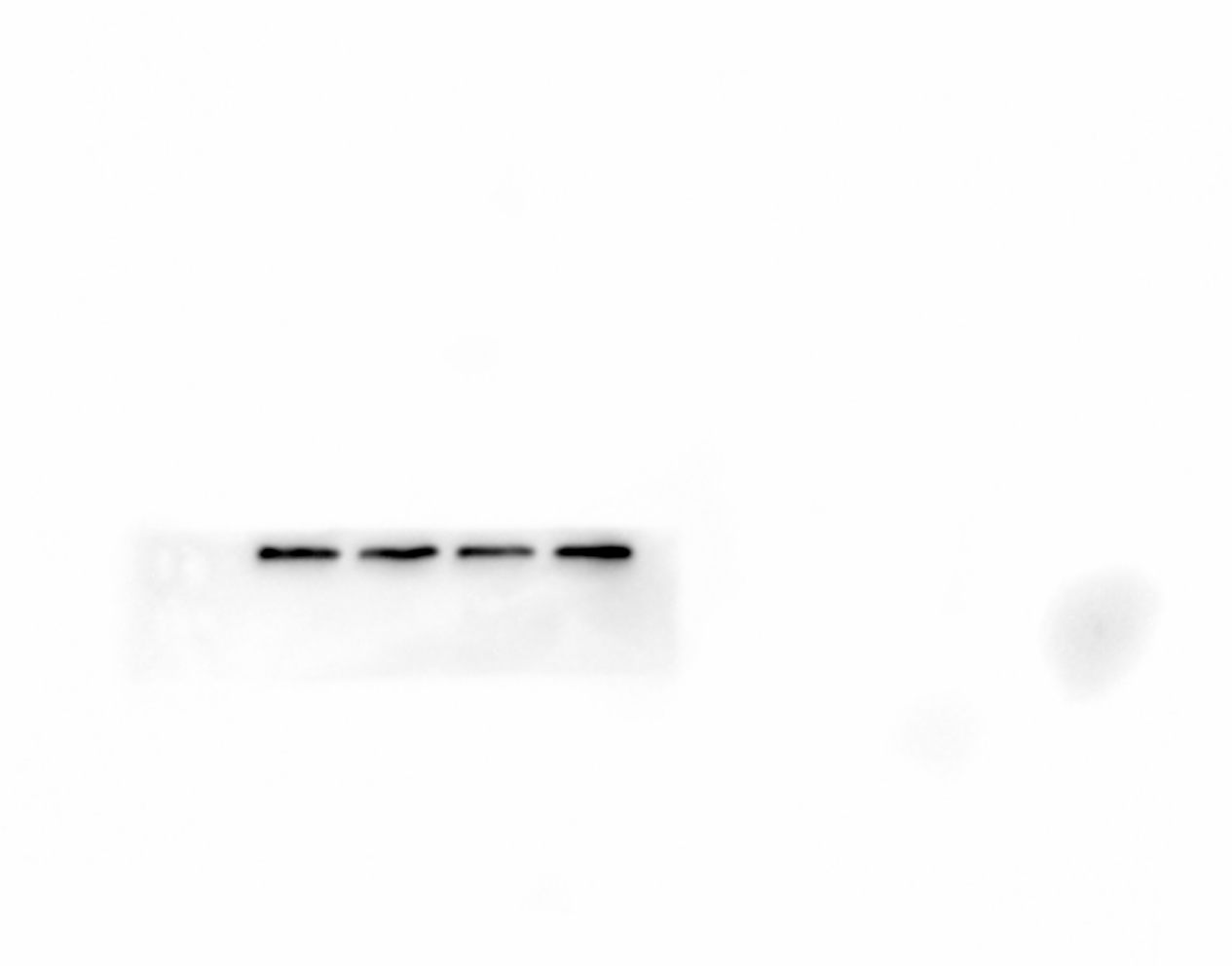


**BCL-2**


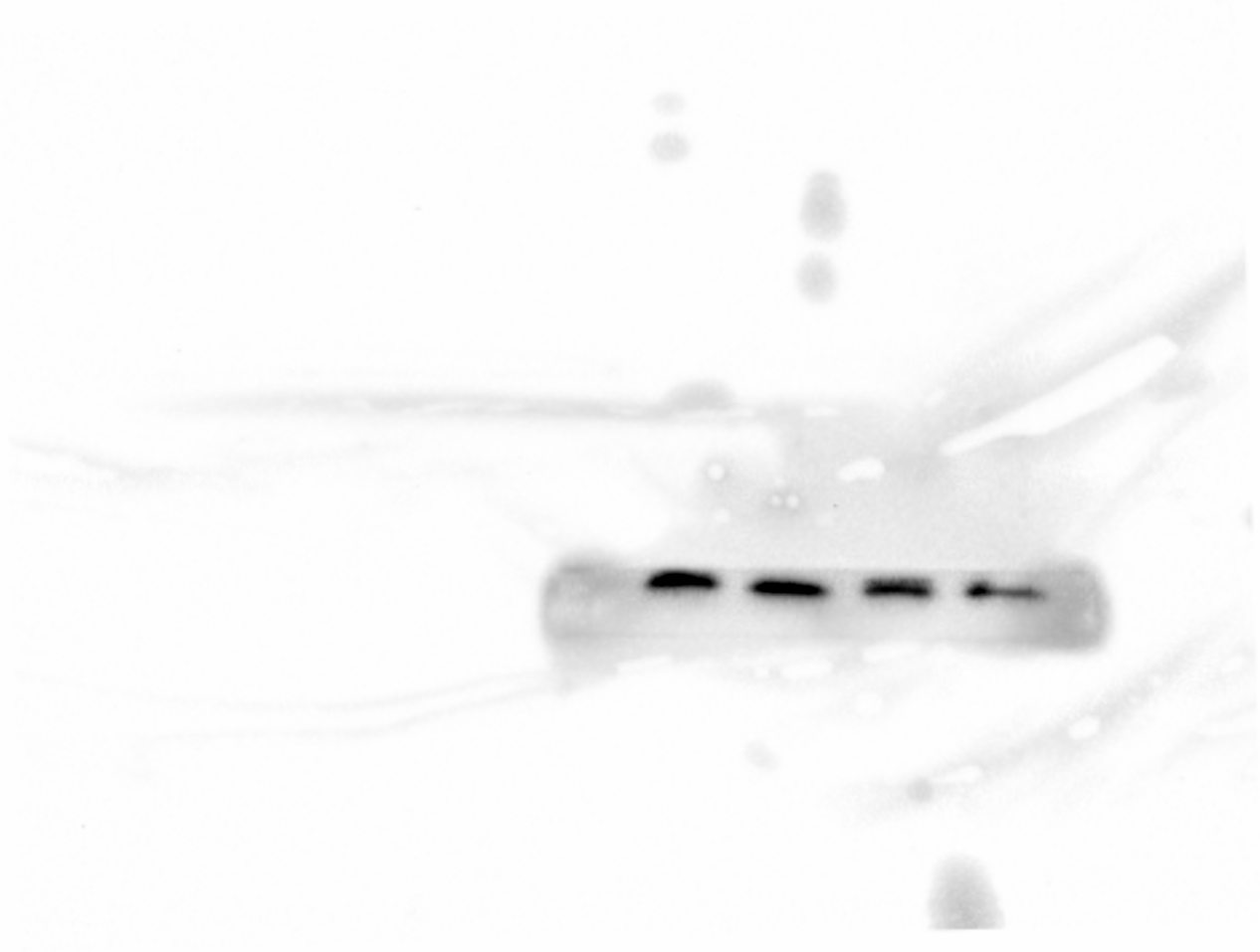


**MCL-1**


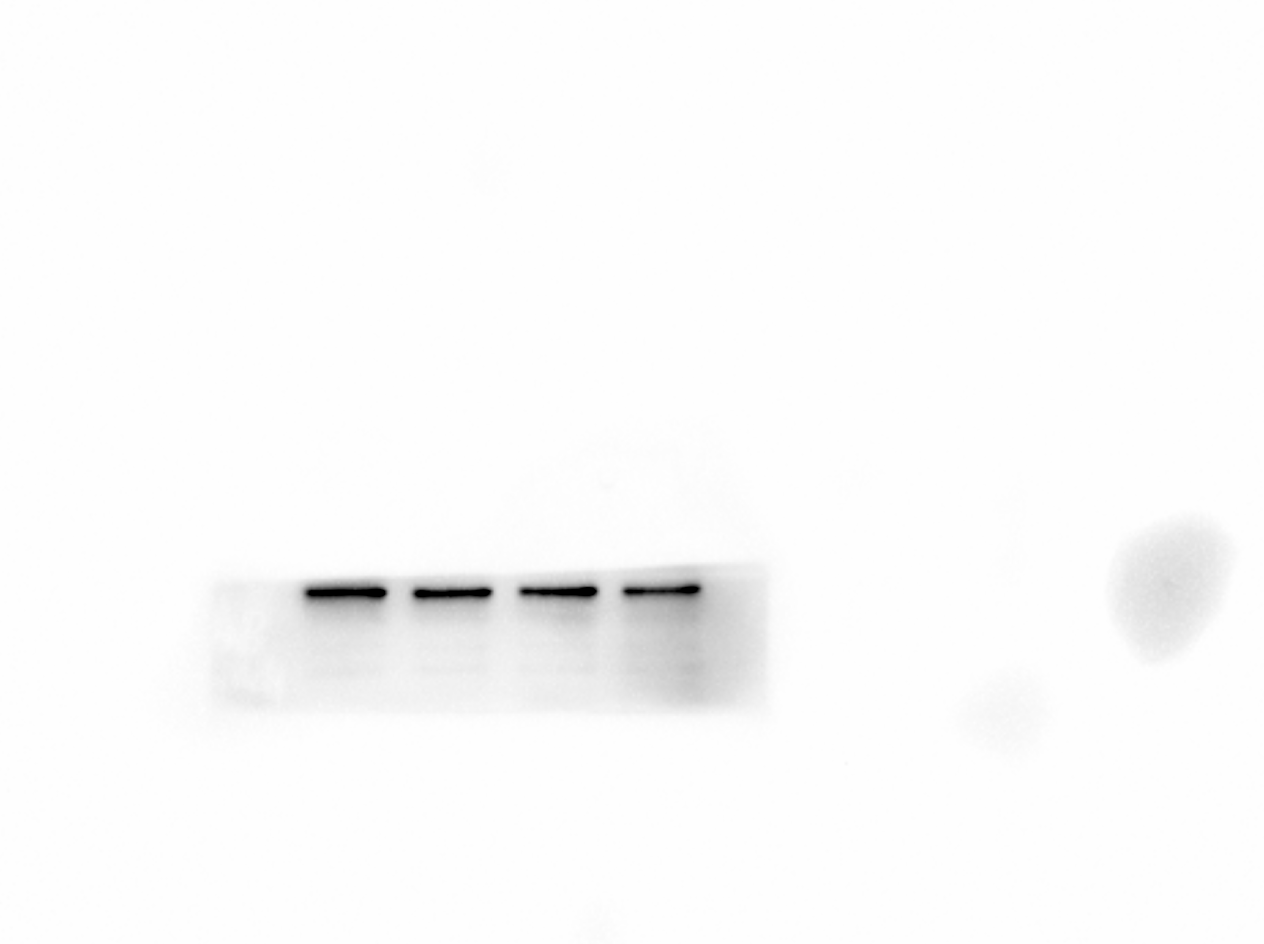


**beta-actin**


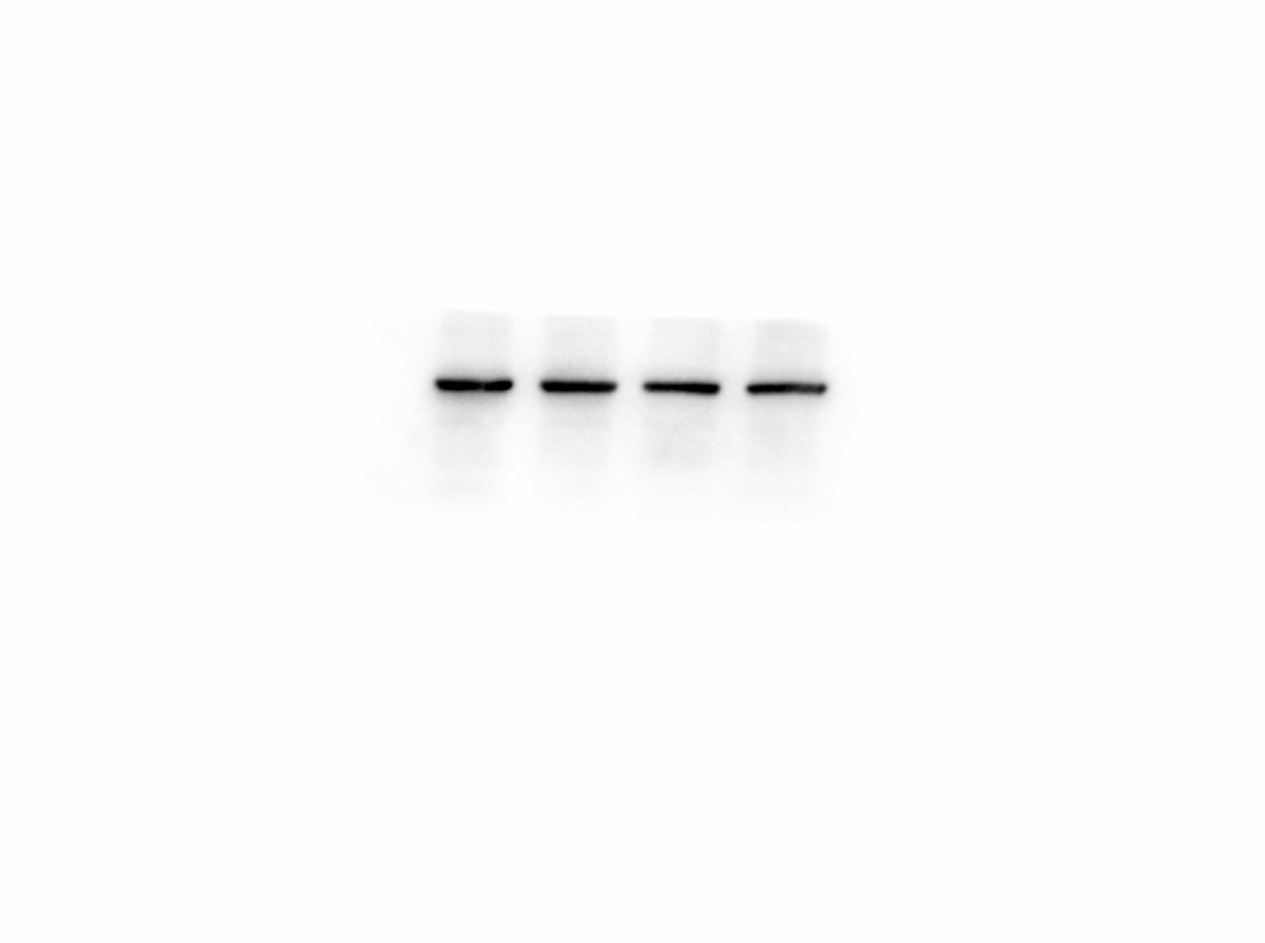


**IκB-alpha**

**
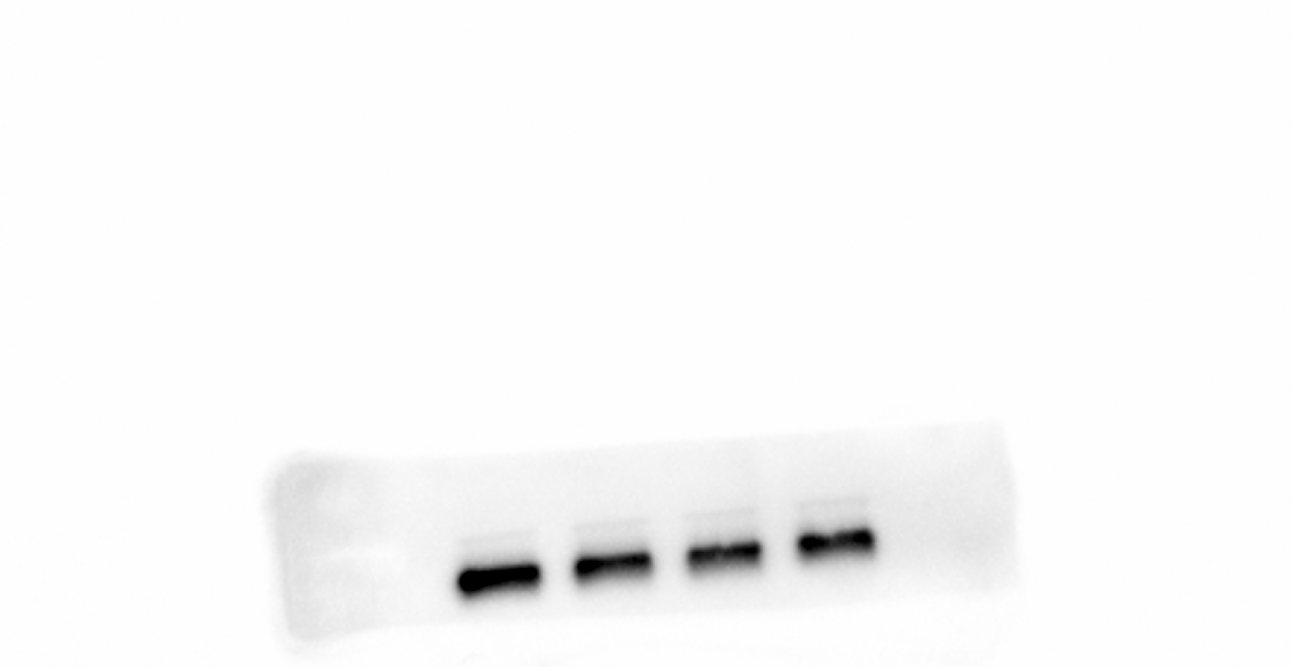
**

***Fig. S7* Raw data for western blots (article Fig 2B 48 h)**

**p-IRAK1 S376**


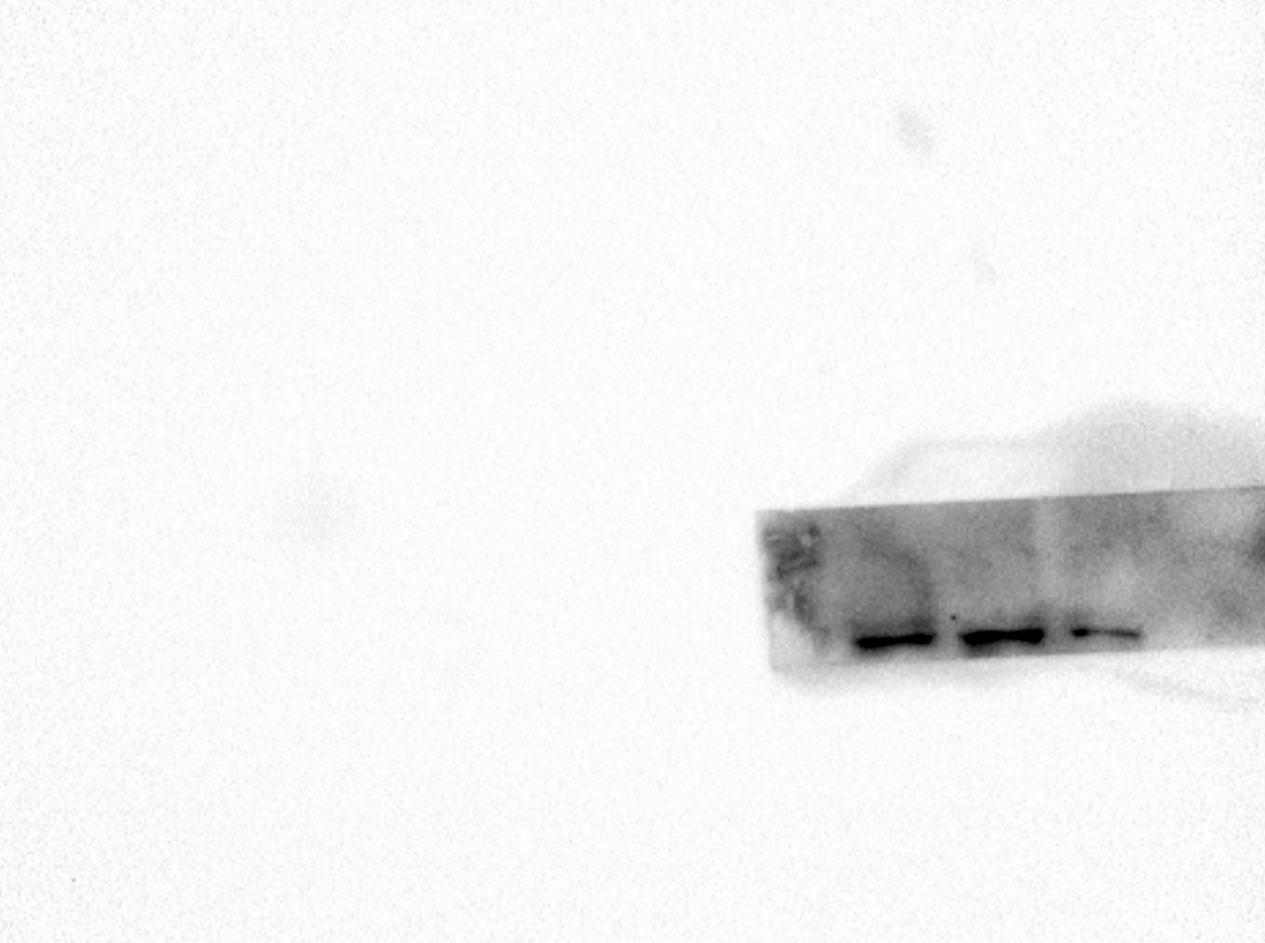


**IRAK1**


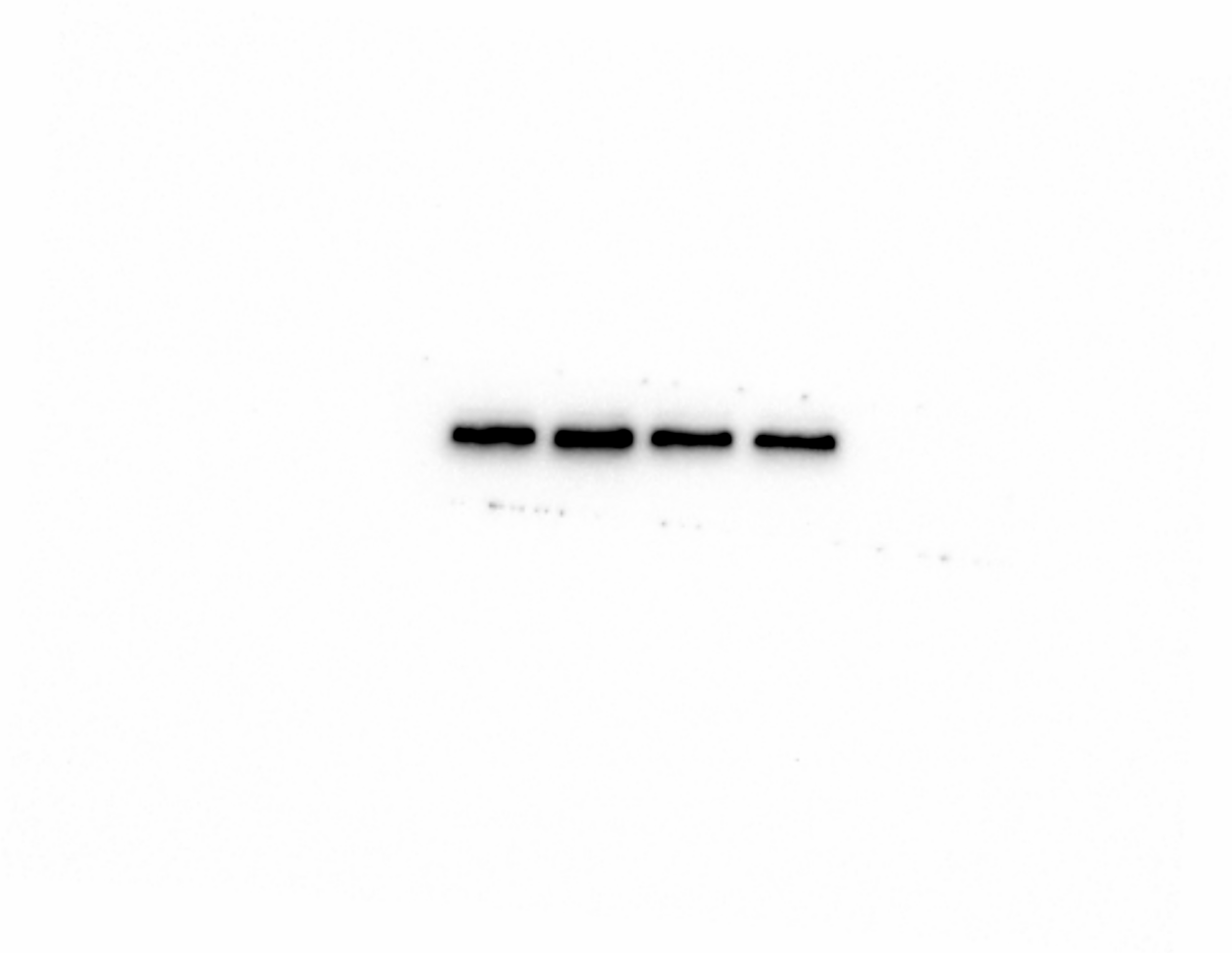


**p-P65 S536**


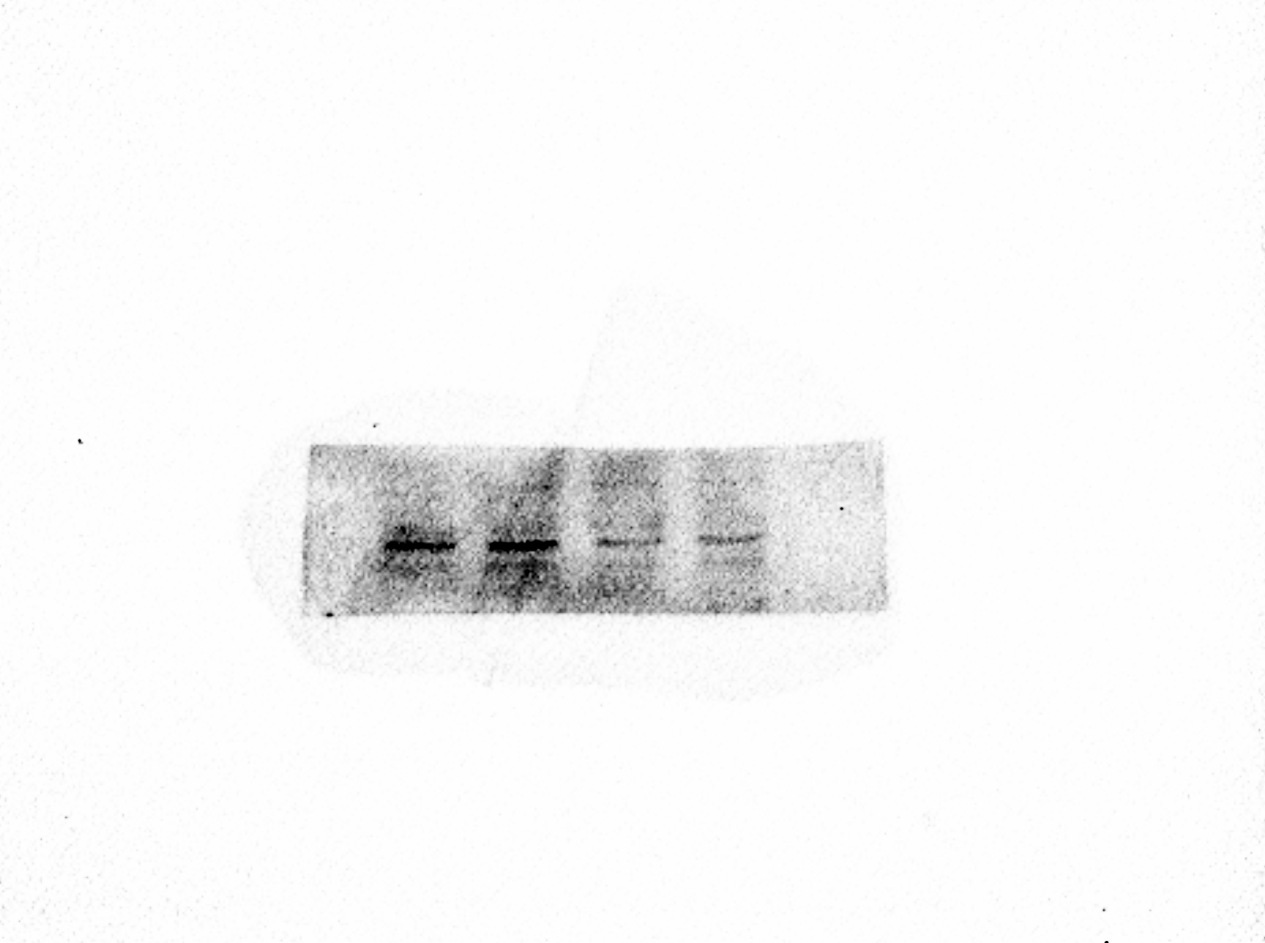


**P65**


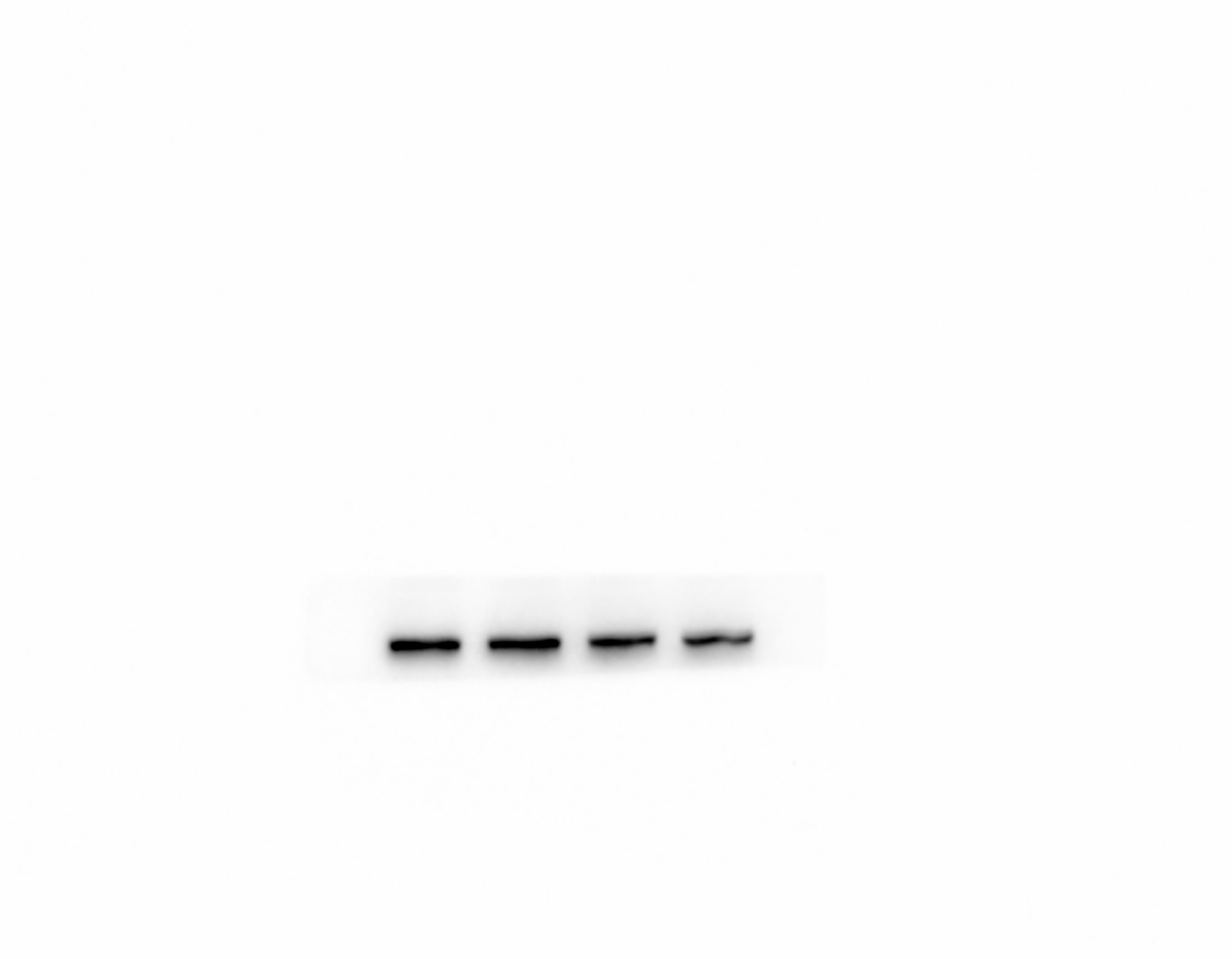


**p-ERK1/2**


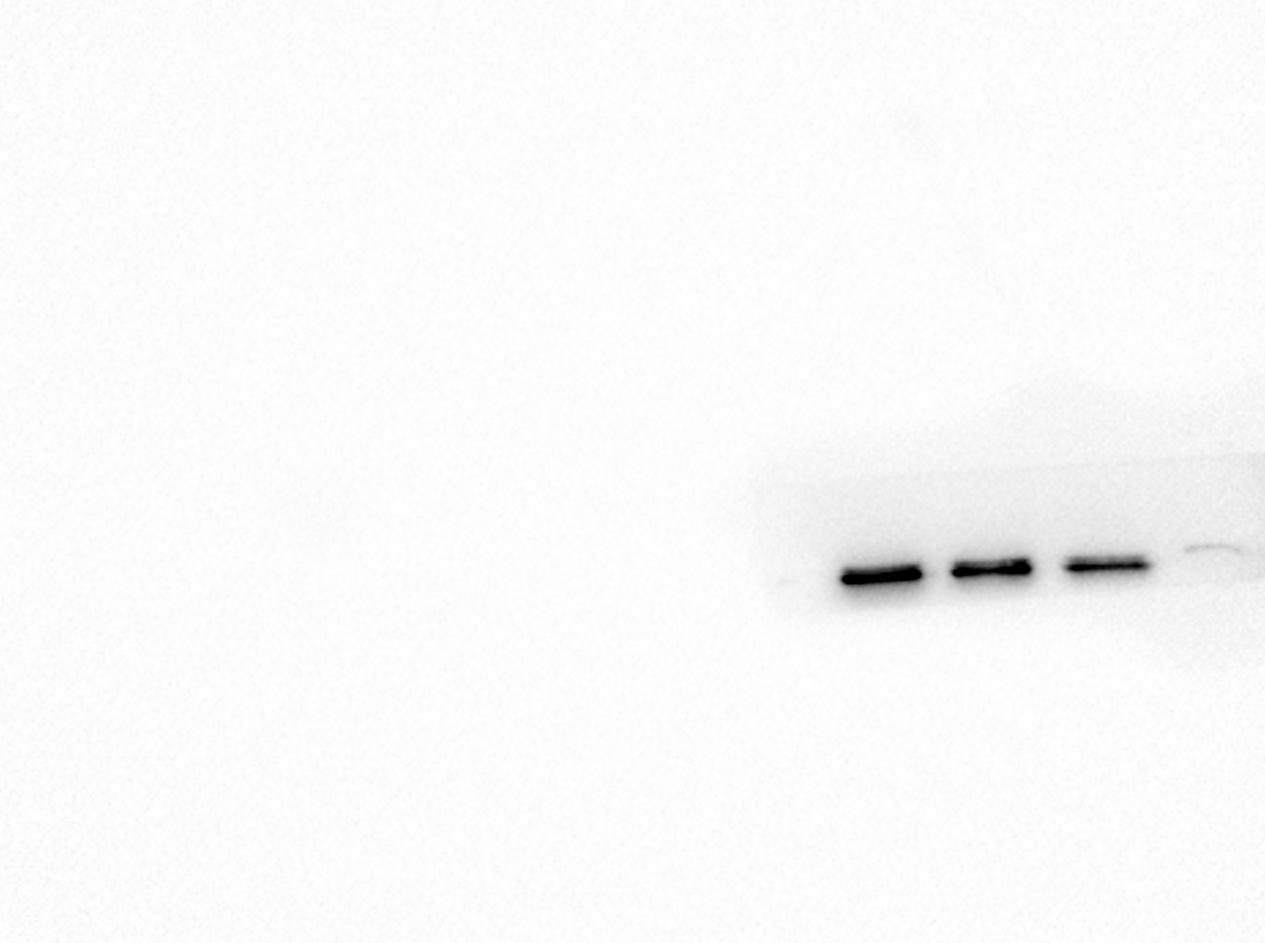


**ERK1/2**


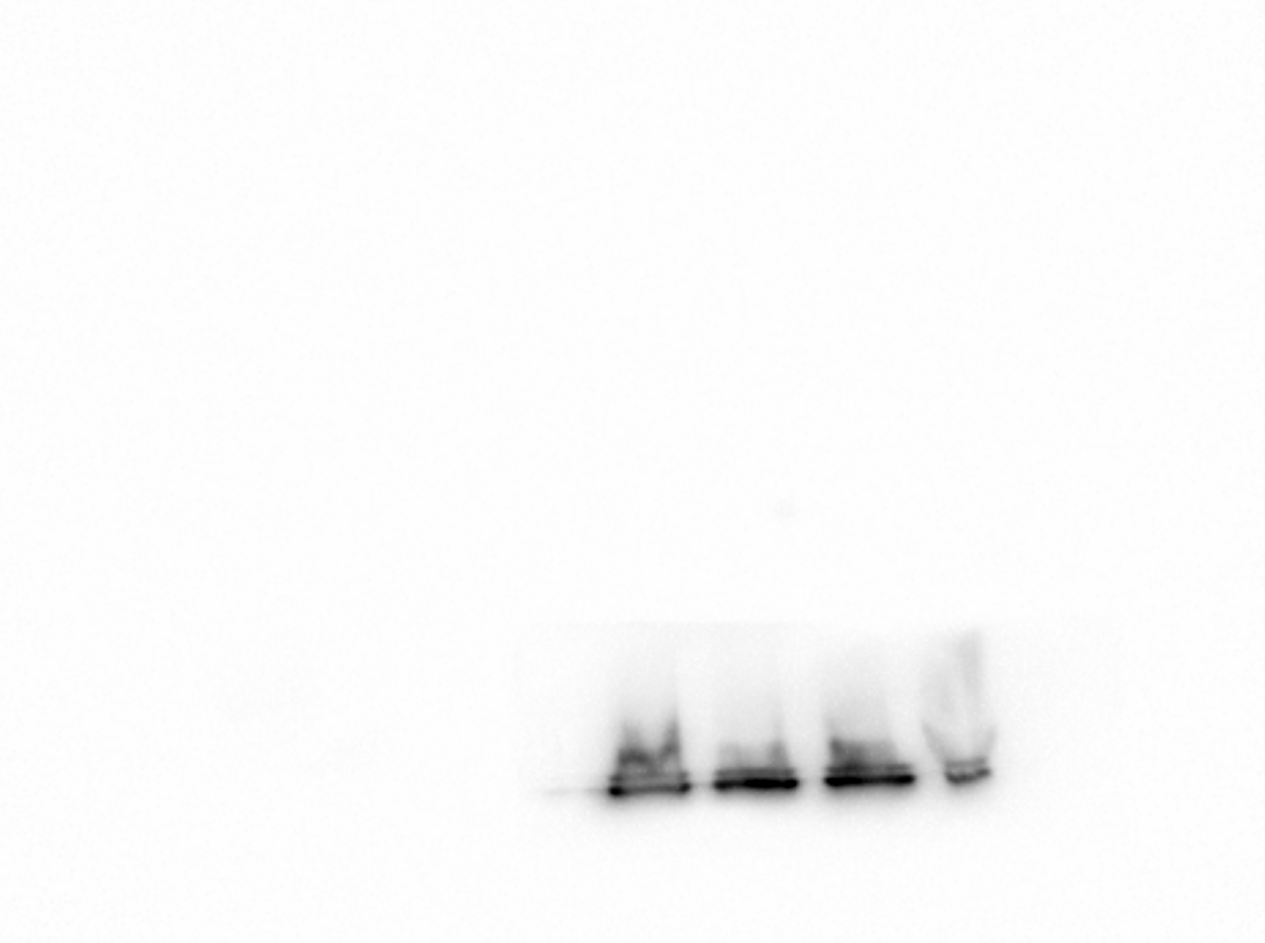


**BAX**


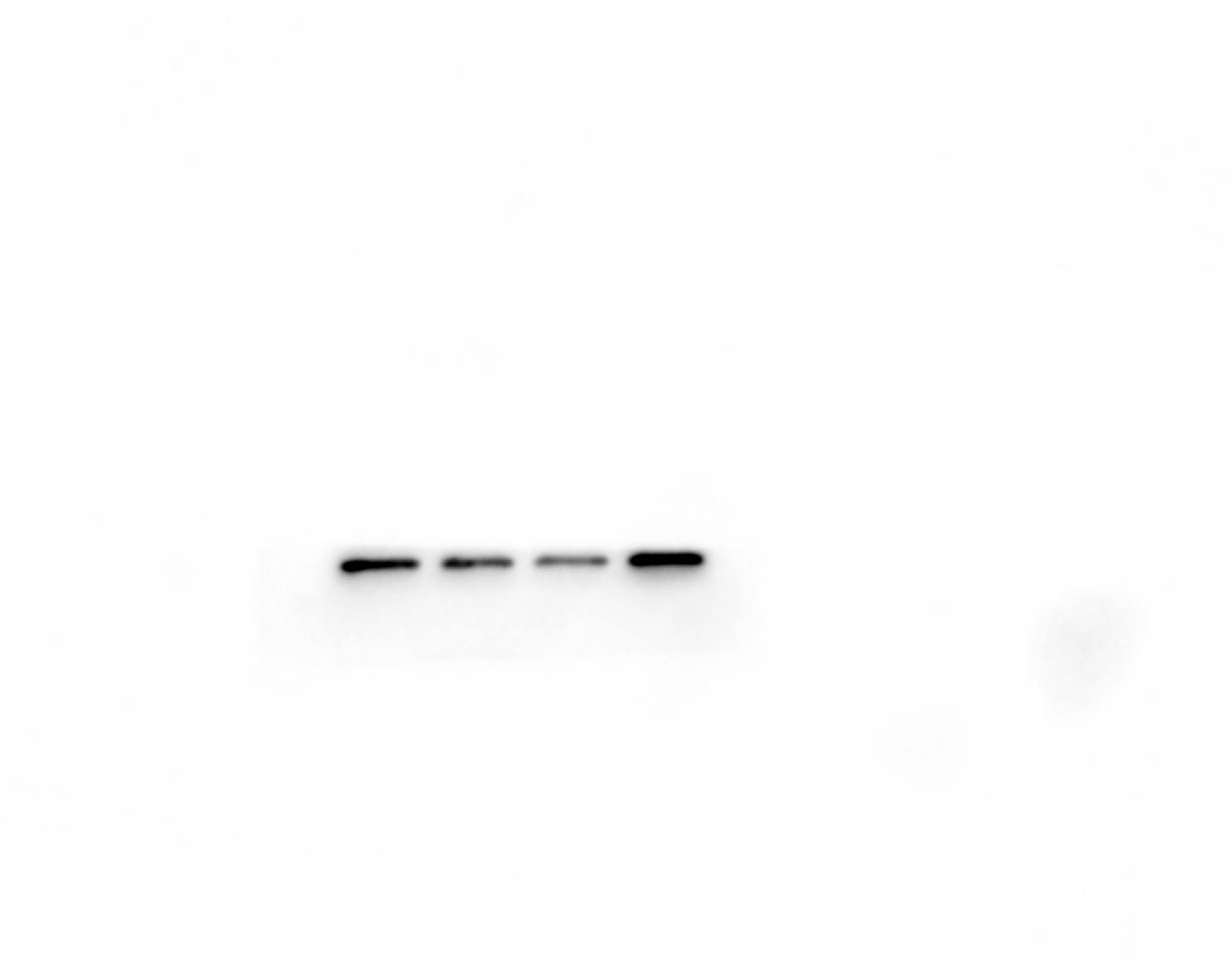


**BCL-2**


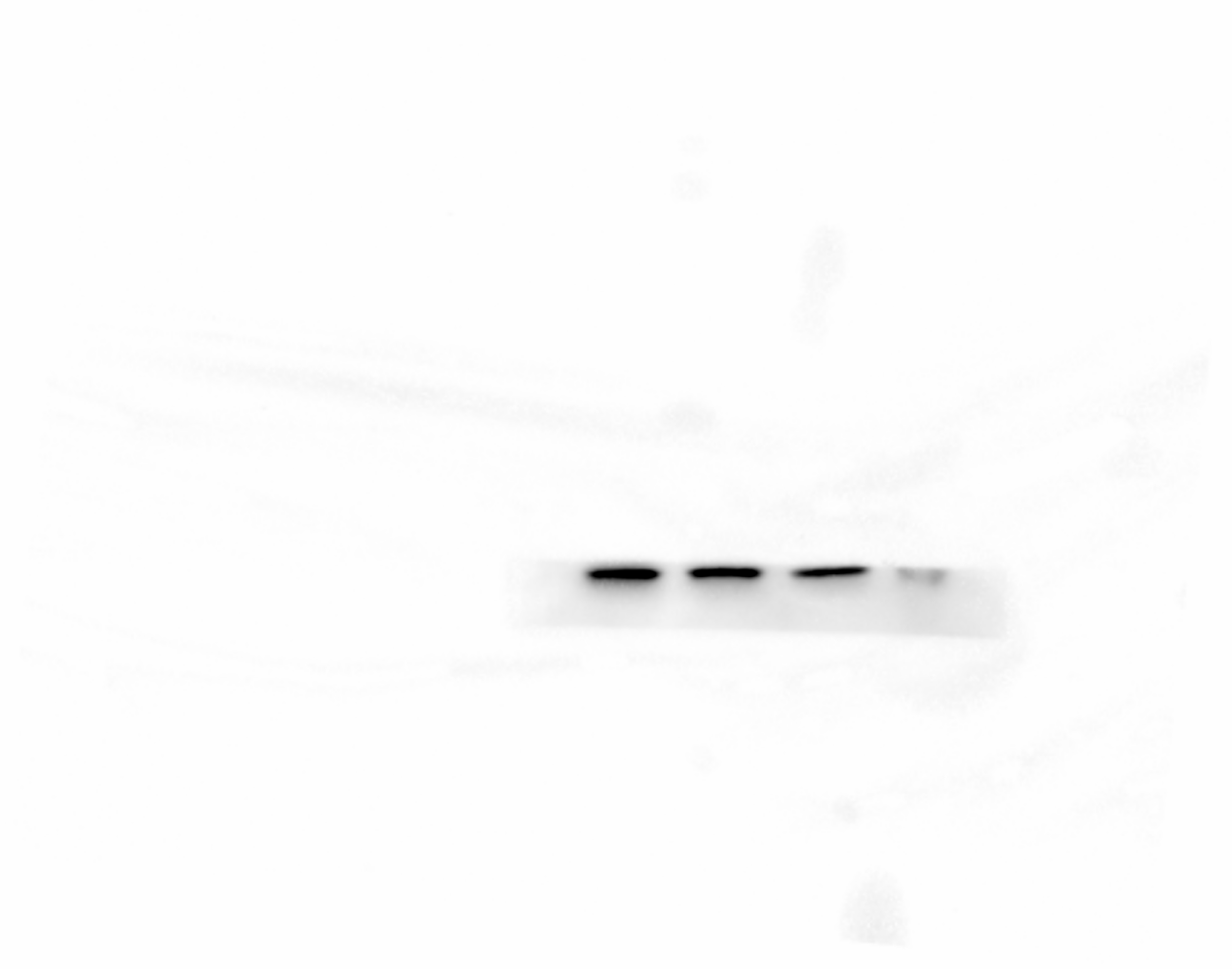


**MCL-1**


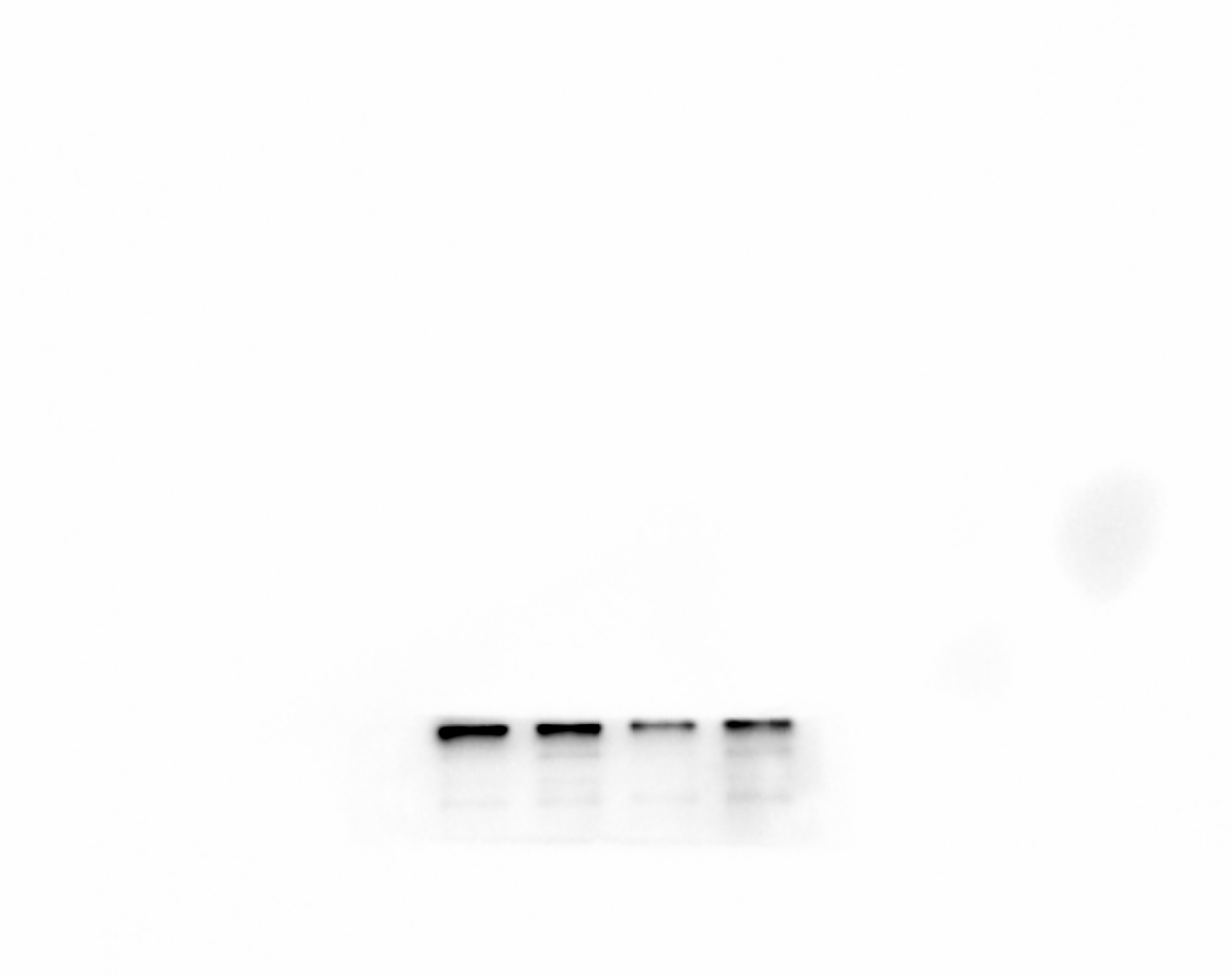


**beta-actin**


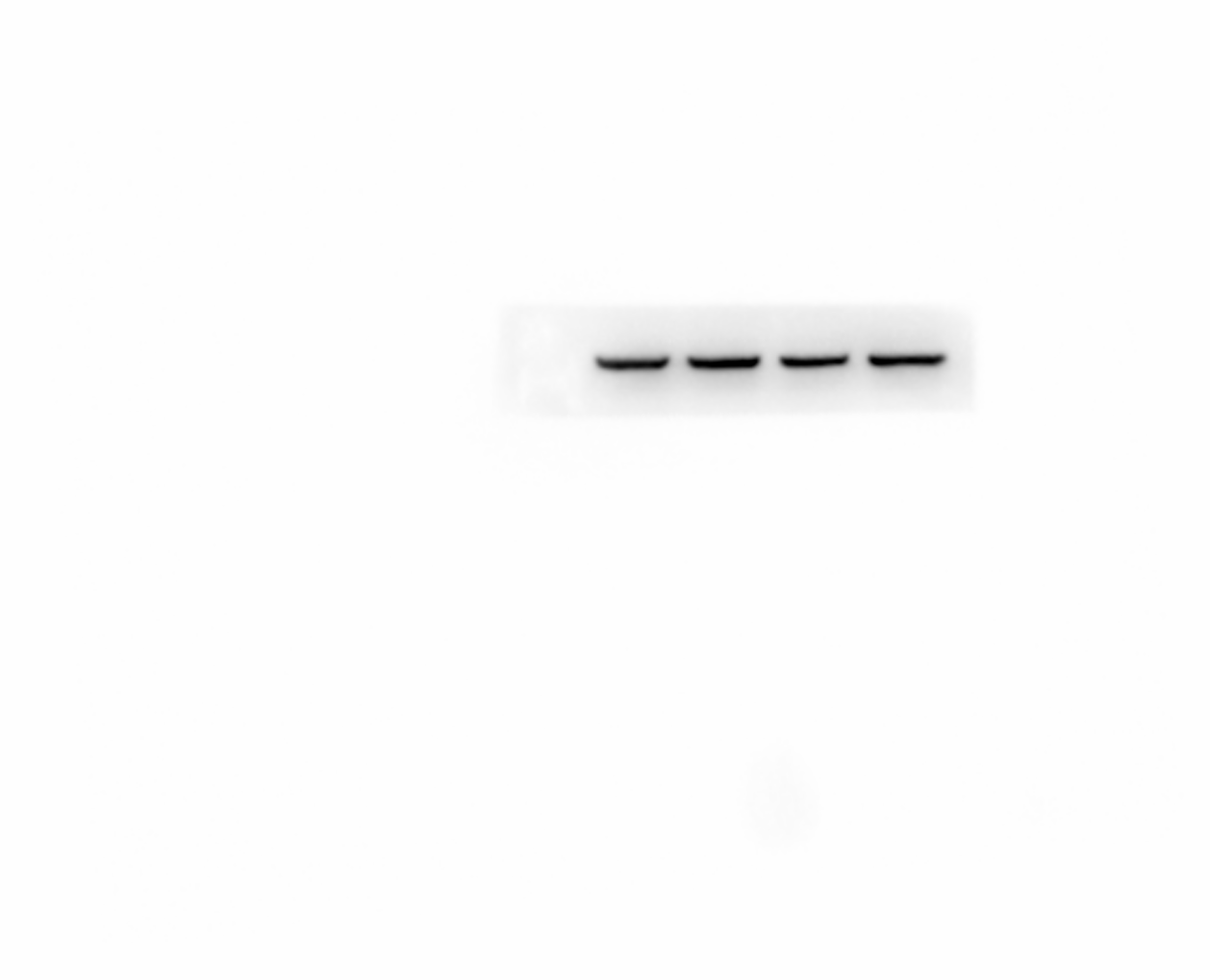


**IκB-alpha**

***
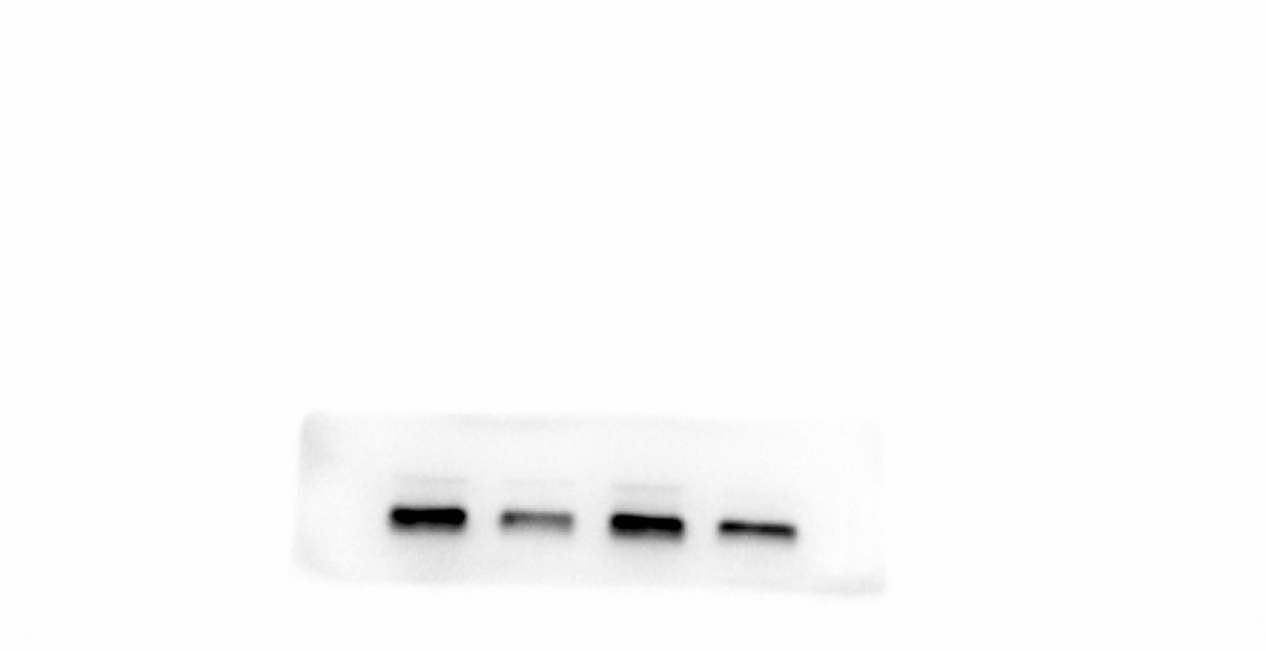
***
