# Supplementary material for: Ginsenoside panaxatriol reverses TNBC paclitaxel resistance by inhibiting the IRAK1/NF-κB and ERK pathways
Source: PeerJ. 2020 Jun 3;8:e9281. doi: 10.7717/peerj.9281 (PMC7275687; doi:10.7717/peerj.9281)
Supplement: Supplemental Information 2 [file peerj-08-9281-s002.docx]

| ***Table S1.* Raw data for qPCR CT value 24h** | | | | | | | | | | | | | | |
| --- | --- | --- | --- | --- | --- | --- | --- | --- | --- | --- | --- | --- | --- | --- |
|  | **18S** | | **IL6** | | **IL8** | | **CXCL1** | | **CCL2** | | **S100A7** | | **S100A9** | |
| **DMSO** | 14.654586 | 14.628469 | 30.81347847 | 30.704525 | 22.125849 | 22.118959 | 27.508564 | 27.496943 | 29.519962 | 29.497108 | 28.879343 | 28.915785 | 32.227928 | 32.719506 |
| **PTX** | 14.825779 | 14.73597 | 28.84378198 | 28.915484 | 21.489687 | 21.418742 | 27.127517 | 27.021599 | 27.19961 | 27.167506 | 27.74523 | 27.594221 | 30.538586 | 31.185461 |
| **PT** | 13.21214 | 13.183847 | 29.40676985 | 29.383998 | 20.973173 | 21.005358 | 26.387705 | 26.381942 | 27.937483 | 27.97464 | 27.356664 | 27.479519 | 30.893579 | 31.314226 |
| **PTX+PT** | 13.847047 | 13.547615 | 29.13769319 | 28.833937 | 21.125577 | 21.20073 | 26.551353 | 26.402285 | 27.55011 | 27.693354 | 27.082628 | 27.20428 | 30.792959 | 31.410381 |

***Table S2.* Raw data for qPCR CT value 24h (continued)**

|  | **18S** | | **OCT4** | | **SOX2** | | **NANOG** | | **CD44** | | **ALDH1** | |
| --- | --- | --- | --- | --- | --- | --- | --- | --- | --- | --- | --- | --- |
| **DMSO** | 11.478293 | 11.496413 | 30.92447662 | 31.186642 | 32.997459 | 31.411179 | 28.636639 | 28.905518 | 18.108501 | 18.021978 | 32.002838 | 32.911304 |
| **PTX** | 12.042658 | 11.953481 | 31.21488108 | 31.444451 | 32.623871 | 30.873721 | 28.856614 | 28.904757 | 18.521183 | 18.444186 | 31.848584 | 32.948802 |
| **PT** | 11.845497 | 11.781176 | 31.38740137 | 31.60443 | 33.557215 | 31.900313 | 29.030269 | 29.213998 | 18.861085 | 18.667077 | 32.692762 | 33.543871 |
| **PTX+PT** | 11.772391 | 11.674336 | 31.50035262 | 31.937217 | 33.55365 | 32.846209 | 29.088661 | 29.574106 | 18.736422 | 18.487652 | 33.451025 | 33.714172 |

***Table S3.* Raw data for qPCR CT value 48h**

|  | **18S** | | **IL6** | | **IL8** | | **CXCL1** | | **CCL2** | | **S100A7** | | **S100A9** | |
| --- | --- | --- | --- | --- | --- | --- | --- | --- | --- | --- | --- | --- | --- | --- |
| **DMSO** | 12.512431 | 12.391443 | 30.80656433 | 30.628981 | 22.564619 | 22.580149 | 27.413393 | 27.253183 | 32.128719 | 31.745401 | 28.46838 | 28.614761 | 32.158161 | 33.482716 |
| **PTX** | 13.234319 | 13.147666 | 27.9885783 | 27.922939 | 22.453688 | 22.525172 | 26.562528 | 26.49637 | 29.848837 | 29.537678 | 27.698709 | 27.996535 | 30.745441 | 31.827699 |
| **PT** | 12.663682 | 12.70055 | 30.96373189 | 30.850017 | 22.709226 | 22.783853 | 27.583459 | 27.431965 | 32.234471 | 31.69306 | 28.5922 | 29.099317 | 32.470355 | 33.673796 |
| **PTX+PT** | 12.791941 | 12.757323 | 29.59299281 | 29.362566 | 22.259892 | 22.298026 | 26.605608 | 26.615114 | 30.01759 | 29.631371 | 28.94089 | 28.657392 | 32.562871 | 33.710697 |

***Table S4.* Raw data for qPCR CT value 48h (continued)**

|  | **18S** | | **OCT4** | | **NANOG** | | **CD44** | |
| --- | --- | --- | --- | --- | --- | --- | --- | --- |
| **DMSO** | 13.732825 | 13.805447 | 34.42969131 | 34.47076 | 31.329983 | 31.839161 | 19.940117 | 19.777689 |
| **PTX** | 14.135152 | 14.125635 | 34.44587763 | 34.544365 | 31.016856 | 31.512679 | 19.93629 | 19.782841 |
| **PT** | 13.541458 | 13.48876 | 34.4909121 | 34.593127 | 31.074959 | 31.685193 | 20.251669 | 20.210874 |
| **PTX+PT** | 13.609197 | 13.556749 | 35.18195952 | 35.459004 | 31.583999 | 31.854695 | 20.593121 | 20.555539 |

***Table S5.* Raw data for qPCR CT value 48h (continued)**

|  | **18S** | | **SOX2** | | **ALDH1** | |
| --- | --- | --- | --- | --- | --- | --- |
| **DMSO** | 11.597721 | 11.663932 | 32.31998444 | 31.388847 | 32.667679 | 33.645672 |
| **PTX** | 11.91592 | 11.945827 | 31.38898842 | 30.64692 | 32.009847 | 33.136025 |
| **PT** | 11.931091 | 11.850897 | 32.63389309 | 31.861962 | 33.374525 | 34.0902 |
| **PTX+PT** | 11.832992 | 11.763094 | 32.87625127 | 32.149396 | 34.342921 | 34.797345 |

***Fig. S8* Raw data for Transwell invasion**

**DMSO**


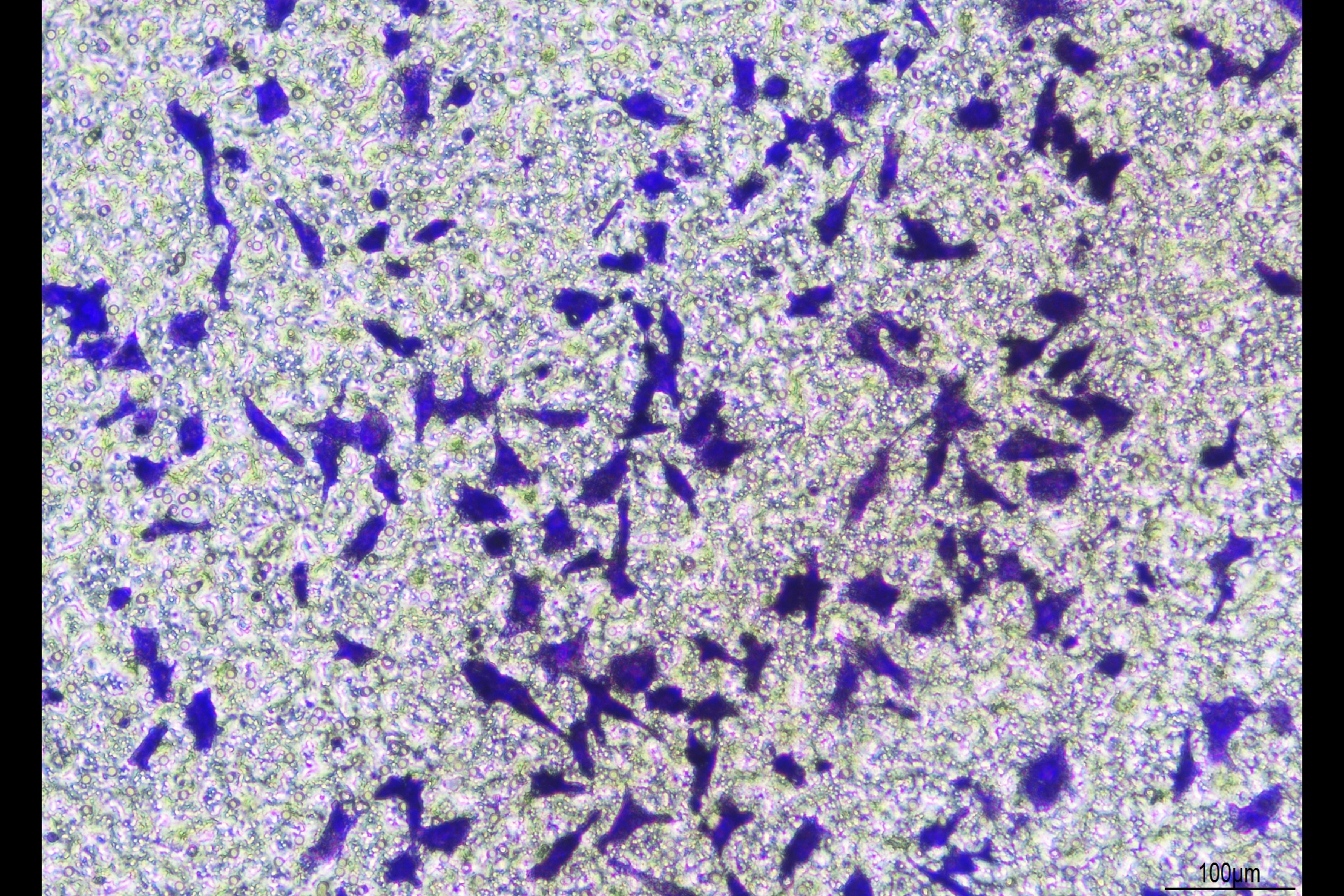


**PTX**


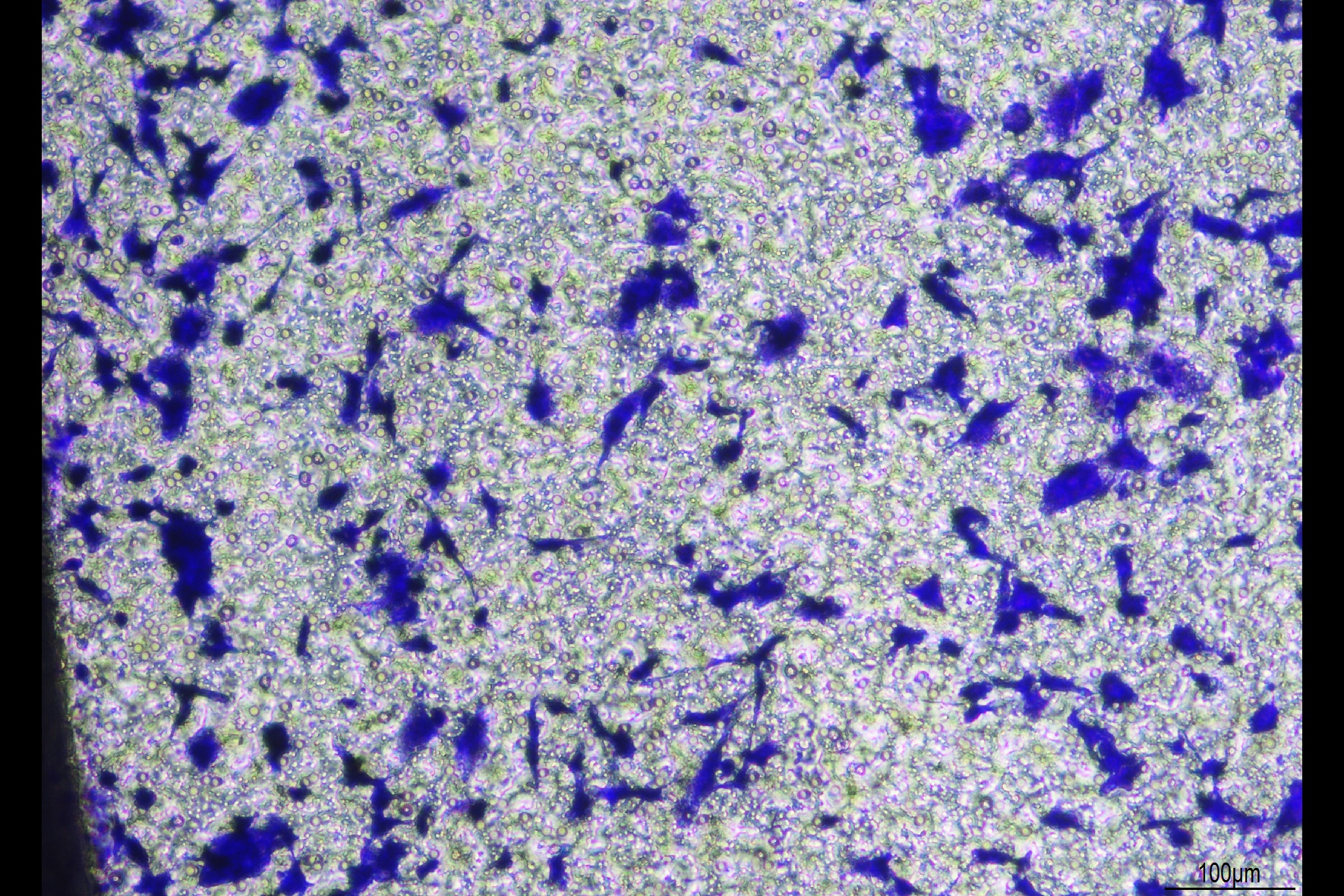


**GPT**


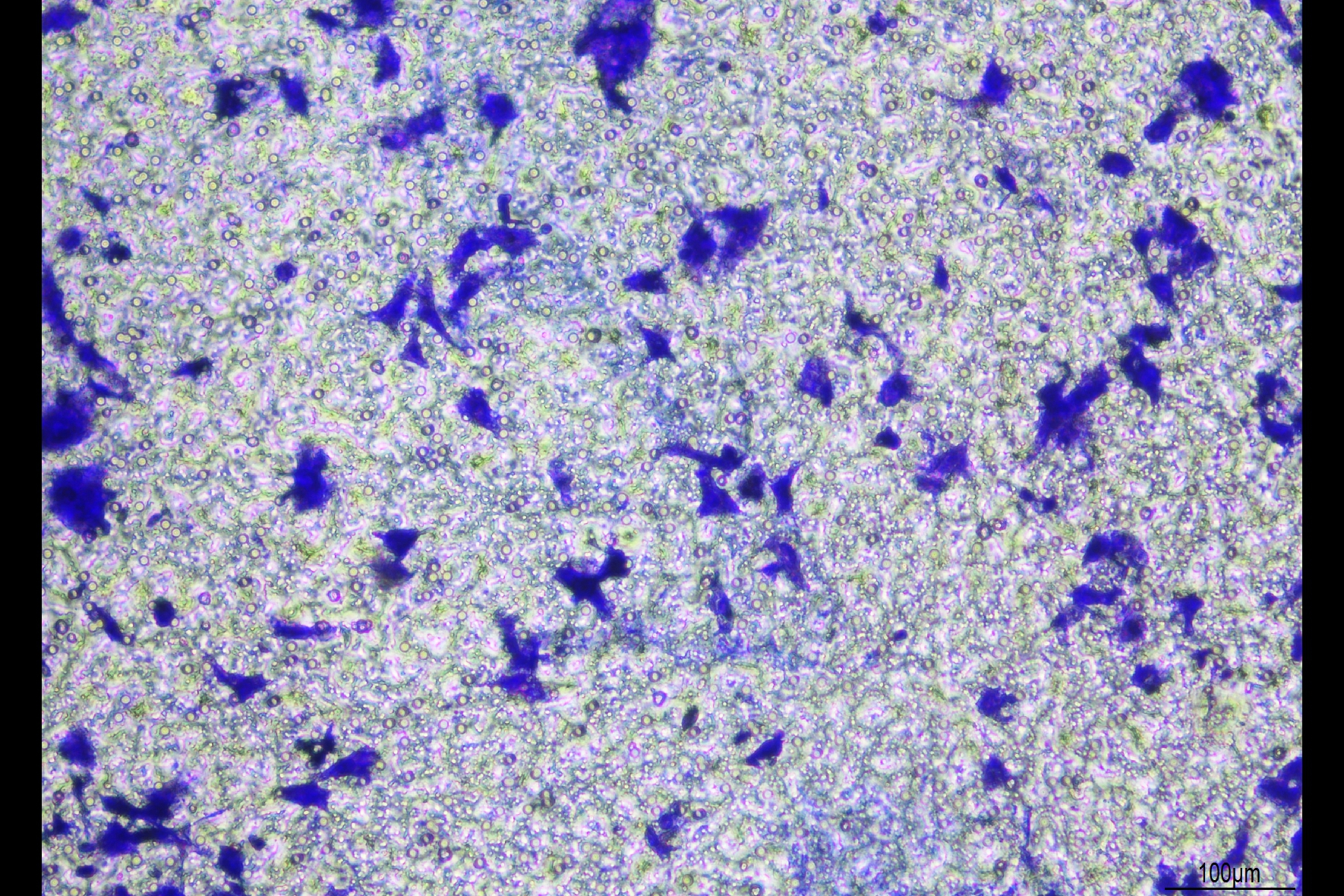


**PTX+PT**


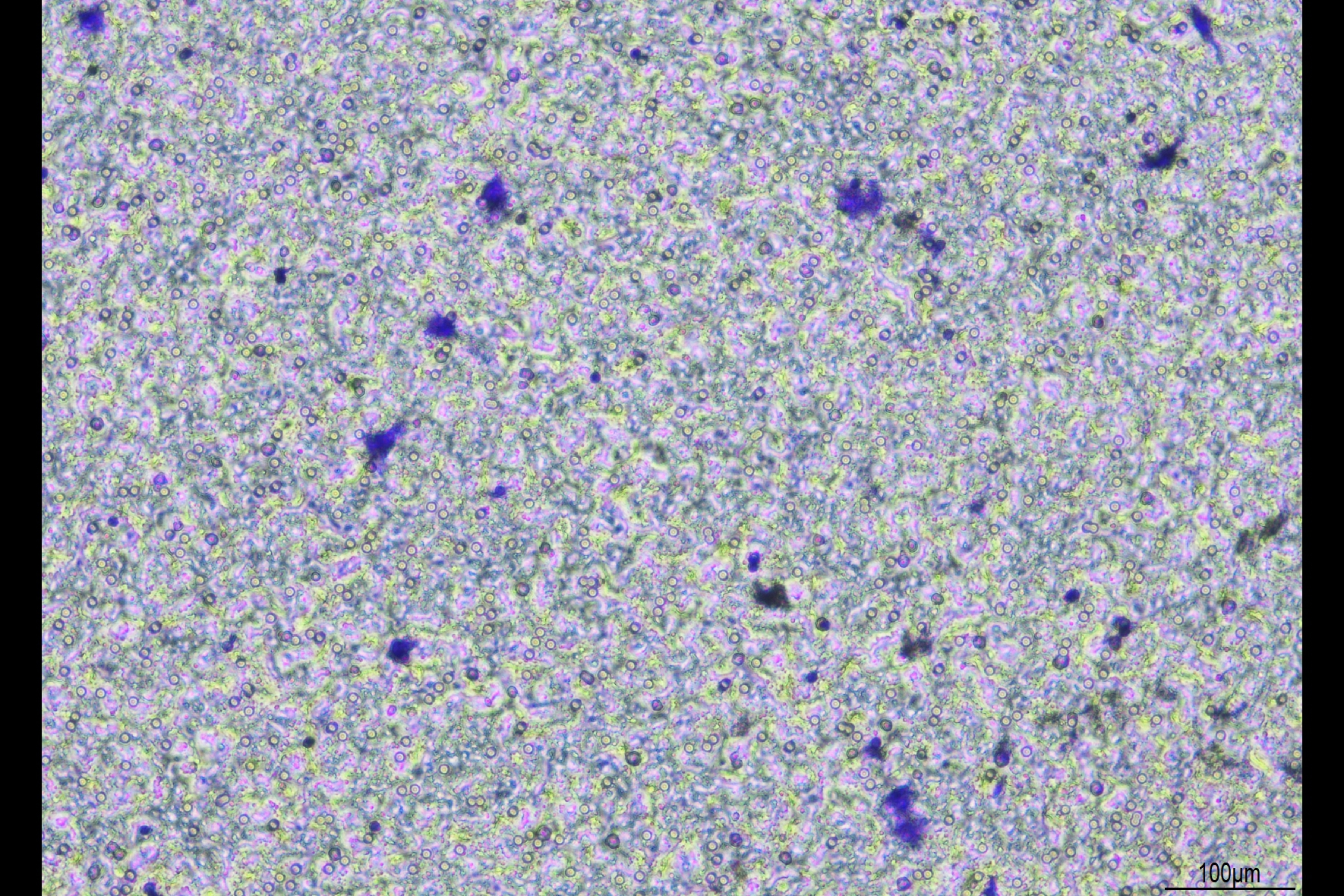


***Fig. S9* Raw data for *MB231-PR* Tumor sphere**

**DMSO DAY 0**

*
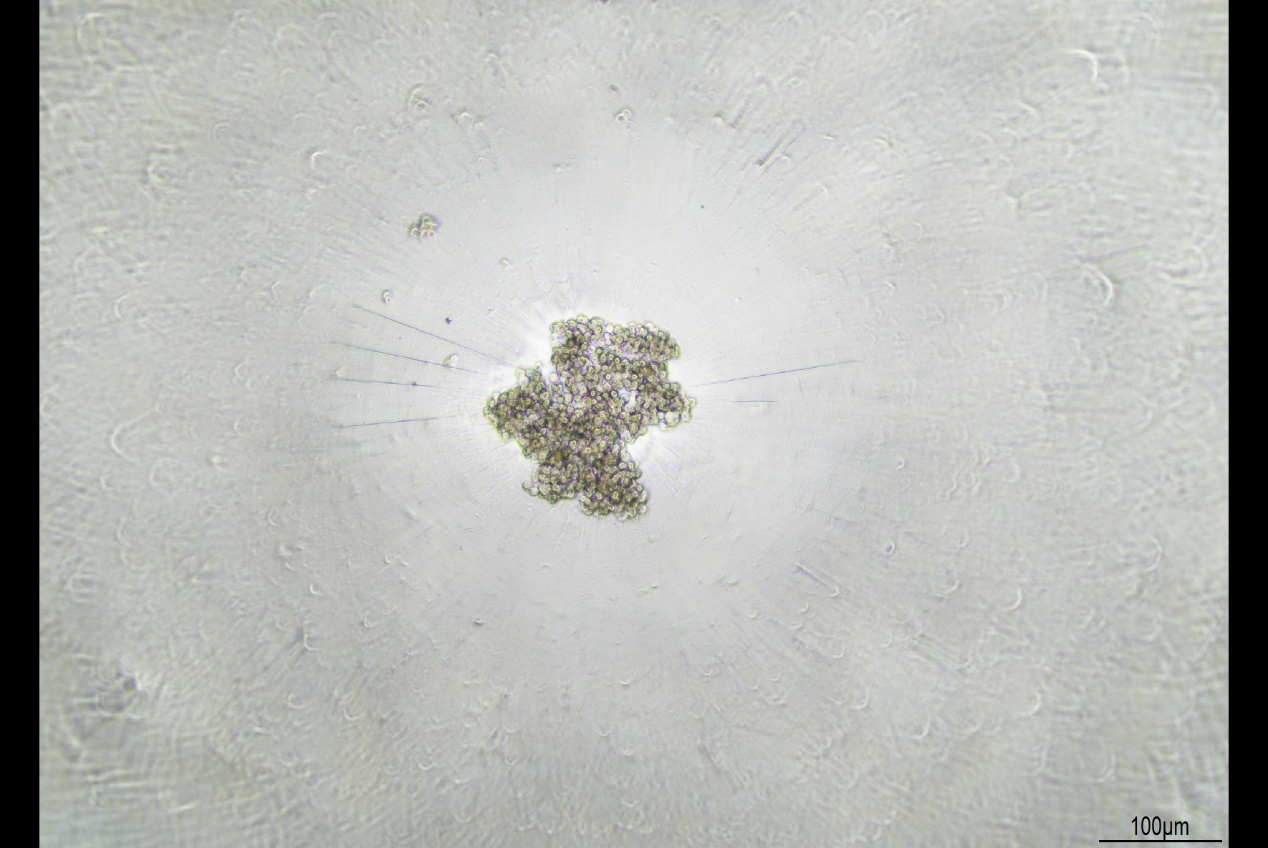
*

**DMSO DAY 6**

*
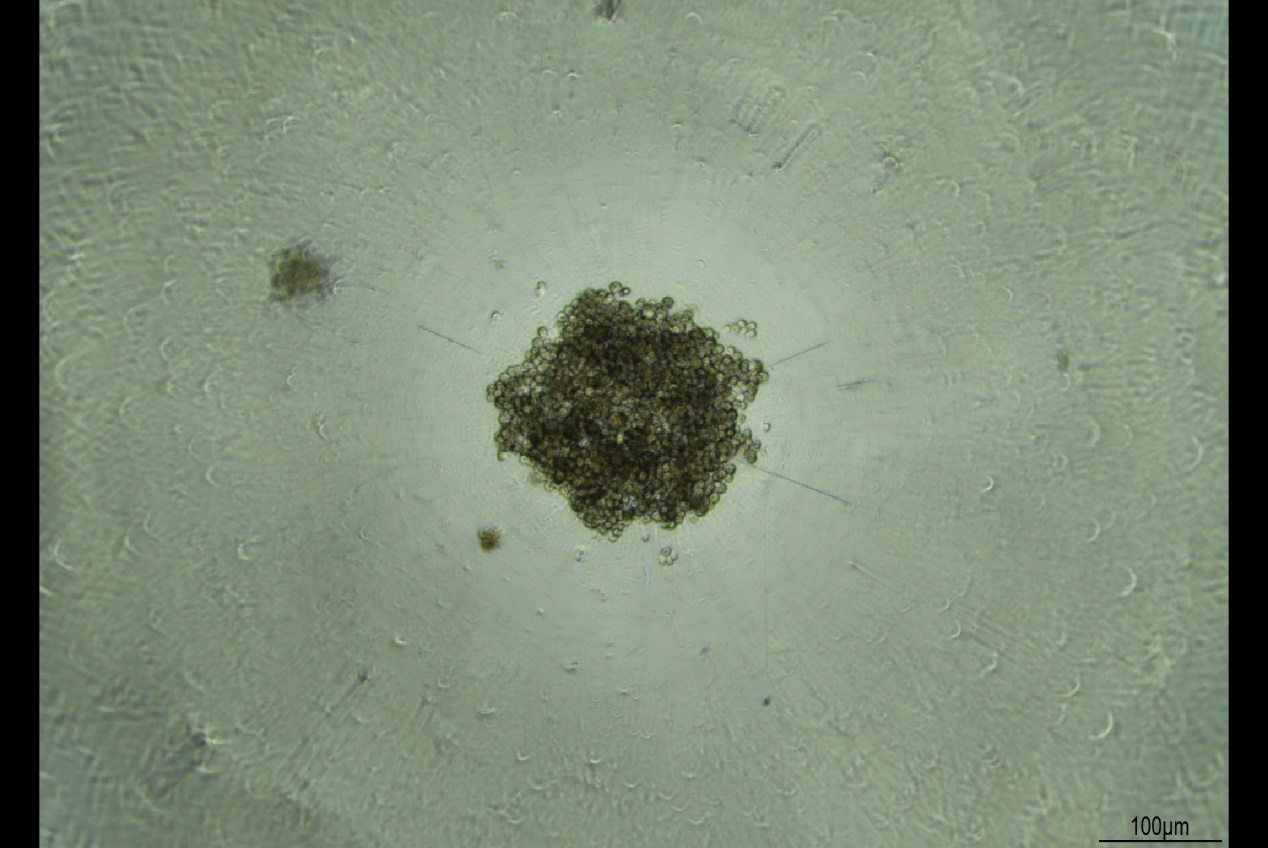
*

**DMSO DAY 12**

*
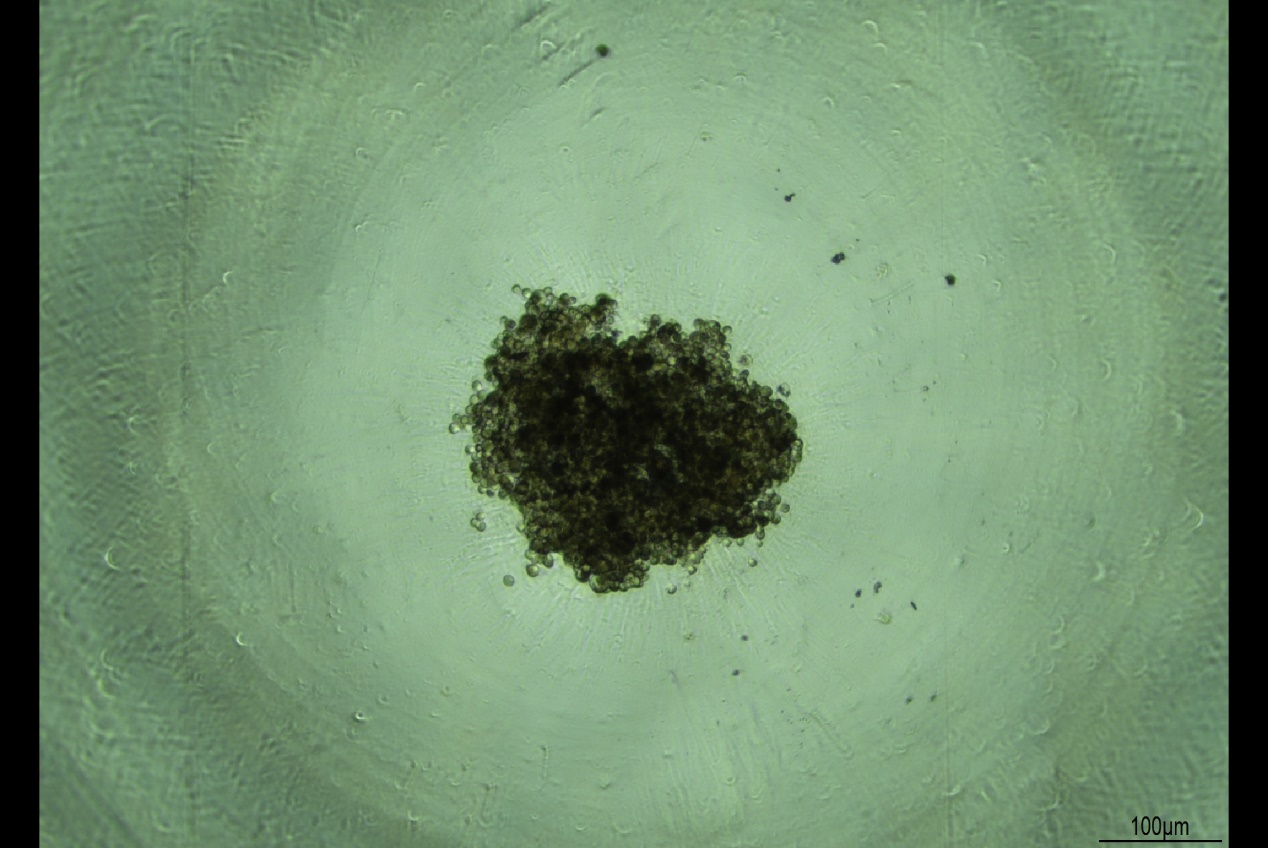
*

**PTX DAY 0**

*
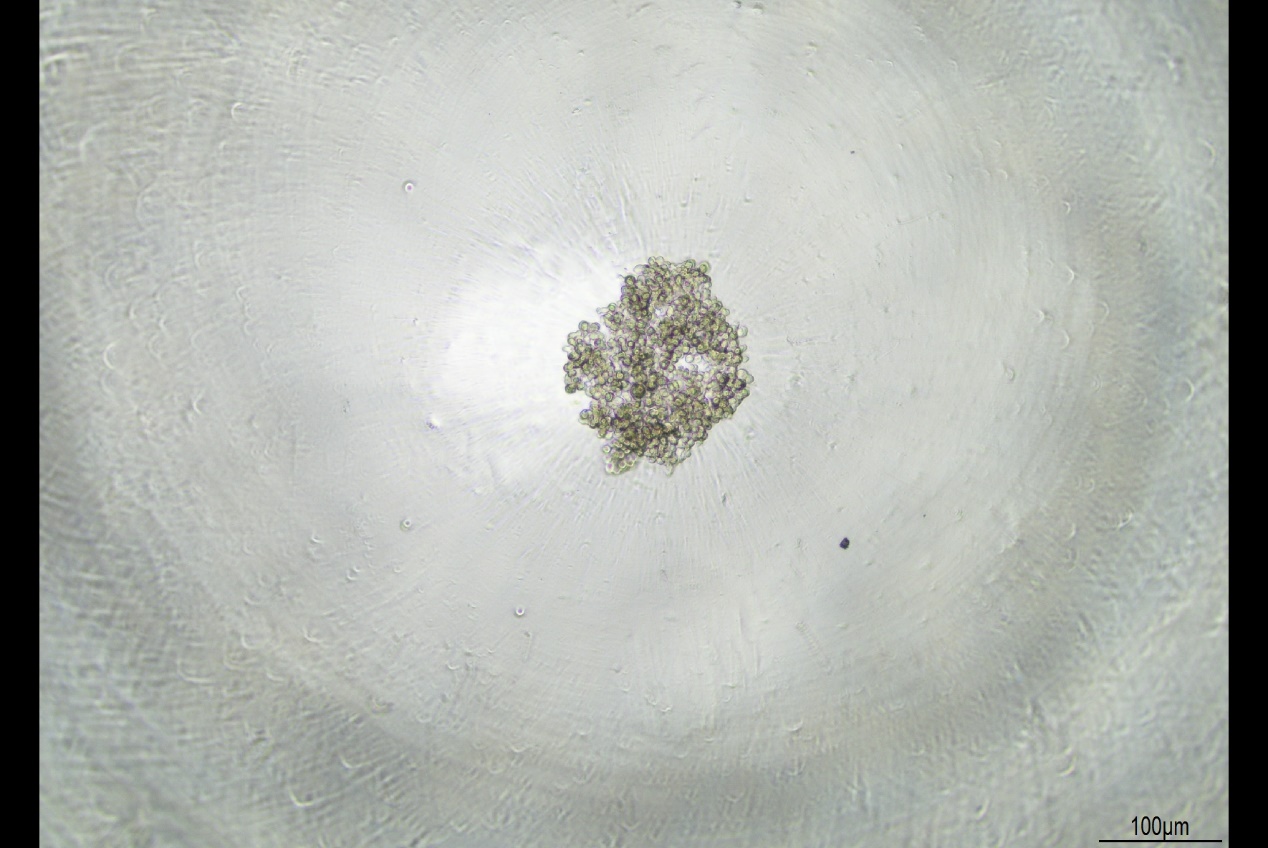
*

**PTX DAY 6**

*
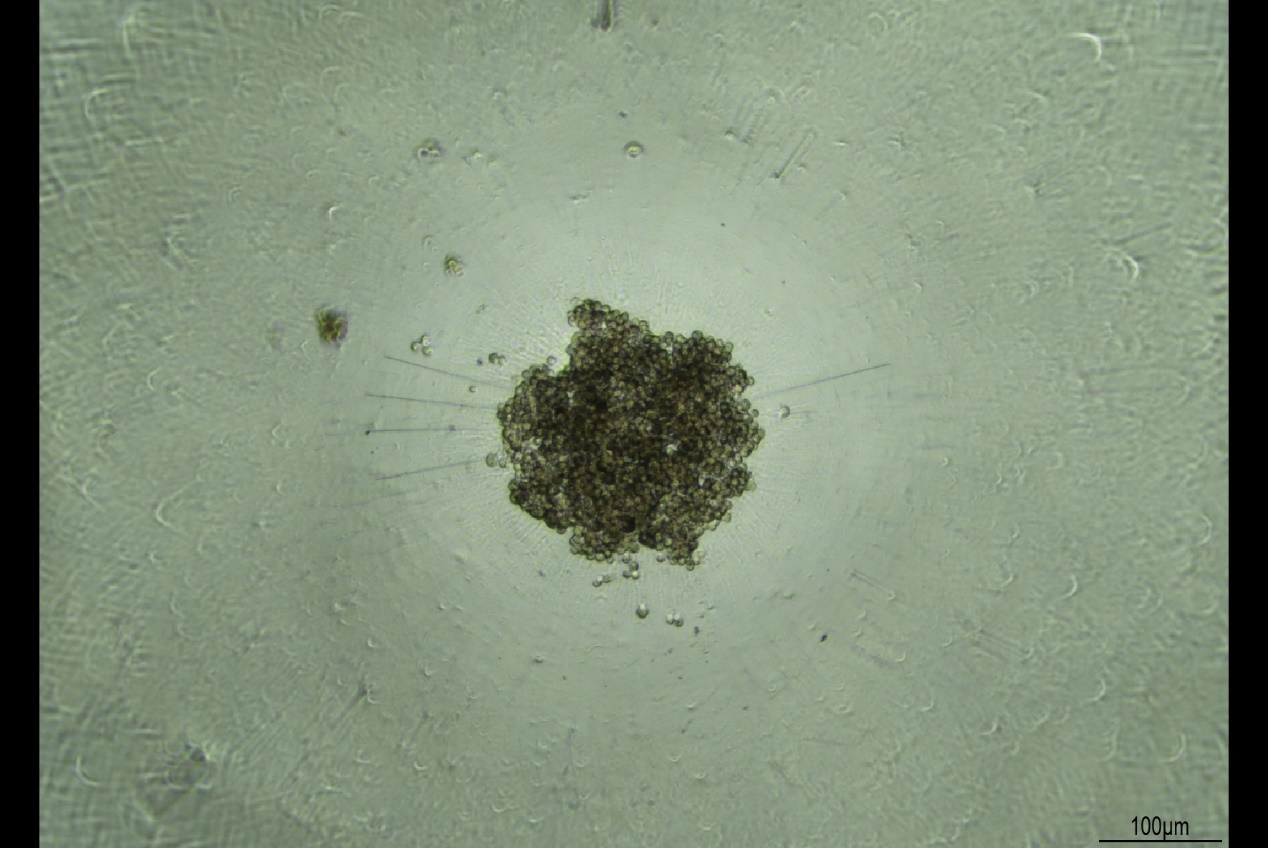
*

**PTX DAY 12**

**
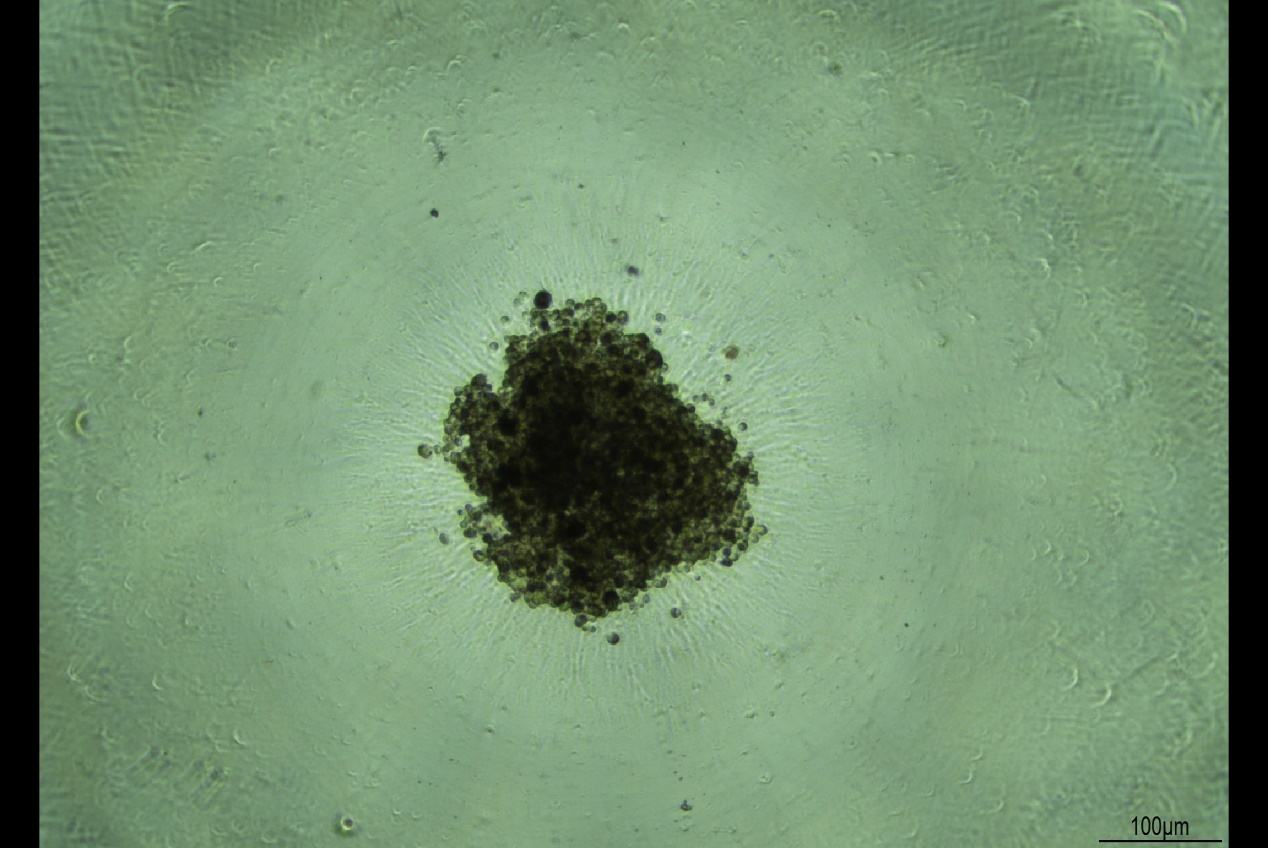
**

**GPT DAY 0**

**
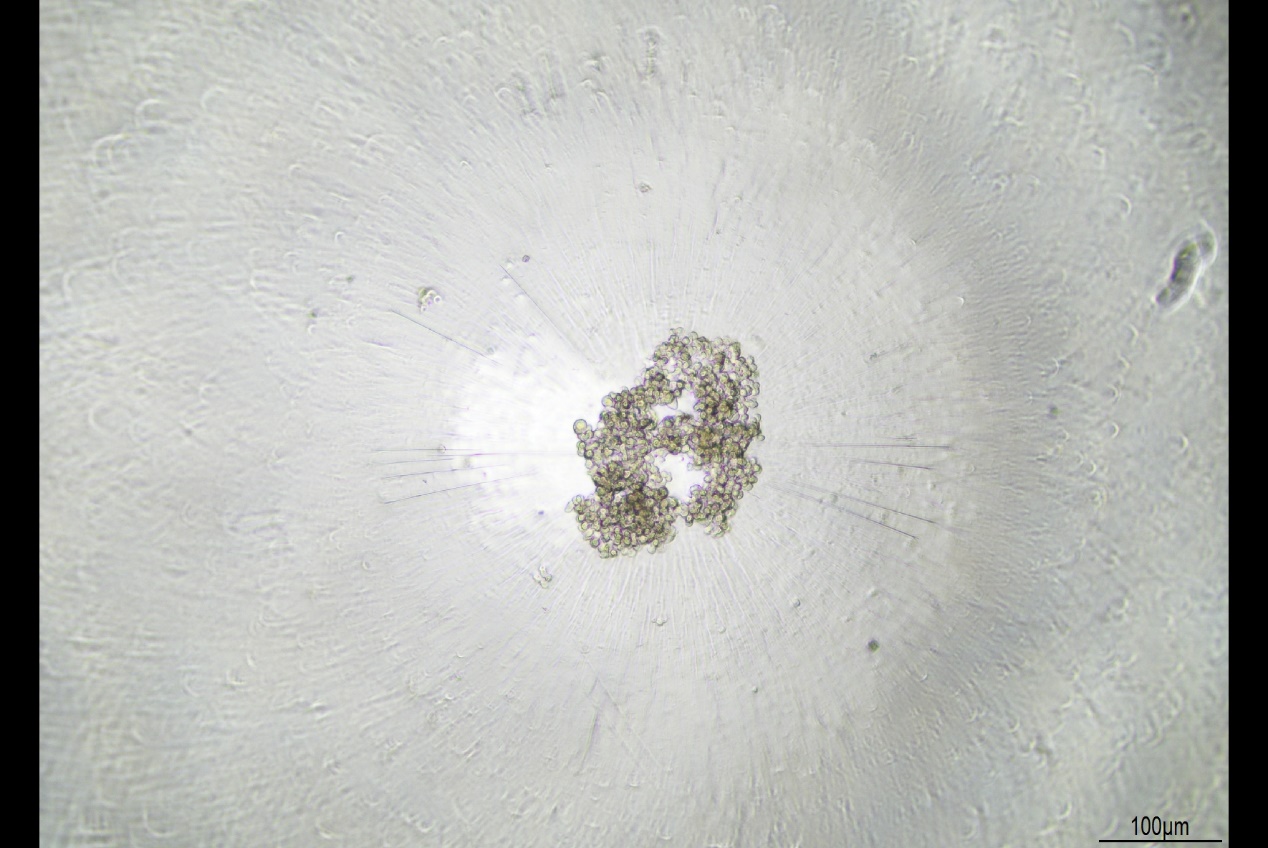
**

**GPT DAY 6**

**
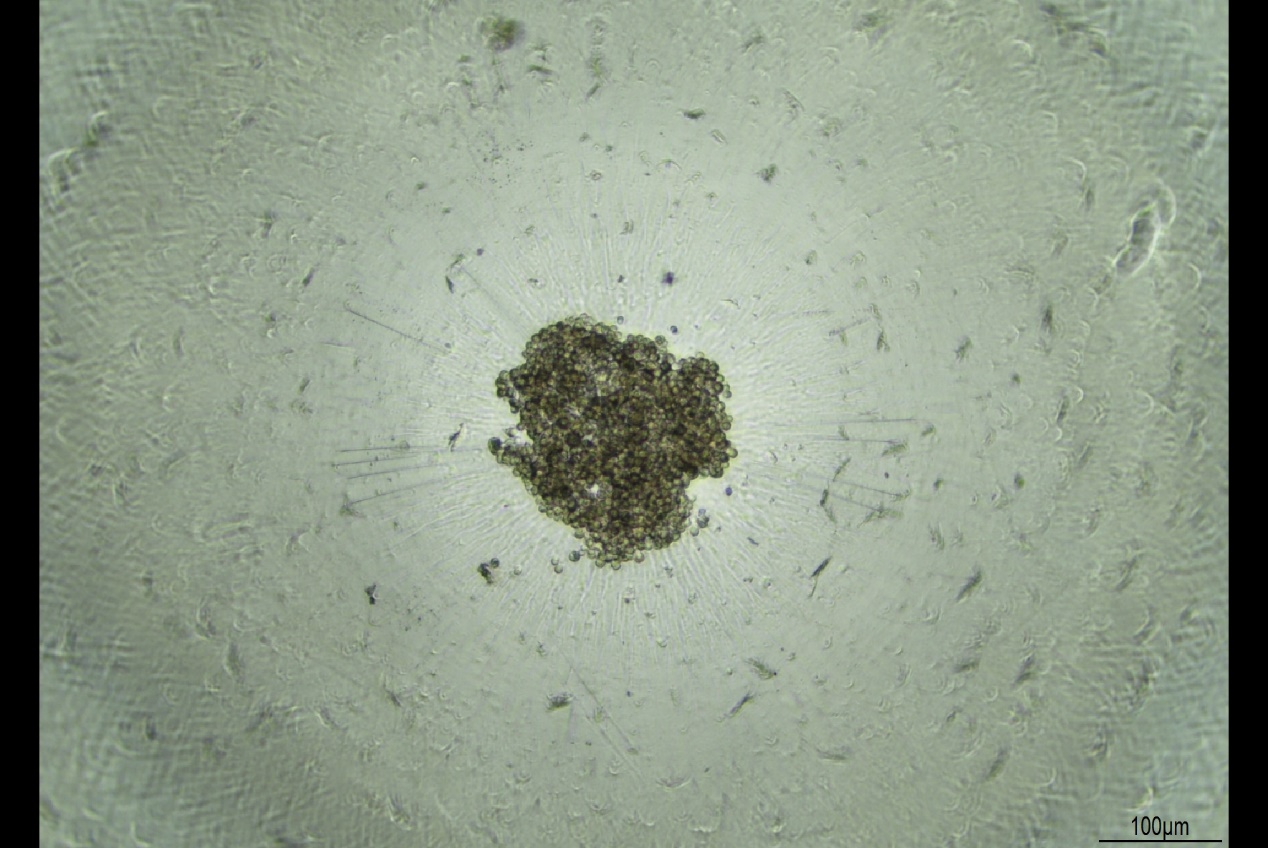
**

**GPT DAY 12**

**
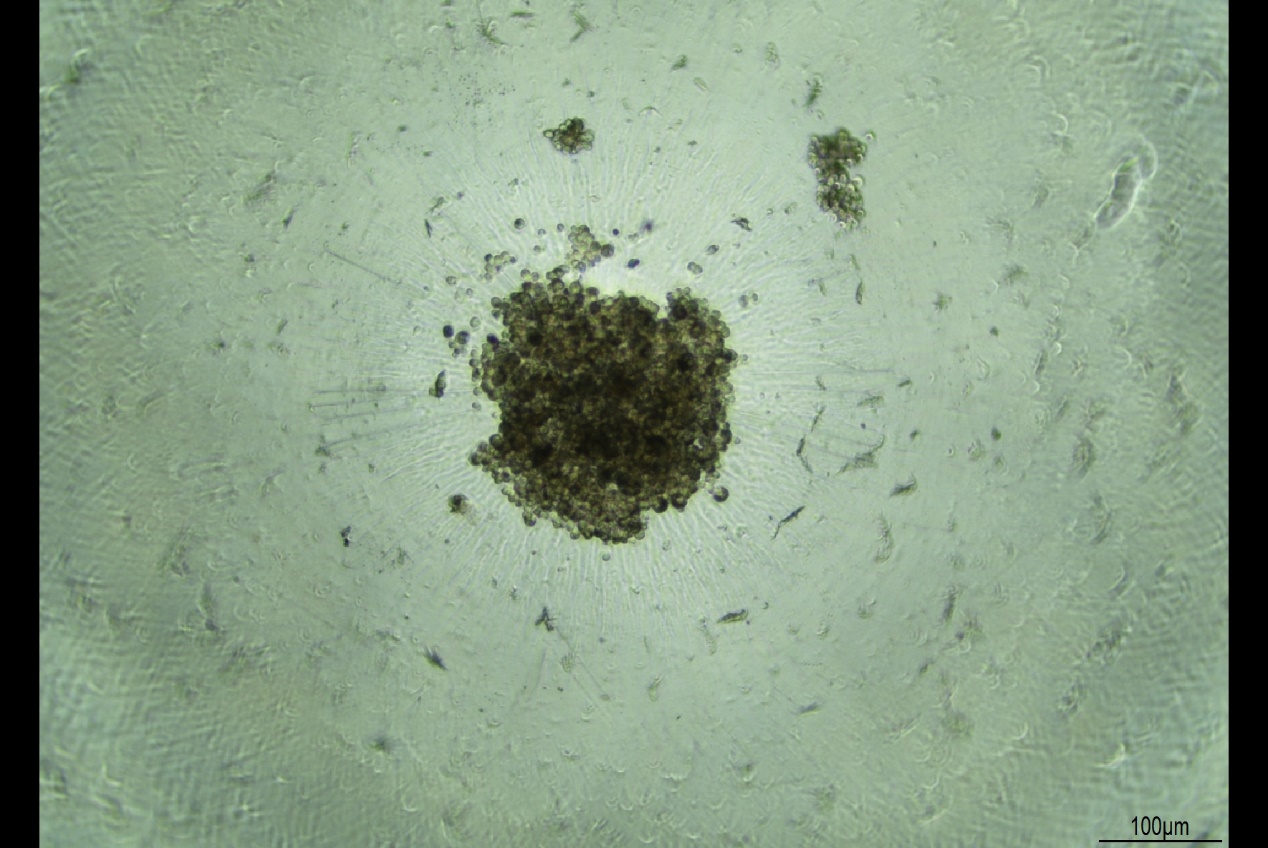
**

**PTX+GPT DAY 0**

**
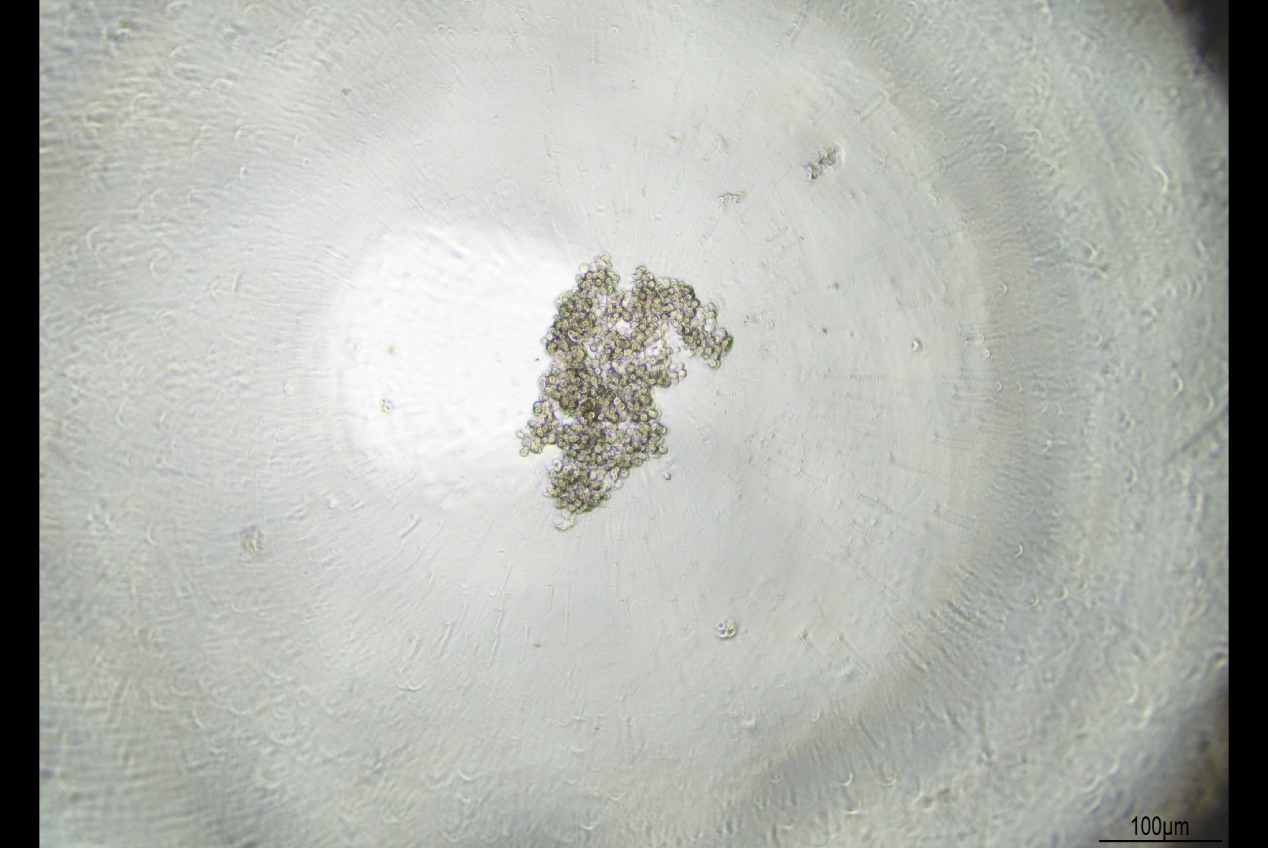
**

**PTX+GPT DAY 6**

**
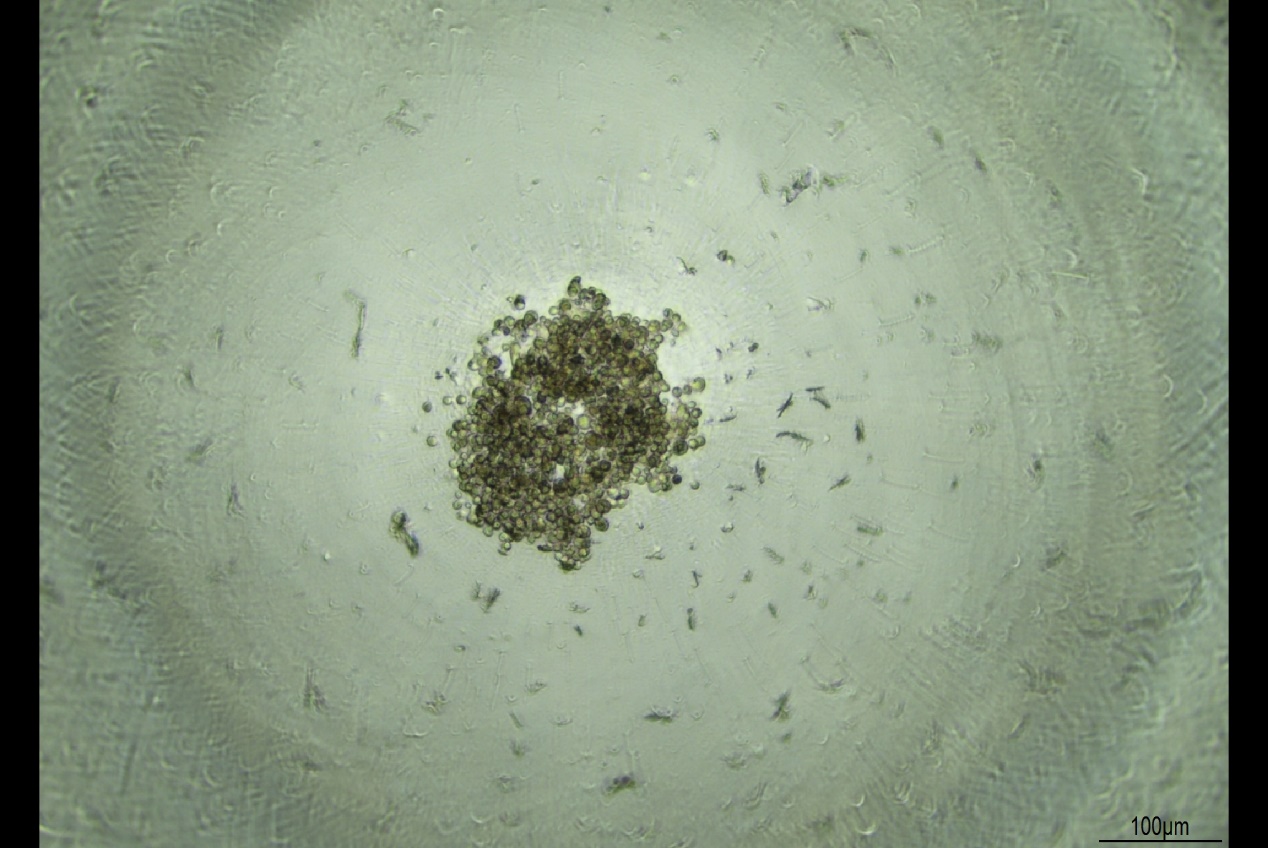
**

**PTX+GPT DAY 12**

*
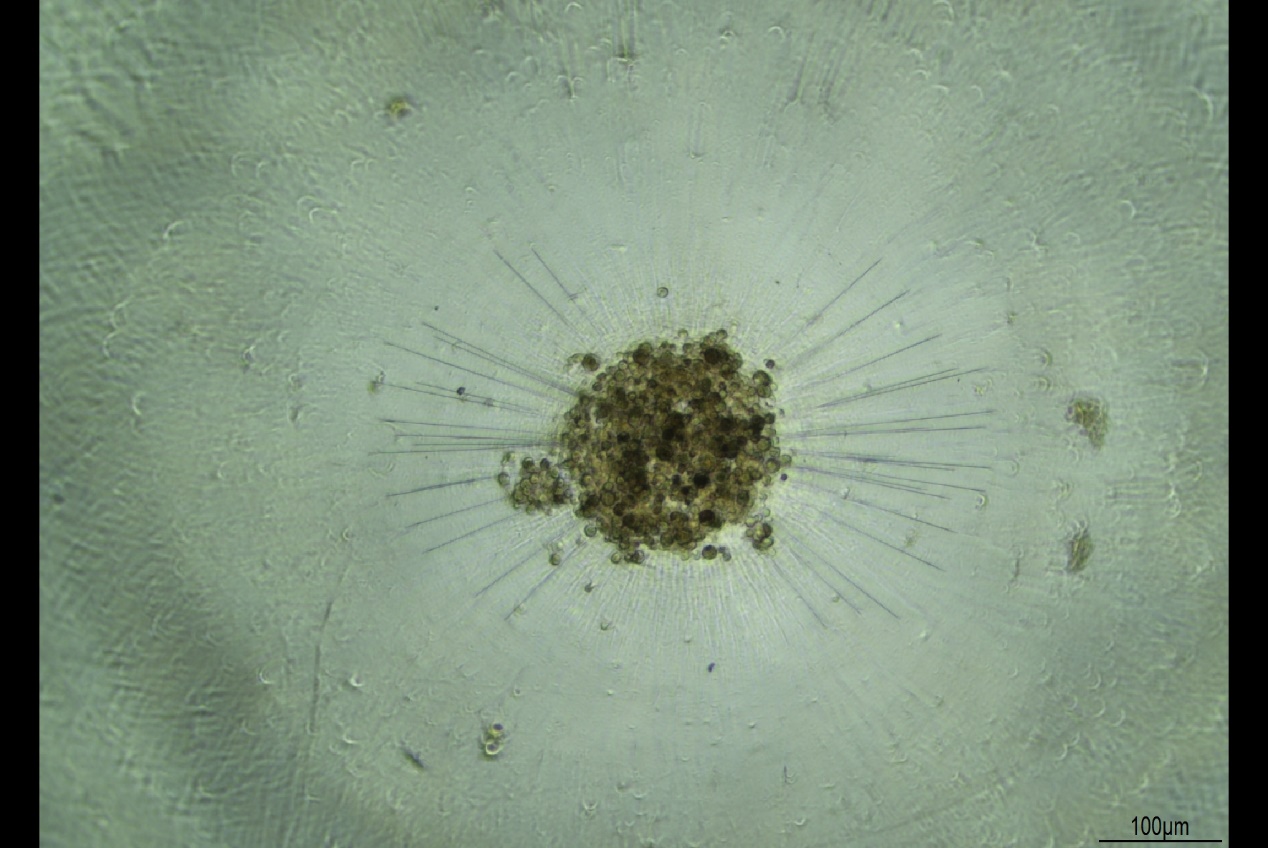
*

***Fig. S10* Raw data for *SUM159-PR* Tumor sphere**

**DMSO DAY 0**

**
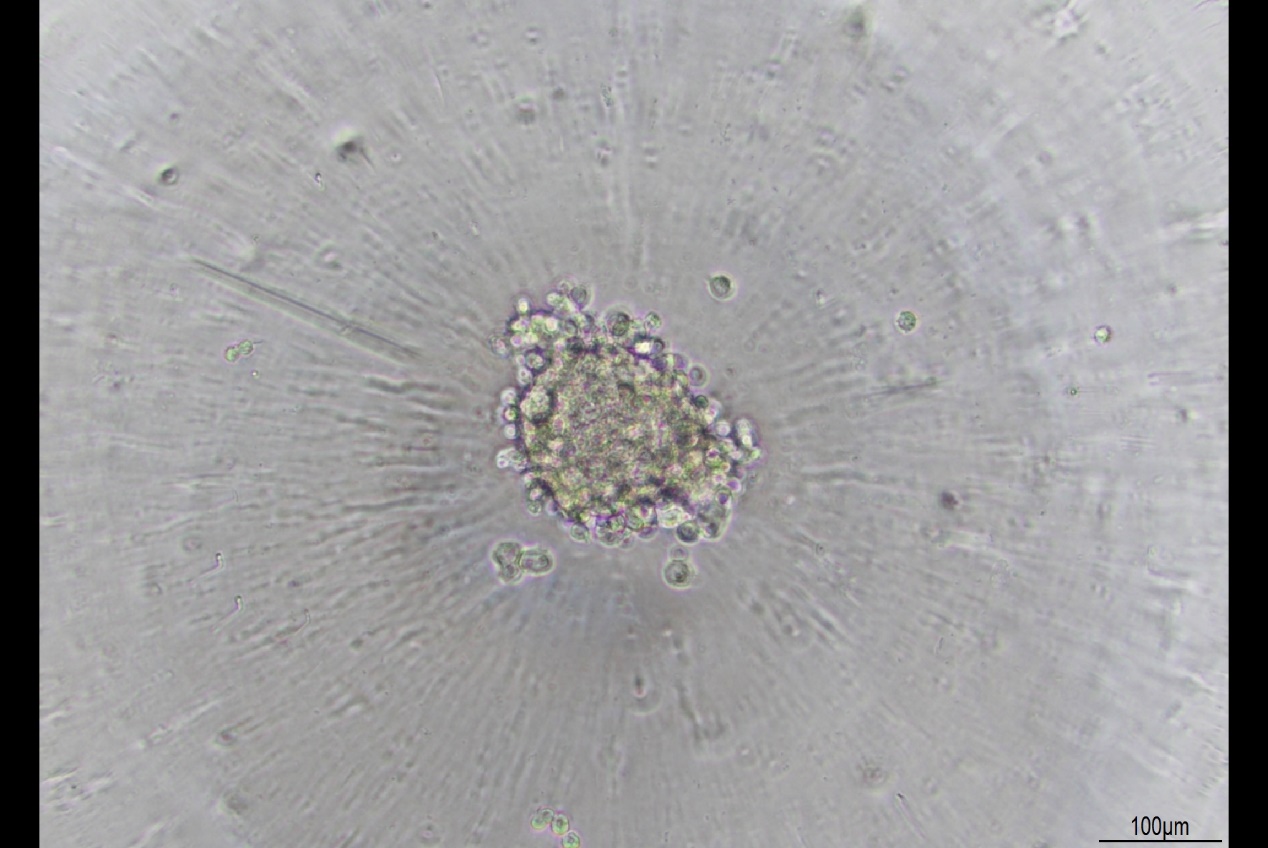
**

**DMSO DAY 6**

*
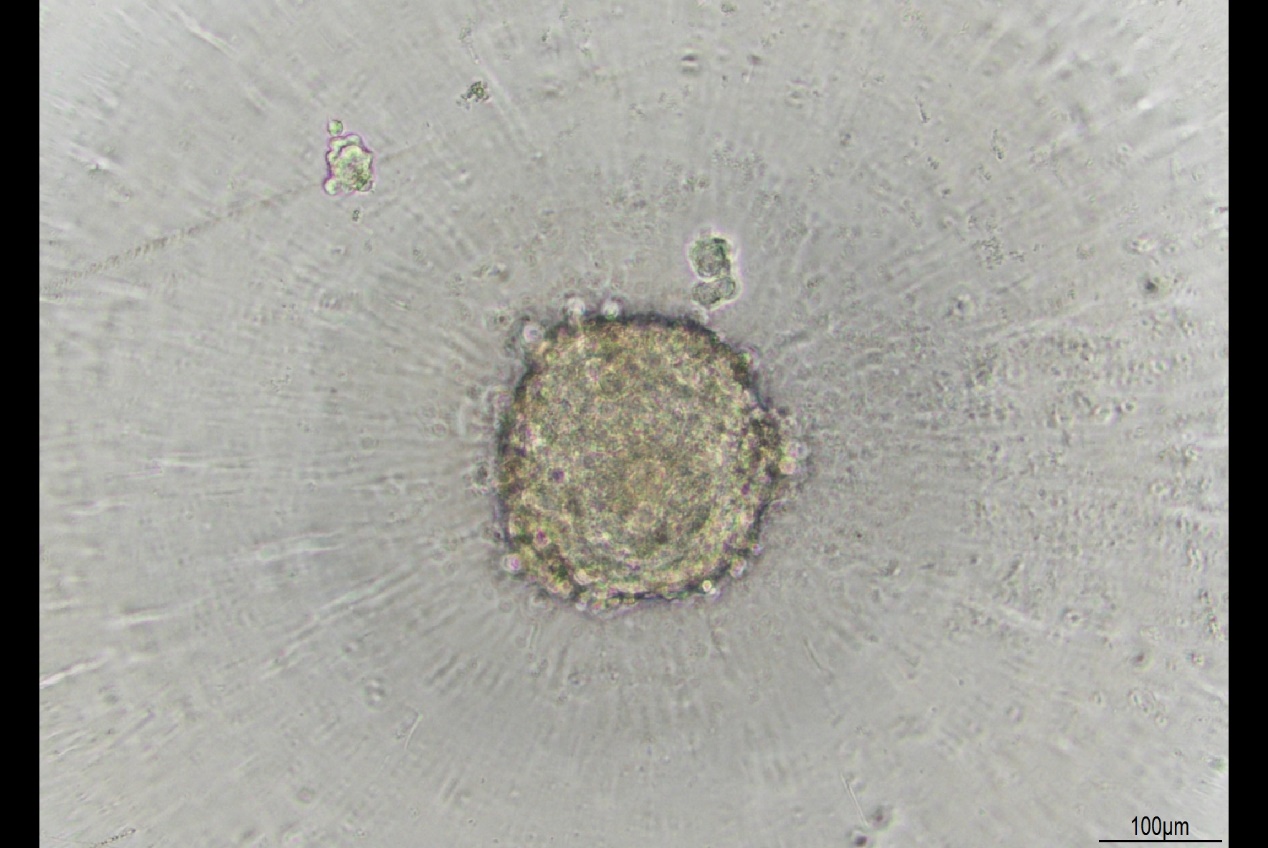
*

**DMSO DAY 12**

*
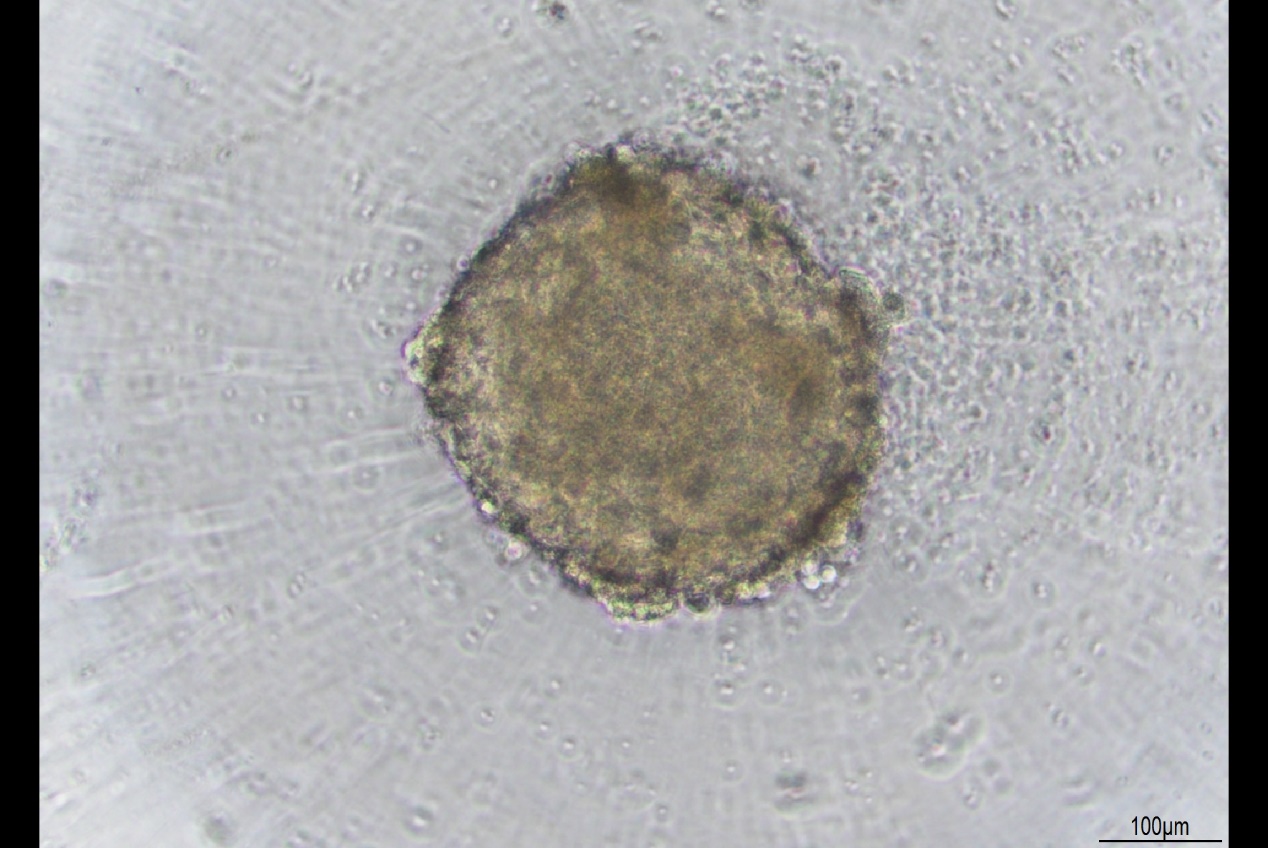
*

**PTX DAY 0**

*
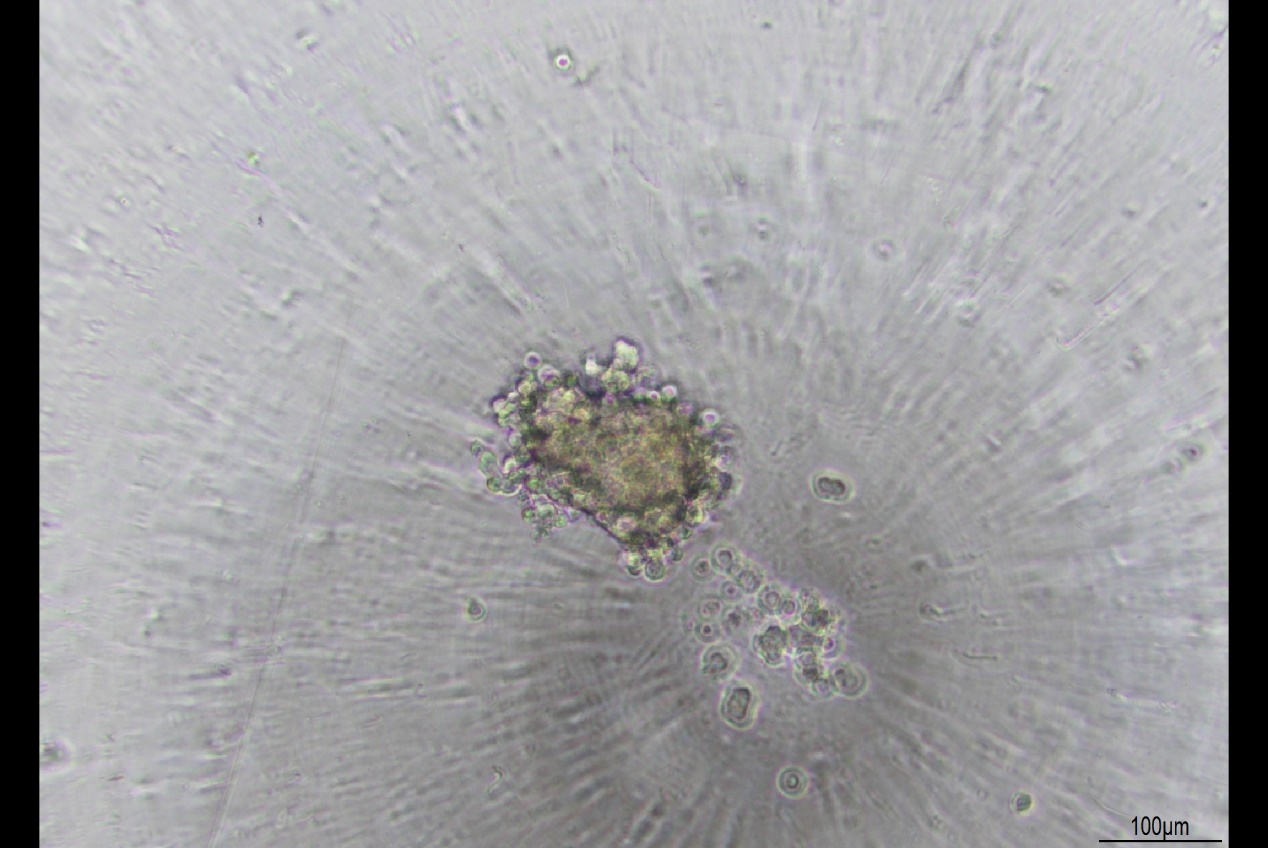
*

**PTX DAY 6**

*
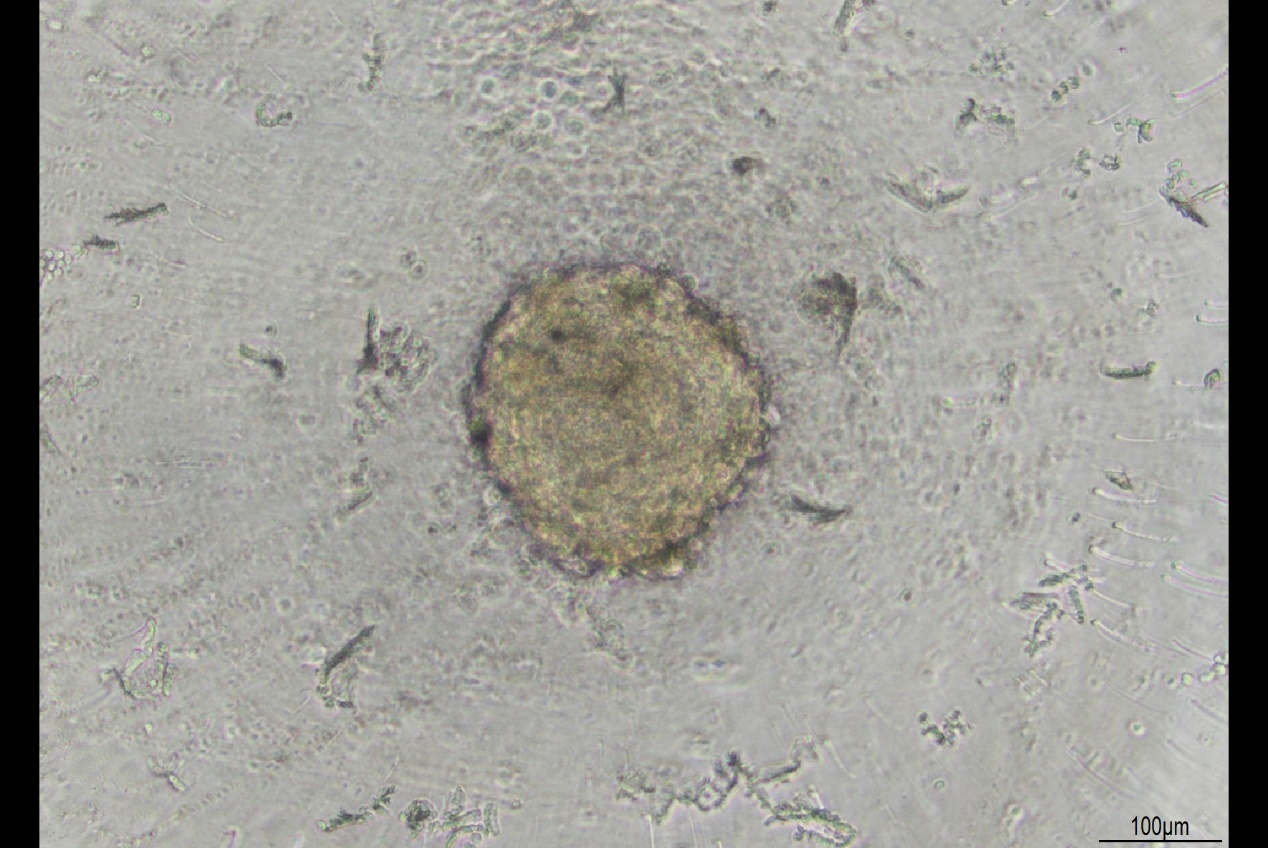
*

**PTX DAY 12**

*
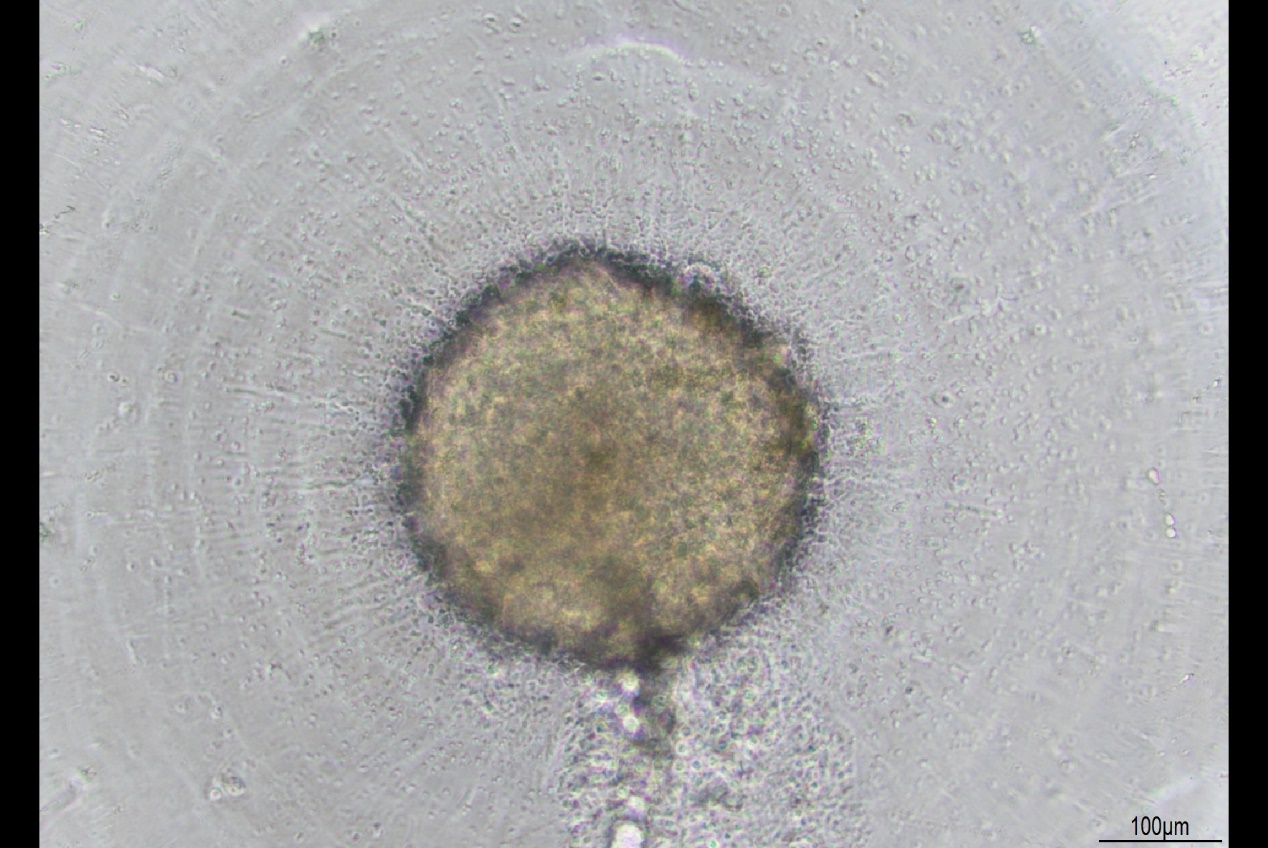
*

**GPT DAY 0**

*
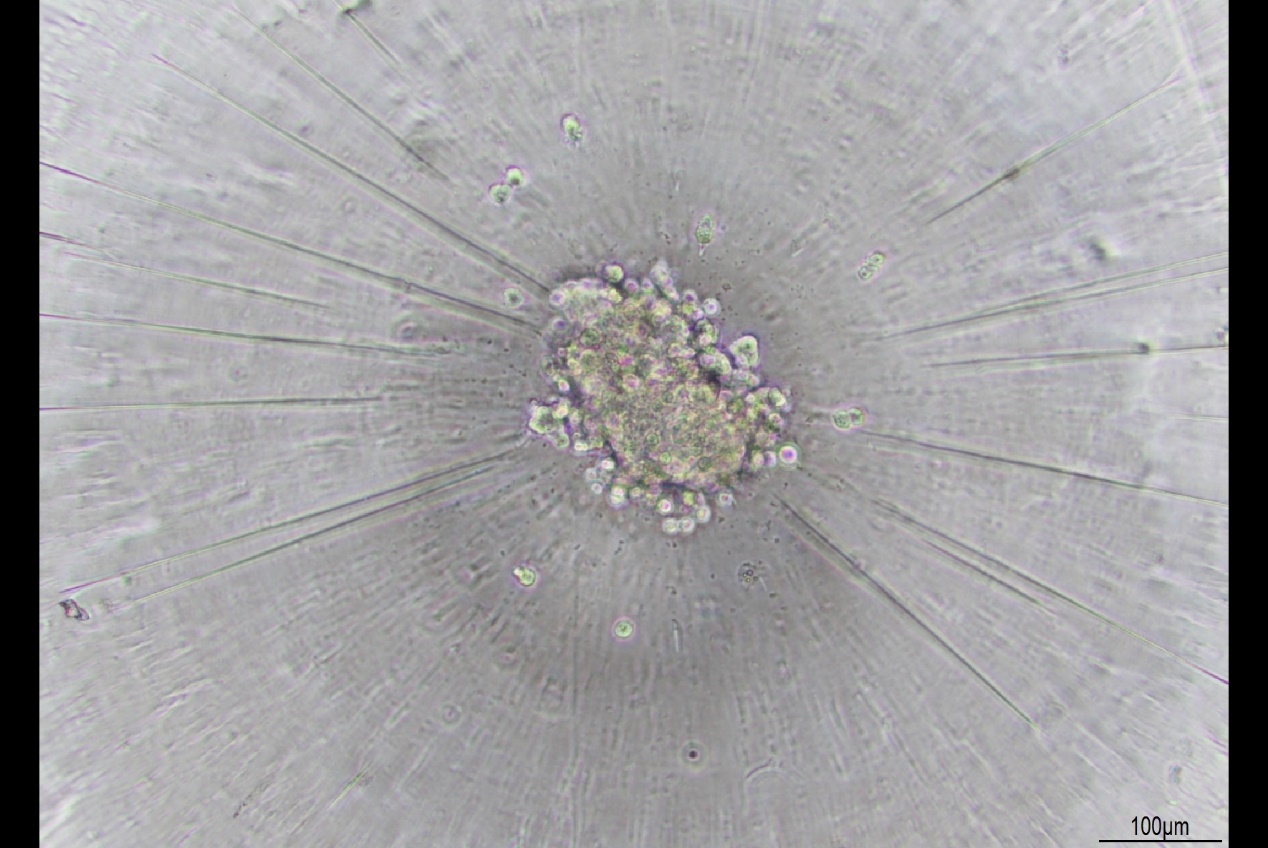
*

**GPT DAY 6**

*
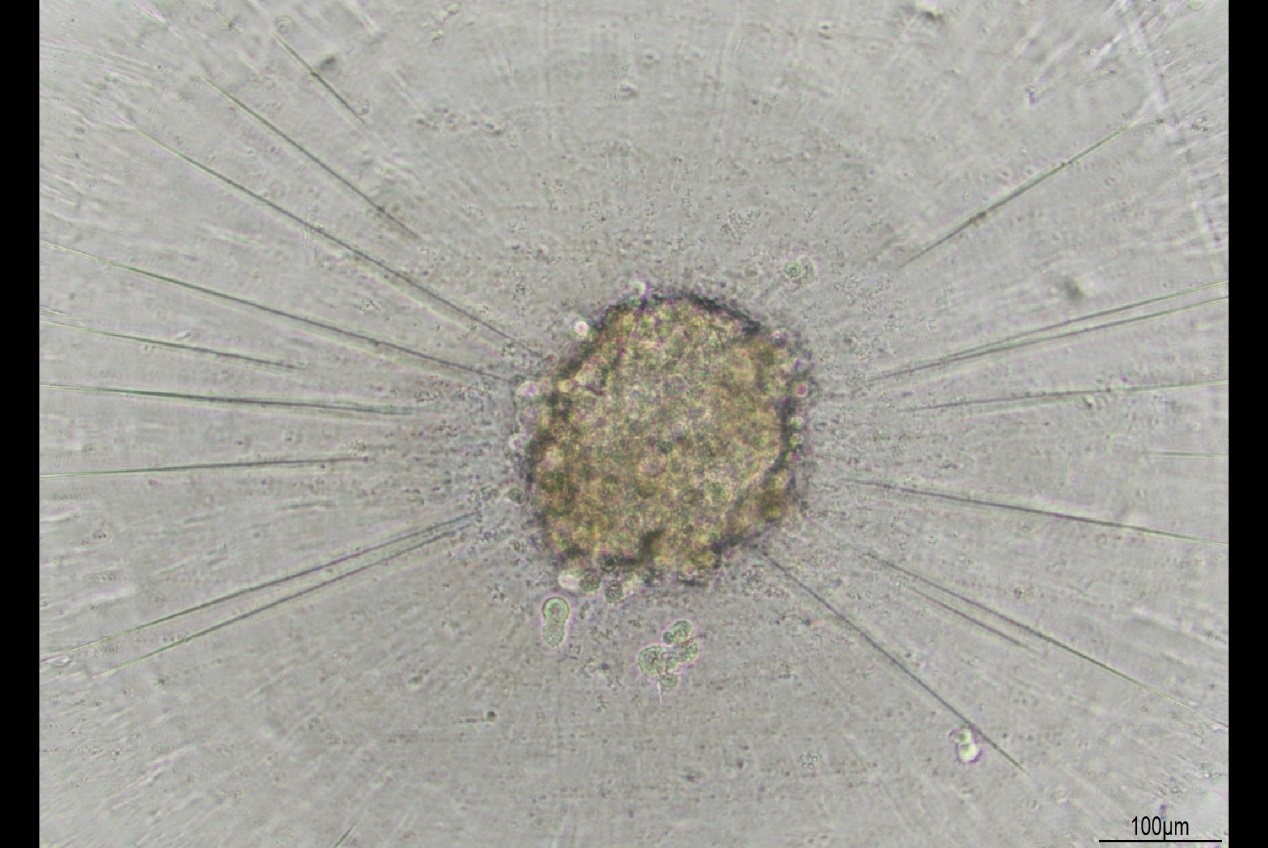
*

**GPT DAY 12**

**
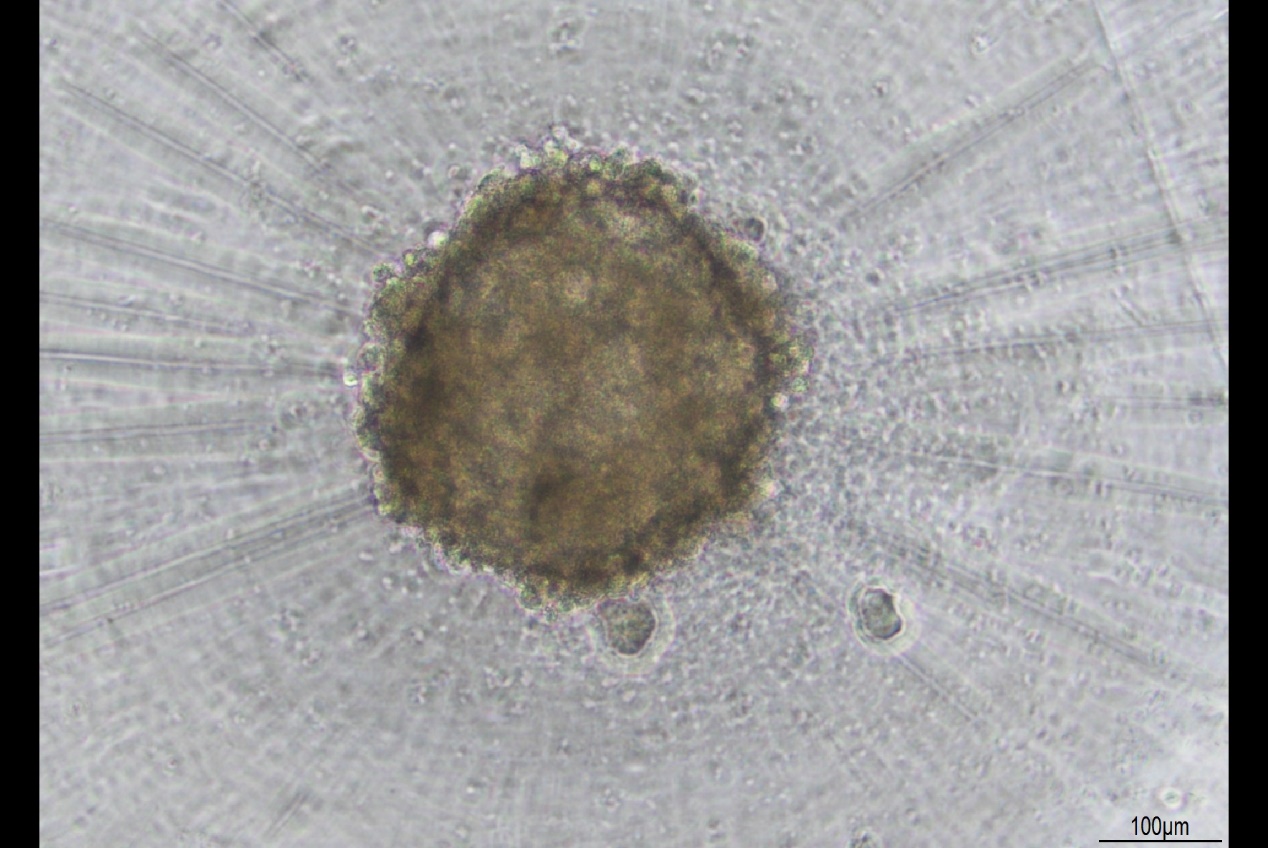
**

**PTX+GPT DAY 0**

*
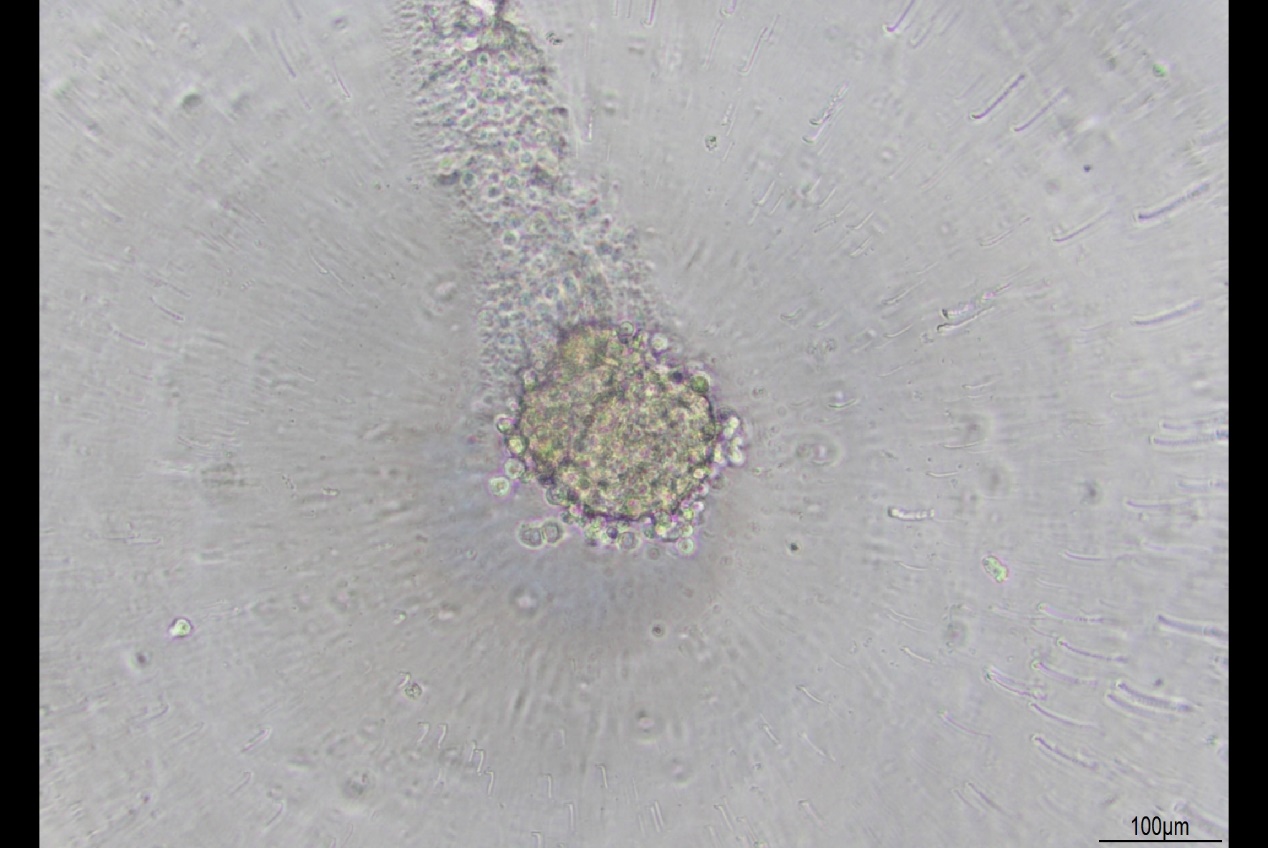
*

**PTX+GPT DAY 6**

*
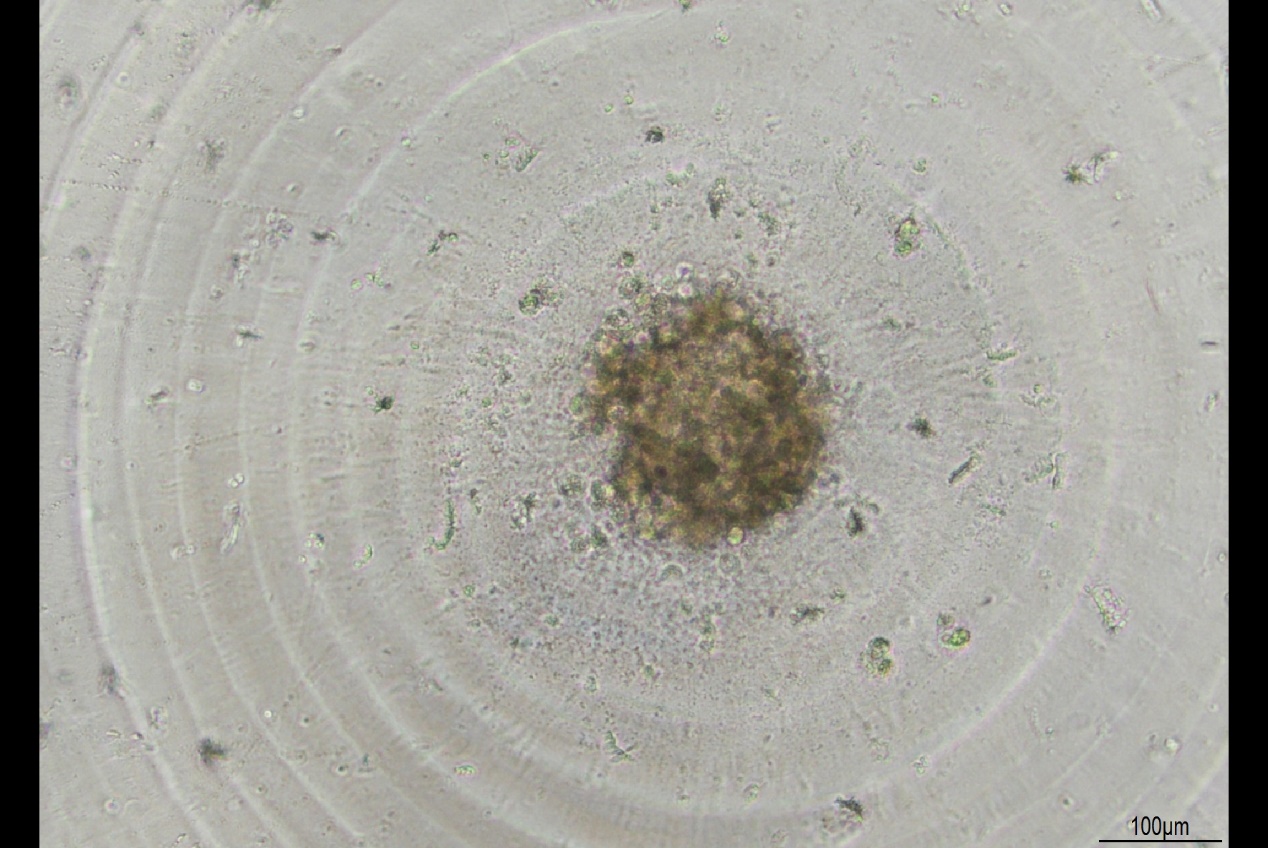
*

**PTX+GPT DAY 12**

*
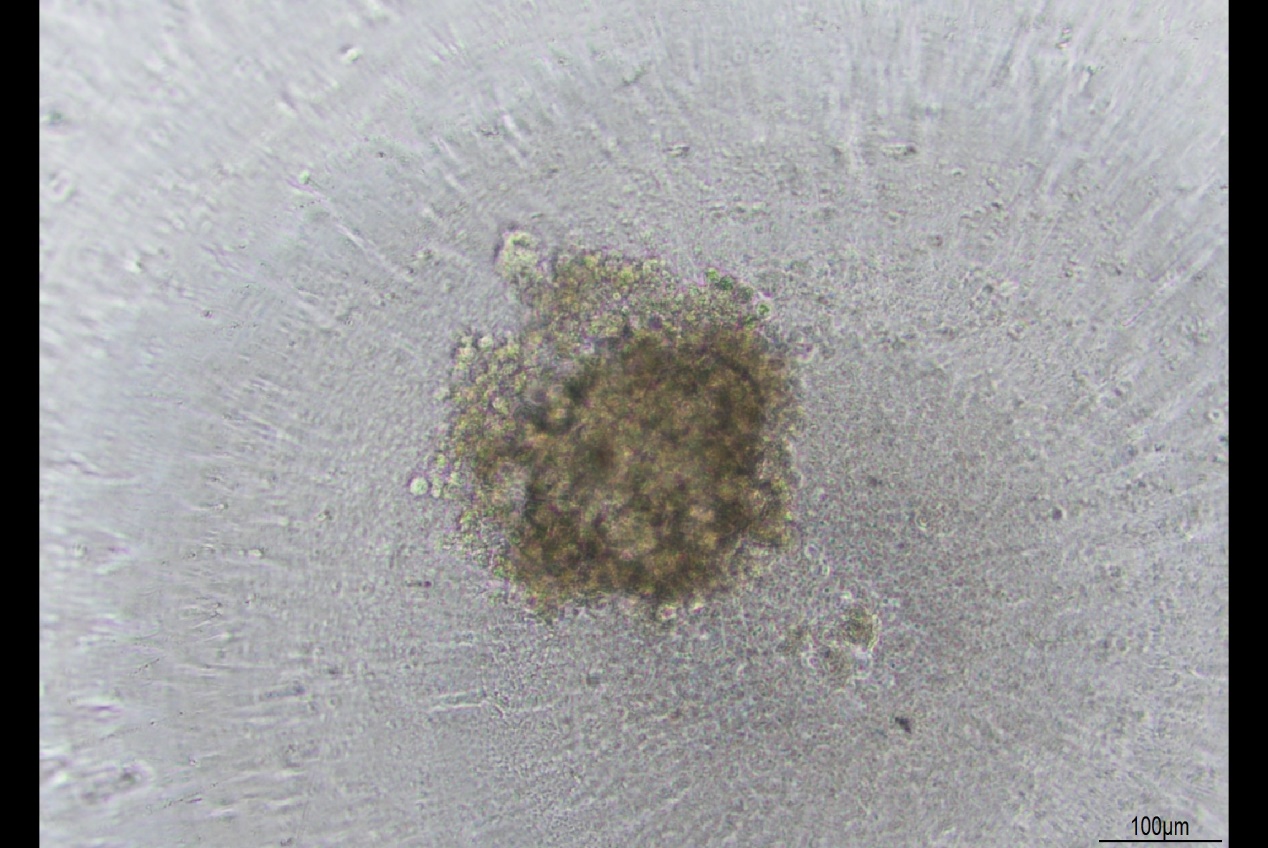
*
